# A neutralizing antibody target in early HIV-1 infection was recapitulated in rhesus macaques immunized with the transmitted/founder envelope sequence

**DOI:** 10.1371/journal.ppat.1010488

**Published:** 2022-05-03

**Authors:** Sarah Welbourn, Srirupa Chakraborty, Jie E. Yang, Anne S. Gleinich, Sailaja Gangadhara, Salar Khan, Courtney Ferrebee, Bhrugu Yagnik, Samantha Burton, Tysheena Charles, S. Abigail Smith, Danielle Williams, Rohini Mopuri, Amit A. Upadhyay, Justin Thompson, Matt A. Price, Shiyu Wang, Zhaohui Qin, Xiaoying Shen, LaTonya D. Williams, Nathan Eisel, Tiffany Peters, Lu Zhang, William Kilembe, Etienne Karita, Georgia D. Tomaras, Steven E. Bosinger, Rama R. Amara, Parastoo Azadi, Elizabeth R. Wright, Sandrasegaram Gnanakaran, Cynthia A. Derdeyn

**Affiliations:** 1 Yerkes National Primate Research Center, Emory University, Atlanta, Georgia, United States of America; 2 Theoretical Biology and Biophysics Group, Center for Nonlinear Studies, Los Alamos National Laboratory, Los Alamos, New Mexico, United States of America; 3 Department of Biochemistry, University of Wisconsin-Madison, Madison, Wisconsin, United States of America; 4 Complex Carbohydrate Research Center, University of Georgia, Athens, Georgia, United States of America; 5 Department of Epidemiology and Biostatistics, University of California San Francisco, San Francisco, California, United States of America; 6 International AIDS Vaccine Initiative, New York city, New York, United States of America; 7 Department of Biostatistics and Bioinformatics, Rollins School of Public Health, Emory University, Atlanta, Georgia, United States of America; 8 Department of Surgery, Duke University, Durham, North Carolina, United States of America; 9 Center for Family Health Research in Zambia (CFHRZ), Lusaka, Zambia; 10 Projet San Francisco, Kigali, Rwanda; 11 Department of Pathology and Laboratory Medicine, Emory University, Atlanta, Georgia, United States of America; 12 Department of Microbiology and Immunology, Emory University, Atlanta, Georgia, United States of America; Vaccine Research Center, UNITED STATES

## Abstract

Transmitted/founder (T/F) HIV-1 envelope proteins (Envs) from infected individuals that developed neutralization breadth are likely to possess inherent features desirable for vaccine immunogen design. To explore this premise, we conducted an immunization study in rhesus macaques (RM) using T/F Env sequences from two human subjects, one of whom developed potent and broad neutralizing antibodies (Z1800M) while the other developed little to no neutralizing antibody responses (R66M) during HIV-1 infection. Using a DNA/MVA/protein immunization protocol, 10 RM were immunized with each T/F Env. Within each T/F Env group, the protein boosts were administered as either monomeric gp120 or stabilized trimeric gp140 protein. All vaccination regimens elicited high titers of antigen-specific IgG, and two animals that received monomeric Z1800M Env gp120 developed autologous neutralizing activity. Using early Env escape variants isolated from subject Z1800M as guides, the serum neutralizing activity of the two immunized RM was found to be dependent on the gp120 V5 region. Interestingly, the exact same residues of V5 were also targeted by a neutralizing monoclonal antibody (nmAb) isolated from the subject Z1800M early in infection. Glycan profiling and computational modeling of the Z1800M Env gp120 immunogen provided further evidence that the V5 loop is exposed in this T/F Env and was a dominant feature that drove neutralizing antibody targeting during infection and immunization. An expanded B cell clonotype was isolated from one of the neutralization-positive RM and nmAbs corresponding to this group demonstrated V5-dependent neutralization similar to both the RM serum and the human Z1800M nmAb. The results demonstrate that neutralizing antibody responses elicited by the Z1800M T/F Env in RM converged with those in the HIV-1 infected human subject, illustrating the potential of using immunogens based on this or other T/F Envs with well-defined immunogenicity as a starting point to drive breadth.

## Author summary

A subset of HIV-1 infected individuals generates antibodies that neutralize many different viral strains. However, this process requires years of infection and has proved difficult to achieve through vaccination. The envelope proteins of the infecting HIV-1 virus likely contain features that drive neutralization breadth within an individual. To explore this, we immunized rhesus macaques using envelope proteins from two HIV-1 infected human subjects that developed either high or low neutralizing antibody levels during infection. Several animals developed neutralizing antibodies when immunized with the envelope protein derived from the high neutralizing individual. Using viral escape variants that emerged in this infected individual over time, we mapped the targets of vaccine-elicited neutralizing antibodies to a distinct loop region of the envelope protein, which was also targeted by neutralizing monoclonal antibodies isolated from both the infected individual and the immunized rhesus macaques. Modeling of the envelope protein used in the vaccine demonstrated why this loop is vulnerable to antibody attack in infection and vaccination. By recapitulating the early HIV-1 human neutralizing antibody responses in a nonhuman primate immunization model, we demonstrate remarkable similarities as well as the potential of using this approach as a starting point to drive neutralizing antibody breadth, one of the ultimate goals of vaccine design.

## Introduction

The HIV-1 envelope (Env) glycoproteins (gp) contain the determinants of entry into a host cell and are the targets of neutralizing antibodies (nAb). However, Env has heavy glycosylation that is extremely heterogeneous, particularly on the surface subunit gp120, resulting in a dynamic shield that often conceals conserved nAb epitopes [1]. A growing number of studies have established a strong link between glycosylation of the T/F Env (the antigen that likely initiates nAb) and the ensuing development of nAb breadth against diverse variants. Several studies, including our own, have shown that potential asparagine-linked glycan sites (PNGS) present in the T/F Env are an important predictor for the subsequent development of nAb breadth during infection [2–4]. A recent study conducted in a large acute infection cohort also demonstrated that higher engagement of naïve B cells with the autologous T/F Env within the first weeks of infection predicted the development of nAb breadth [5]. In RM infected by SHIVs that carry breadth-associated T/F Envs, nAb target the same regions as in the infected individuals, sometimes sharing properties at the monoclonal antibody (mAb) level, and highlighting the significance of this nonhuman primate model [6]. It has also been firmly established that HIV-1 broadly neutralizing antibodies (bnAbs) develop from autologous nAb lineages in response to ongoing viral evolution [7–11]. The various observations linking T/F Env glycosylation with neutralization breadth suggests that this prolonged process is dependent at least in part on initial immune recognition of the T/F Env. Moreover, these observations highlight the need to better understand the relationship between Env glycosylation, immunogenicity, and elicitation of nAb for vaccines. Recent studies, including our own, have provided support for the importance of glycosylation by showing that, following vaccination, nAb predominantly target strain-specific glycan hole regions even on trimeric Env immunogens [12–14]. However, these nAb could provide a foundation for moving from autologous to heterologous neutralization if these responses could be redirected to more conserved epitopes.

In a previous study, we ranked the level of plasma neutralization breadth present at 3 years after infection for 21 individuals in Zambia and Rwanda that were infected by subtype C, A, or AC recombinant HIV-1 viruses [4]. Importantly, a single T/F Env variant established infection in each case. To probe similarities and differences in antibody responses elicited by T/F Envs that were associated with neutralization breadth in our cohort, we selected Env immunogens from two individuals whose humoral immune responses exhibited marked differences in autologous neutralization and heterologous breadth [4,15]. We incorporated each T/F Env into a heterologous vector vaccination regimen using DNA, modified vaccinia Ankara (MVA), and soluble protein administered to RM. One clade C T/F Env was isolated from a newly infected individual in Zambia (Z1800M) who had the highest breadth ranking in our cohort of 21 individuals [4] as well as the highest breadth score in a much larger independent study [16]. HIV-infected subject Z1800M also developed strong autologous nAb in early infection that drove initial viral diversity in the gp120 hyper-variable domains V2, V4, and V5 and led to robust escape [4]. The other T/F Env, an AC recombinant, was isolated from a Rwandan individual, R66M, whose plasma neutralization breadth ranked #20 out of 21 individuals in our cohort. Subject R66M lacked detectable levels of plasma autologous nAb, developed lower Env diversity, and did not develop detectable neutralization breadth by 3 years [4]. We posited that vaccines based in these two Envs would elicit distinct levels of autologous nAb, and that the Z1800M Env vaccine might provide a foundation for strategically enhancing neutralization breadth. We also incorporated a comparison of each T/F Env delivered as a monomeric gp120 immunogen vs. a stabilized gp140 trimer in the protein boosts.

Here, we demonstrate that all four vaccine regimens elicited high titers of antigen-specific IgG in serum that was broadly cross-reactive against diverse Env proteins. However, only two RM, both of which received the Z1800M T/F Env DNA/MVA-gp120 regimen, developed autologous nAb. For the two neutralization-positive RM, there was no evidence of serum neutralization breadth; however, the autologous nAb from both immunized RM was dependent on the gp120 V5 region. Neutralization was abrogated by a pair of amino acid substitutions or a single amino acid deletion in V5 that were found in early plasma nAb escape variants from Z1800M. These changes also conferred resistance to an early neutralizing mAb isolated from this subject. Profiling and modeling of glycan coverage on the Z1800M T/F Env gp120 immunogen supported that the V5 region is highly exposed on the T/F Env and that this inherent feature drove nAb targeting in subject Z1800M and in two immunized RM. Finally, an expanded family of V5-dependent mAbs was isolated from one of the neutralization-positive animals and these demonstrated identical neutralization characteristics to both the RM serum and the human Z1800M mAb 1A8. The results demonstrate that the Z1800M T/F Env nAb responses elicited in immunized nonhuman primates converge with those of the HIV-1 infected human subject from which the Env was isolated, highlighting the importance of Env immunogen selection and characterization, as well as the potential for using this as a platform to drive breadth.

## Results

### Generation of HIV-1 Env vaccine components

The wildtype T/F Envs, R66M_07Mar06_EnvO20 and Z1800M_26Jun07_EnvE4, are clade A/C recombinant and clade C, respectively, and have been described previously [4,15]. The nucleotide sequences of these two T/F *env* genes were deposited into Genbank (KX983519.1 for R66M and KX983907.1 for Z1800M). The gp120 proteins used for immunization contained the wildtype sequences with a stop codon introduced into the Env open reading frame after HXB2 residue 511. Gp120 proteins were expressed and purified from 293F cells and antigenic characterization was conducted with reference bnAbs and non-neutralizing mAbs that recognize known epitopes through biolayer interferometry (BLI) [15] and a binding antibody multiple assay (BAMA) format [17] (S1, S2A and S3 Figs). Anti-HIV immunoglobulin (HIVIG) and the non-neutralizing V3 mAb 19b bound robustly to both proteins (S1A Fig). The V3 glycan bnAb PGT121 and the CD4 binding site (CD4bs) bnAb VRC01 also bound to both proteins, although weakly to Z1800M gp120, and BLI and BAMA produced comparable results for VRC01 (S1A Fig). However, the V3 glycan bnAb PGT125 and the CD4bs non-neutralizing mAb F105 bound only to the R66M gp120 (BAMA and BLI for F105), while the CD4bs bnAb CH103 bound only to the Z1800M gp120 at a low level (S1A Fig). Neither gp120 protein displayed detectable binding by the V1V2 apex bnAbs PGT145 and PG16, the gp120-gp41 interface bnAb PGT151, or non-neutralizing mAb 17b, which recognizes a CD4-induced epitope (S1A Fig). Finally, an anti-gp41 non-neutralizing mAb (7B2) and an anti-influenza hemagglutinin (HA) bnAb (CH65) did not bind to either gp120 protein, as expected.

**Fig 1:**
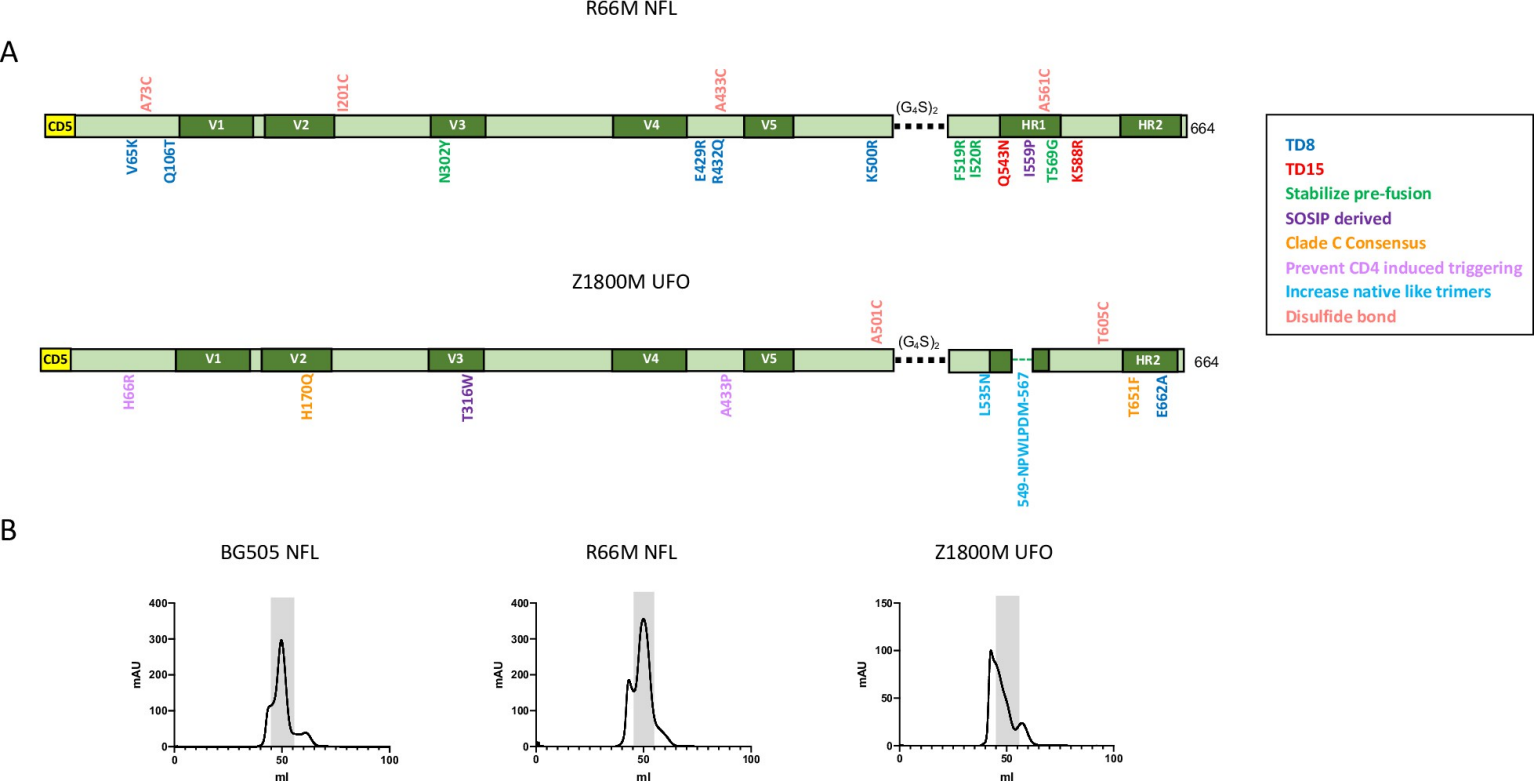
Z1800M and R66M trimers. **(A)** Linear representations of the R66M NFL and the Z1800M UFO Env trimers are shown. Cysteine residues are indicated along the top; the (G_4_S)_2_ linker is indicated by a black dashed line; the optimized gp41 region in the UFO is indicated by a green dashed line; key amino acid substitutions with HXB2 numbering are indicated along the Env open reading frame and are color-coded to indicate their putative contribution to stabilization. The CD5 leader sequence is also shown. **(B)** Size exclusion chromatography profiles are shown for the BG505 NFL trimer as a process control, the R66M NFL trimer, and the Z1800M UFO trimer. The fractions corresponding to the trimeric proteins are shaded.

To generate the R66M and Z1800M trimers, we first evaluated whether each Env contained any of the 15 BG505-associated, trimer-derived (TD) stabilizing residues that were the basis for the native flexibly linked (NFL) approach used successfully to generate gp140 trimers for several diverse HIV-1 Envs [18,19]. The R66M Env harbored 8 of the 15 TD residues (HXB2 residues D47, D49, E164, L165, V172, R308, S583, A662), suggesting that it was a good candidate for the NFL stabilization approach. The R66M NFL trimer was also designed to contain the CD5 leader sequence to improve expression, and stabilization was based on the TD-disulfide bond (CC) NFL strategy described in [18], which incorporated mutations into the trimer axis, V1V2, V3, the pre-bridging sheet, and the gp41-gp120 interface (Fig 1A). The R66M NFL contained disulfide bonds between HXB2 A73C and A561C [20] and I201C and A433C [18,19,21] and was truncated at HXB2 residue D664, prior to the membrane spanning domain, to produce a soluble gp140 [19]. Additional modifications to the R66M NFL included the SOSIP-associated HXB2 I559P substitution to enhance trimer formation and replacement of the cleavage site between gp120 and gp41, REKR, by a 2X four glycine one serine linker, (G_4_S)_2_, making this protein cleavage independent [22–26] (Fig 1A). Finally, the R66M NFL contained HXB2 residues Y302, R519, R520, and G569, which were shown to stabilize Env in the pre-fusion conformation [21].

In contrast to the R66M T/F Env, the Z1800M T/F Env contained only 2 TD residues (HXB2 residues V172 and R308). Therefore, we adopted the uncleaved prefusion-optimized (UFO) based approach described in [27,28], which was further supported by the recent generation of a well-behaved clade C C.1086 Env trimer using this approach (Sahoo et al., in press at *Cell Reports*). The Z1800M trimer contained a restructuring of a disordered region in gp41 HR1 (HXB2 547–569) to stabilize the prefusion conformation [29], as well as additional modifications in gp120, including an HXB2 A501C-T605C disulfide bond adopted from the SOSIP design, HXB2 residues R66 and P433 that prevent CD4 induced conformational changes [27], HXB2 Q170 derived from the clade C consensus sequence [27], and HXB2 N535 to increase native like trimers [30] (Fig 1A). Like the R66M NFL, this construct also contained the CD5 leader sequence, the (G_4_S)_2_ linker, and was truncated at HXB2 residue D664 (Fig 1A). Size exclusion chromatography (SEC) profiles are shown in Fig 1B, along with the BG505 NFL trimer, which was produced in parallel as a control but was not used for any of the immunizations. The BG505 and R66M NFL preparations exhibited a robust trimer-containing peak; however, this peak was much less prominent for the Z1800M UFO (Fig 1B).

**Fig 2:**
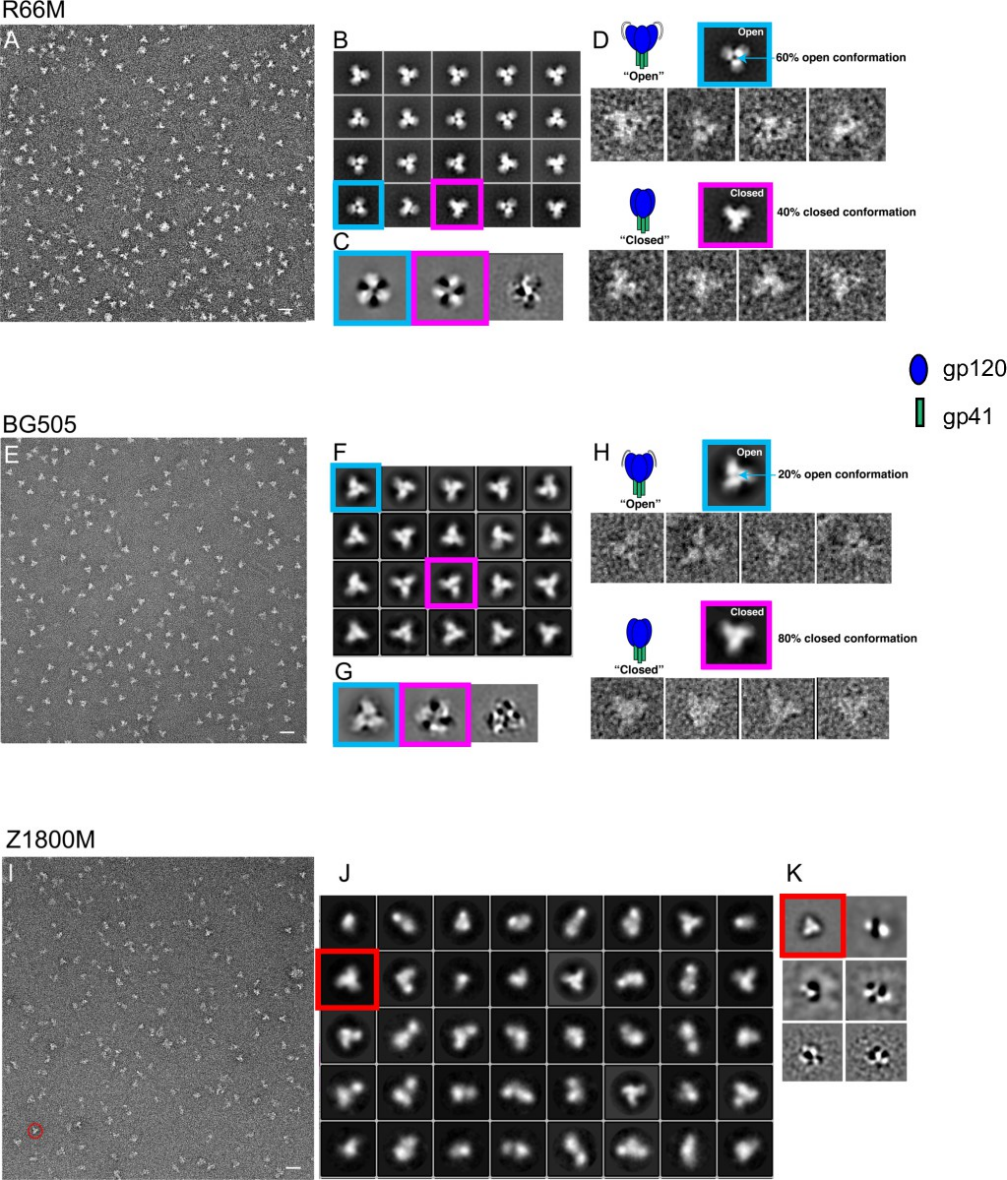
Negative stain electron microscopy analysis of Z1800M and R66M trimers. Negative stain transmission electron microscopy images **(A, E, I)** and reference-free 2D class averages **(B, F, J)** are shown for R66M, BG505, and Z1800M trimers. Representative open and closed states for the R66M and BG505 trimers are indicated by blue and magenta boxes, respectively, in **(B)** and **(F)**. Eigen vector classes of aligned particles of R66M **(C)** and BG505 **(G)** also demonstrate open and closed states. Reference-free 2D class averages of open (blue boxed) and closed (magenta boxed) representative particles of R66M **(D)** and BG505 **(H)** are shown. Z1800M trimers show a more heterogenous population **(I)**. The red circle in **(I)** and red boxes in **(J)** and in the Eigen vector classes in **(K)** highlight stable trimers. For R66M, approximately 60% open and 40% closed out of 14,000 included particles for 2D classification were observed. For BG505, approximately 20% open and 80% closed out of 12,000 included particles for 2D classification were observed. Scale bar = 20 nm.

The R66M NFL and Z1800M UFO trimers were further characterized by negative stain EM (NSEM), again using BG505 NFL as a prototype (Fig 2). The reference-free 2D class averages were used to determine their overall state. R66M and BG505 NFL trimer populations were homogeneous and well-behaved, exhibiting a classic propeller-like shape (Fig 2A and 2E). However, stabilized pre-fusion Env trimers have been shown to be conformationally flexible and can “breathe” by fluctuating between native-like closed and more open forms in an equilibrium, revealed by NSEM [31–35]. While the BG505 NFL trimers displayed a high propensity to remain closed (Fig 2F and 2H), the equilibrium of the R66M NFL trimer was shifted towards an open structure (Fig 2B and 2D). As a result, a greater proportion of partially open trimers (~60% open and 40% closed out of 14,000 included particles for 2D classification) was observed in R66M, compared to BG505 that adopted predominantly a more native closed from (~20% open and 80% closed out of 12,000 included particles for 2D classification). To further minimize the effects of negative stain grain size on the analysis, iterative multivariate statistical analysis (MSA) based reference-free 2D classification was applied to the R66M and BG505 aligned particle data sets [36]. As shown in Fig 2C and 2G, and consistent with previous reports by NSEM analysis [33,35,37], open (blue boxed) and closed (magenta boxed) eigen vector classes of aligned particles confirmed the dynamic “breathing” behavior of both constructs. In contrast to R66M and BG505, the Z1800M UFO population was heterogeneous, comprised of stable trimers but also dimers and monomers (Fig 2I–2K). While well-behaved Z1800M trimers were present and displayed a closed conformation (Fig 2J and 2K), they comprised a smaller proportion than BG505 and R66M, in which the well-formed trimers were dominant. The NSEM results were therefore consistent with the SEC profiles in demonstrating substantial variability in the degree of stabilization across these three genetically diverse HIV-1 Envs.

We also characterized the antigenicity of the R66M and Z1800M trimers using BAMA and BLI (S1B, S2B and S3 Figs), as was done for the gp120 proteins (S1A Fig). Both the R66M NFL and the Z1800M UFO were bound robustly by HIVIG and the V3 non-neutralizing mAb 19b, like the gp120 proteins. The V3 glycan bnAb PGT125 and the V1V2 apex bnAb PG16 also bound to both R66M and Z1800M trimers, but more strongly to the former. Other bnAbs and non-neutralizing mAbs against the CD4bs, V3, and gp41 also bound both trimers to varying degrees, with BAMA and BLI producing similar results when tested in parallel. The CD4bs bnAb CH103 bound only to Z1800M, as was the case for the gp120 proteins. The V1V2 apex bnAb PG9 also bound to R66M, while the V1V2 apex bnAb PGDM1400 bound to Z1800M. The V1V2 apex bnAb PGT145, the gp120-gp41 interface bnAb PGT151, as well as the CD4-induced non-neutralizing mAb 17b and the anti-influenza bnAb CH65 did not bind to either trimer. In addition, the V3 glycan bnAb PGT128 failed to bind to Z1800M. For comparison, the BG505 NFL was tested with a subset of the bnAbs and mAbs using BLI only (S1C and S2B Figs). In contrast to the R66M and Z1800M trimers, the apex bnAb PGT145 bound strongly to the BG505 NFL trimer. Conversely, F105 bound weakly to the Z1800M and R66M trimers but not at all to BG505. All three trimers were recognized by 447-52D, indicating some exposure of the V3 domain. The CD4-induced non-neutralizing mAb 17b did not bind to any of the three trimers. Overall, the SEC profiles, NSEM analysis, and antibody binding studies supported the presence of trimers in the R66M NFL and Z1800M UFO preparations, both of which displayed a combination of trimer-dependent and non-trimer dependent neutralizing and non-neutralizing epitopes. However, the BG505 NFL trimer exhibited desirable features that distinguished it from R66M and Z1800M, including a higher level of homogeneity, a higher proportion of trimers in a closed state, strong binding by the V1V2 apex bnAb PGT145, and lack of binding by the CD4bs non-neutralizing mAb F105.

### Immunization of RM with heterologous prime/boost R66M and Z1800M T/F Env vaccines

We next tested whether vaccines based on the Z1800M and R66M T/F Envs could recapitulate features of nAb elicited in the human subjects from whom they were isolated. Stabilized Env trimer immunogens such as BG505 SOSIP have demonstrated the ability to elicit autologous nAb; however, these proteins do exhibit differences from the native Env, including altered glycosylation [38,39] and the elicitation of non-neutralizing antibodies that target neo-epitopes created by the exposed trimer base [12,40]. We reasoned that other native-like Env forms could be useful immunogens in this regard, including membrane-bound on the surface of cells and virus like particles. We therefore generated recombinant vectors to co-express the wildtype T/F Envs with SIVmac239 Gag, facilitating the production of virus like particles and cell surface Env expression *in vivo* [41,42]. Furthermore, our previous study demonstrated DNA, MVA, and protein vaccination generated higher quality serum nAb responses than six other modalities [43]. The co-expression vectors were comprised of recombinant DNA plasmids that expressed SIVmac239 Gag with each HIV-1 T/F Env gp160 and MVA viral vectors that expressed each Env gp150. Characterization of Gag and Env expression by the recombinant vectors is shown in S4 Fig.

**Fig 3:**
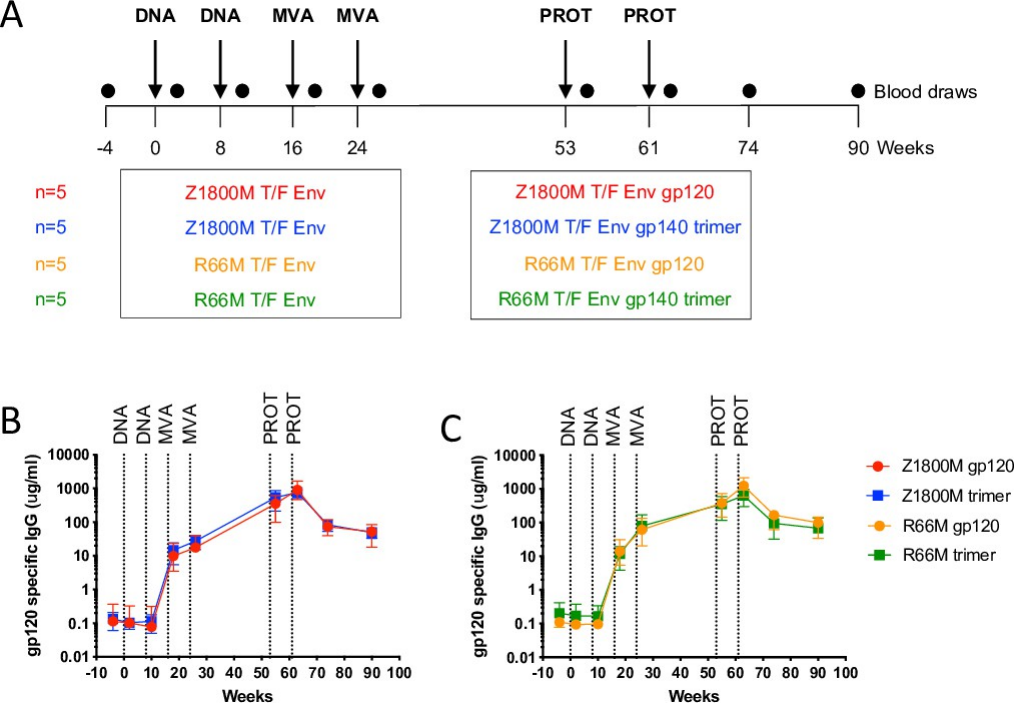
Immunization regimen and antigen-specific serum IgG responses. **(A)** A representation of the immunizations, time scale, and vaccination groups are shown. The four vaccine groups consisted of 5 RM immunized intramuscularly with DNA expressing SIVmac239 Gag and the indicated HIV-1 Env, R66M or Z1800M T/F Env, at weeks 0 and 8; MVA expressing SIVmac239 Gag and the R66M or Z1800M T/F Env at weeks 16 and 24; and the R66M or Z1800M T/F Env as a stabilized gp140 trimer or gp120 protein in Adjuplex at weeks 53 and 61. Blood was collected at baseline (week -4) and weeks 2, 10, 18, 26, 55, 63, 74, and 90. Serum IgG with specificity for the autologous T/F Env gp120, Z1800M **(B)** and R66M **(C)**, was quantified by ELISA. Serial dilutions of serum from RM in each of the four vaccine groups were tested at weeks -4, 2, 10, 18, 26, 55, 63, 74, and 90. The group medians with standard error of the mean are shown in μg/ml, based on a RM IgG standard curve. For samples where both replicates fell below the limit of detection, a value of 0.078 was used. If only one replicate fell below the limit of detection, the other value was used. All samples were measured in duplicate wells and in two independent experiments.

The vaccines were administered to young adult male (n = 17) and female (n = 3) RM through intramuscular injection. The regimen, shown in Fig 3A, consisted of two immunizations with the Env-Gag co-expressing DNA plasmid at weeks 0 and 8; two immunizations with the Env-Gag co-expressing MVA at weeks 16 and 24; and two immunizations with the Z1800M or R66M T/F Env protein in Adjuplex at week 53 and 61, as either monomeric gp120, or as the stabilized UFO/NFL gp140 trimers described above [18–21,27,30,44]. The vaccination groups initially consisted of Z1800M Env DNA/MVA/gp120 (n = 5), Z1800M Env DNA/MVA/gp140 trimer (n = 5), R66M Env DNA/MVA/gp120 (n = 5), and R66M Env DNA/MVA/gp140 trimer (N = 5) (Fig 3A). However, one RM in the Z1800M DNA/MVA/gp120 group did not complete the immunization series due to circumstances unrelated to the protocol.

Antigen specific serum IgG was measured by binding to the matching gp120 protein in ELISA and was evaluated at two weeks after each immunization (Fig 3B and 3C). All four vaccines elicited robust levels of autologous gp120-specific IgG that were similar across groups. Antigen-specific IgG levels were below the limit of detection at baseline and following the two DNA immunizations, measured at weeks -4, 2, and 10. An increase of approximately two logs occurred after the first MVA, which was evaluated in week 18 serum. The second MVA provided a moderate boost apparent in week 26 serum that was slightly higher for R66M Env vaccinated animals than for those that received the Z1800M Env-based vaccines. The first and second protein boosts provided further increases in serum collected at weeks 55 and 63, with no significant differences noted across the vaccines (Kruskal-Wallis test with multiple pairwise comparisons, p>0.05). Thus, each vaccine elicited a robust serum IgG response quantified using the autologous gp120 that was not distinguishable by the Env variant or the form of protein boost.

### Profiling of vaccine-elicited serum IgG antibodies

We next conducted a more extensive profiling of serum IgG using BAMA to evaluate recognition of globally diverse Envs. Antibody binding to a previously described panel consisting of 8 gp120, 8 gp140, and 16 V1V2 scaffold antigens representing clades A, B, CRF01_AE, CRF07_BC, and C from different geographic regions was measured, in addition to the 4 immunogen proteins [17]. This panel had previously facilitated the identification of distinguishing features of IgG antibody breadth and heterogeneity across vaccines [17]. For all four vaccines, binding to the Env antigens was not reliably detected until week 18, two weeks after the first MVA immunization (S5 Fig), consistent with the appearance of antigen specific IgG (Fig 3B and 3C). Binding to several V1V2 antigens was not detected until week 26 in 11 RM that represented all vaccine groups (S5A Fig). There was no clear preference for binding to test antigens of a particular subtype (S5B Fig). Overall, by week 26, all immunized RM had developed serum IgG antibodies capable of recognizing diverse Env gp120/gp140/V1V2 antigens, with increases following the protein boosts, culminating in the highest magnitude at week 63. When the serum IgG binding data was analyzed by comparing MFI across the four vaccination groups, taking into consideration 4–5 RMs per 4 vaccine groups, 7 time points (including baseline), and binding to 31 panel Env antigens (data from one antigen was excluded due to missing values) and the four immunogen proteins, the antibody recognition patterns elicited across vaccines were similar. Out of 140 vaccine group-Env antigen combinations, only 3 emerged with evidence of a significant difference with a p-value less than 0.05. The R66M DNA/MVA/gp120 immunized group exhibited overall lower binding to the subtype B gp70_B.CaseA_V1V2 protein compared to the other groups (β = -2.62, p = 0.01). The Z1800M DNA/MVA/gp120 immunized group exhibited lower recognition of the AC recombinant R66M gp120 and the subtype B WITO4160.gp140C proteins compared to the other groups (β = -1.98, p = 0.02; β = -1.66, p = 0.02). However, these modest differences did not indicate a distinct pattern of recognition that could be explained by immunogen, subtype, or form of Env binding antigen. Overall, the magnitude, kinetics, and recognition capability of serum IgG antibodies elicited by the four vaccines were similar.

### Analysis of serum neutralization

**Fig 4:**
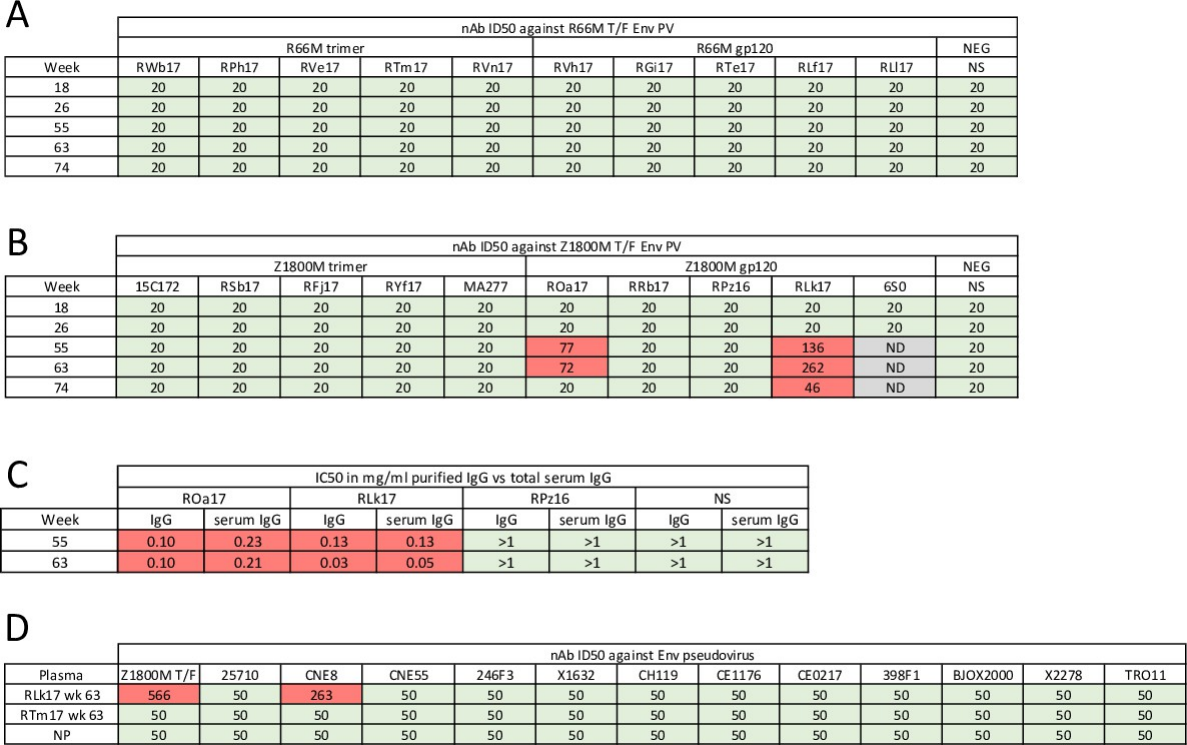
Characterization of immunized RM serum neutralization. **(A-B)** Neutralization activity was measured against the autologous T/F Env PV, Z1800M or R66M, with heat-inactivated, serially diluted serum using the TZM-bl assay. Neutralization was evaluated at weeks 18, 26, 55, 63, and 74, corresponding to two weeks post MVA immunizations, two weeks post protein immunizations, and 13 weeks after the final protein immunization. Green boxes indicate that an ID_50_ titer could not be calculated using 1:20 as the highest dilution. Red boxes show a positive ID_50_ titer. Gray boxes indicate that the serum was not evaluated (ND). **(C)** For neutralization positive week 55 and 63 post-protein serum samples from ROa17 and RLk17, and neutralization negative week 55 and 63 post-protein samples from RPz16, IgG was purified from the serum and tested for neutralization. The results shown are IC_50_ titers calculated using the quantified amounts of purified IgG and total IgG present in serum as measured by ELISA. The infectivity curves corresponding to purified and total serum IgG IC_50_ values from panel C are shown in S6 Fig. NS represents serum or IgG from a naïve RM in **(A-C)**. **(D)** Week 63 plasma from neutralization positive Z1800M gp120-immunized RLk17 was tested for neutralization activity against a global panel of envelope PV [48]. Plasma serial dilutions began at 1:50 and ID_50_ values are shown. A value of 50 indicates the highest concentration of plasma tested without 50% inhibition. Naïve RM plasma (NP) and week 63 plasma from a non-neutralizing animal (R66M trimer immunized RTm17) were used as negative controls. All experiments had duplicate wells and were repeated at least twice independently.

We next assessed nAb activity in serum against pseudoviruses (PV) expressing the autologous T/F Env, Z1800M or R66M, using the TZM-bl assay. Serum samples collected at two weeks after the MVA and protein immunizations (weeks 18, 26, 55, and 63) and 13 weeks after the final immunization (week 74) were serially diluted and evaluated. For the R66M DNA/MVA/gp120, R66M DNA/MVA/trimer, and Z1800M DNA/MVA/trimer vaccination groups, nAb was low to undetectable at all time points tested, and an ID_50_ titer could not be calculated with the least diluted serum concentration of 1:20 (Fig 4A and 4B). In contrast, serum samples from two of the four RM in the Z1800M DNA/MVA/gp120 immunized group were positive for nAb at weeks 55 and 63, following the protein boosts. The ID_50_ titers were moderate for RM ROa17, 1:77 and 1:72 respectively (Fig 4B), and it was interesting that the titer did not increase even though the second protein immunization had a clear boosting effect of the gp120-specific IgG (Fig 3B). The ID_50_ titer for RM RLk17 was higher, reaching 1:136 and 1:262 following each protein boost. At week 74, the nAb titer for ROa17 had waned to below detection, while for RLk17 was modest at 1:46. Overall, half of the Z1800M DNA/MVA/gp120 immunized animals developed autologous neutralizing antibodies.

We next tested whether IgG purified from the serum of ROa17, RLk17, and another RM from the same vaccine group that did not develop serum autologous nAb activity, RPz16, could mediate the observed neutralization. Using concentrations of purified IgG that were normalized to the IgG concentration of the serum, ROa17 and RLk17 nAb activity at weeks 55 and 63 was recapitulated (Figs 4C and S6). We also confirmed that the Z1800M T/F Env had tier 2 neutralization phenotype, although this seemed likely because this Env was derived directly from the plasma of an HIV-1 infected individual during the antigen-positive, antibody-negative acute phase through single genome PCR amplification (SGA) [4]. The general neutralization susceptibilities of Z1800M and R66M T/F Envs were characterized using a panel of bnAbs/mAbs with known epitopes at 5 μg/ml and HIV+ patient plasma samples at a 1:100 dilution, alongside Envs that had been previously characterized (398F1, tier 1.8/2; CNE55, tier 2/3; 93MW965.26, tier 1A; SHIV1157ipd3N4, tier 2; SHIV1157ipd3N4 Env G.10, tier 2) [43,45–48] (S7 Fig). We included mAb 17b, which recognizes a CD4-induced epitope; bnAb 447-52D, which recognizes an epitope in V3; HIVIG; five HIV+ plasma samples from Rwanda; 5 HIV+ plasma samples from Zambia; and two plasma pools, one from Rwanda and one from Zambia. The tier 1 Env 93MW965.26 was potently neutralized by the mAbs, HIVIG, and all plasma samples (S7 Fig). The Envs 398F1 and SHIV1157ipd3N4 EnvG.10 PV, both among the more sensitive tier 2 Env variants, were less susceptible, while CNE55 and the SHIV1157ipd3N4 virus exhibited an even higher level of resistance. The Z1800M and R66M T/F Envs exhibited the highest level of resistance in this experiment. Using a two-way ANOVA with Dunnett’s correction for multiple comparisons, we found that all Envs were statistically different from the tier 1 Env 93MW965.26 (all p<0.0001). Thus, the Z1800M and R66M T/F Envs exhibited neutralization resistance typical of globally circulating, patient-derived variants arguing that bona fide, tier 2 autologous neutralizing IgG was elicited in the two RM that were immunized with the by Z1800M DNA/MVA/gp120 vaccine. We also tested RLk17 week 63 plasma for neutralization of a tier 2 global Env panel [48]. Except for modest inhibitory activity against Env CNE8, which is among the more sensitive tier 2 Envs, there was no evidence of neutralization breadth (Fig 4D).

### Immunized RM serum neutralization is dependent on the V5 loop like the infected subject Z1800M

**Fig 5:**
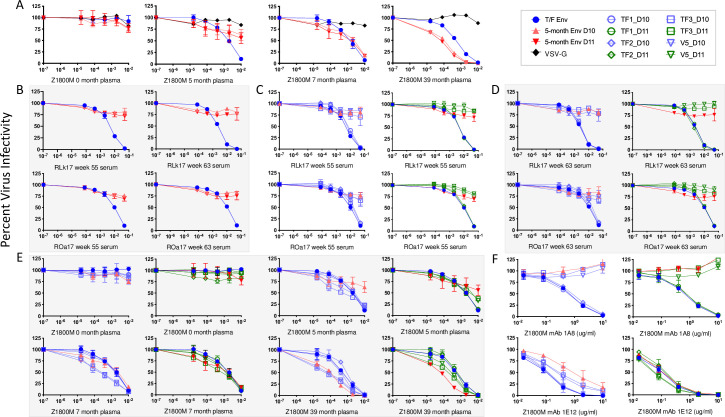
Neutralization mapping of immunized RM sera, and autologous plasma and mAbs from HIV+ subject Z1800M. **(A)** Plasma from HIV+ subject Z1800M collected close to the time of infection (0-month) and longitudinally at 5, 7, and 39 months and **(B)** week 55 and 63 serum from neutralization positive RMs RLk17 and ROa17 were tested against PV expressing the Z1800M T/F Env and 5-month Envs D10 and D11. RLk17 and ROa17 week 55 and 63 serum **(C and D)**, Z1800M longitudinal patient plasma **(E)**, and Z1800M derived mAbs 1A8 and 1E12 **(F)** were tested against Env chimeras and mutants generated in the T/F Env. The TF1, TF2, and TF3 constructs correspond to fragments from D10 (lavender) or D11 (green) that were introduced into the T/F Env background (Fig 6B). The V5 constructs correspond to the changes in D10 and D11 V5 loops (Fig 6A) that were introduced into the T/F Env. Each graph represents percent viral infectivity on the y axis and plasma/serum/mAb dilution or concentration on the x axis on a log10 scale. All experiments had duplicate wells and were repeated at least twice independently, and error bars indicate the standard deviation.

**Fig 6:**
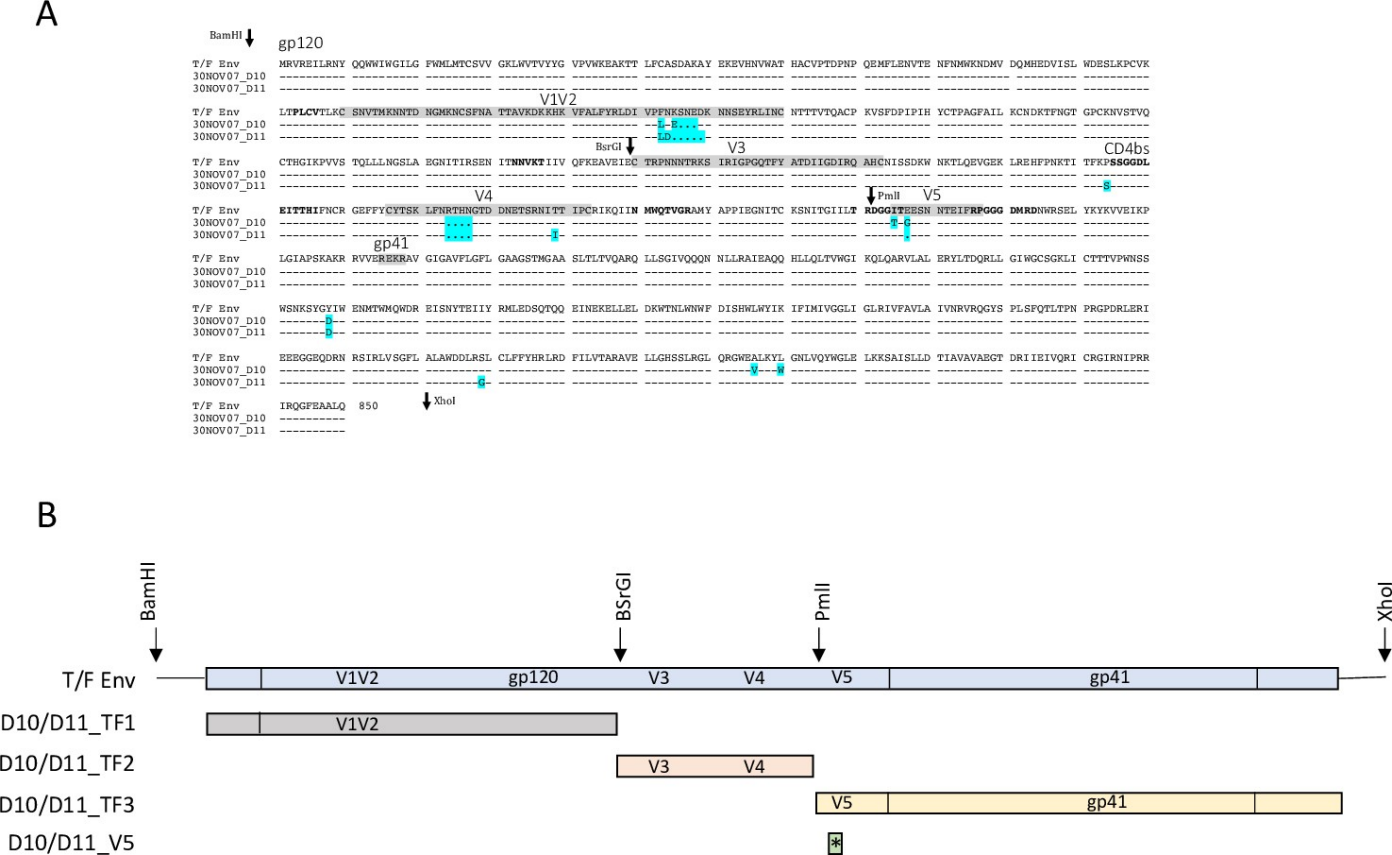
Sequence differences and chimeras based on T/F Env and escape Envs. **(A)** Amino acid sequence alignment of the Z1800M T/F Env and the 5-month neutralization escape Envs D10 and D11. Dashes indicate conserved residues relative to the T/F Env. Dots indicate deleted residues and amino acid differences from the T/F Env are indicated by cyan highlighted. Key Env regions are shaded and labeled. The positions of restriction sites used to generate chimeras shown in panel B are indicated with arrows. **(B)** The scheme for construction of the chimeras is shown. The restriction sites and fragments that were replaced in the T/F Env using D10 and D11 sequences are shown. The V5 constructs contained only the changes present in V5, which were two amino acid differences in D10 and one amino acid deletion in D11.

Focusing on the two RM that developed autologous nAb against the Z1800M T/F Env, we tested two Envs, Z1800M_30Nov07_D10 (D10; Genbank KX983921) and Z1800M_30Nov07_D11 (D11; Genbank KX983922) that were previously isolated from human subject Z1800M at 5 months after infection [4]. These Envs were much less susceptible to neutralization by the contemporaneous 5-month Z1800M plasma than the T/F Env, but the patient’s nAb evolved to neutralize them at later time points (Fig 5A). Interestingly, Envs D10 and D11 were also resistant to neutralization by week 55 and week 63 serum from RLk17 and ROa17 (Fig 5B). A comparison between the Z1800M T/F Env amino acid sequence and the two 5-month Envs, D10 and D11, revealed changes concentrated in gp120 V2, V4, and V5, as well as in the gp41 immunodominant loop and cytoplasmic tail (Fig 6A). To home in on the potential targets of neutralization, we created chimeras in which restriction fragments from the 5-month Envs D10 and D11 were exchanged into the Z1800M T/F Env background (Fig 6B). The first chimeras, D10_TF1 and D11_TF1, differed from the T/F Env only in V2. The second set, D10_TF2 and D11_TF2, differed by one residue near the CD4 binding loop as well as a deletion and substitution that removed N-linked glycan motifs in V4. The third set, D10_TF3 and D11_TF3, contained changes in V5 and gp41. When we tested PVs expressing the Env chimeras for neutralization susceptibility to RLk17 and ROa17 week 55 sera (Fig 5C) and week 63 sera (Fig 5D), we found that the TF1 and TF2 chimeras were neutralized with potency like wildtype. In contrast, the TF3 chimeras were as resistant to neutralization as the parental 5-month D10 and D11 Envs. These results indicated that vaccine-elicited nAb in RLk17 and ROa17 serum were impacted by the 5-month changes in V5 and/or gp41. We created and tested mutants, D10_V5 and D11_V5, that contained only the changes in V5: isoleucine to threonine at position 445 (HXB2 residue 459a) and glutamic acid to glycine substitution at position 447 (HXB2 residue 460) in D10_V5 and deletion of the glutamic acid at position 447 in D11_V5 (Fig 6A). Fig 5C and 5D show that the V5 mutants were completely resistant to neutralization by RLk17 and ROa17 serum, unequivocally demonstrating that nAb is dependent on residues in V5. Negative control RM serum lacked nAb against any of the constructs, as did serum from a nAb-negative RM from the same vaccination group (S8A Fig).

We also tested the CD4bs bnAb VRC01 against the chimera and mutant panel. While this bnAb neutralized all constructs, it inhibited D10, D10_TF3, and D10_V5 more potently that the T/F Env, indicating that the changes in V5 increased exposure of the VRC01 epitope within the CD4bs (S8B Fig). In contrast, D11 was less sensitive to VRC01 than the T/F Env, but the determinants of this phenotype were not as clear. We also determined the ability of longitudinal plasma samples from HIV-1 infected subject Z1800M to neutralize the chimeras and mutants (Fig 5E). Overall, viral escape from plasma neutralization was complex and could not be mapped to a single region of D10 or D11. However, the D11_TF3 and D11_V5 PV were more resistant than the T/F Env to neutralization the 5-month plasma and were more sensitive to the 39-month plasma, indicating that nAb was influenced by changes in V5 or a proximal epitope during infection (Fig 5E). On the other hand, D10, D10_TF3, and D10_V5 exhibited increased susceptibility to neutralization, particularly at the later time points. It is interesting to speculate that the autologous escape pathway involving two V5 amino acid substitutions (D10) was less advantageous than the amino acid deletion (D11) because the former also created a vulnerability by exposing the CD4bs.

### Isolation of neutralizing monoclonal antibodies from Z1800M

In addition to longitudinal Envs isolated at 5-months from human subject Z1800M, mAbs were previously isolated at 7-months from B cells that were single cell sorted using the T/F Env gp120 as a probe [4]. Of 64 mAbs tested here, three potently neutralized the Z1800M T/F Env PV (S9A Fig). We reasoned that we could use the mAbs from natural infection to refine our understanding of the vaccine-elicited serum nAb. Two of the human mAbs, 1A8 and 2H10, were derived from immunoglobulin variable domain heavy (VH) and light (VL) chain genes VH4.39/Vλ3.21, had very similar 13 amino acid CDRH3 regions, and were clonally related (S9B Fig). The third human mAb, 1E12, was also derived from VH4.39, had a similar 13 amino acid CDRH3, but paired with Vλ1.44 instead. The CDRL regions for the three mAbs were 11 amino acids in length and shared some amino acid homology. Binding competition experiments using the Z1800M T/F Env gp120 protein revealed that mAb 1A8 competed strongly against the CD4bs bnAb VRC01, reducing its binding to 11%, while mAbs 2H10 and 1E12 were less effective at reducing VRC01 binding (46% and 44%, respectively) (S9C Fig). Binding of the V3 mAb 3074 and the V3 glycan bnAb PGT121 were not substantially reduced by 1A8, 2H10, or 1E12, (71 to 97% residual binding), indicating weak or no competition for these epitopes. When competed against 1A8, autologous mAbs 2H10 and 1E12 reduced binding to 16% and 40%, respectively (S9C Fig). It is therefore likely that these three neutralizing mAbs isolated from Z1800M during early infection recognize overlapping epitopes on the Z1800M T/F Env that are within or proximal to the CD4bs. We therefore used mAbs 1A8 and 1E12 to further probe the vaccine-elicited neutralization specificities.

mAb 1A8 neutralized the Z1800M T/F Env PV with an IC_50_ of 0.6 μg/ml but was unable neutralize Env D10 and D11 PV at 10 μg/ml (Fig 5F). The TF1 and TF2 chimeras were also neutralized by mAb 1A8 at IC_50_ titers like the T/F Env. In contrast, the TF3 chimeras and V5 mutants were completely resistant to neutralization by 1A8 at 10 μg/ml. Thus, the human Z1800M mAb 1A8 targets a similar V5 dependent epitope as the nAb elicited by DNA-MVA-gp120 vaccination of RM. mAb 1E12 also potently neutralized the T/F Env PV at 0.1 μg/ml but differed from 1A8 in its ability to also neutralize the 5-month Envs (Fig 5F). 1E12 neutralized Env D11 completely at 1 μg/ml but D10 was only partially neutralized at 10 μg/ml. Like 1A8, mAb 1E12 neutralized the TF1 and TF2 chimeras with similar potency as the T/F Env. 1E12 also neutralized the TF3 chimeras and the V5 mutants; however, the D10_TF3 and D10_V5 PVs were 5-fold more resistant than the T/F Env, suggesting that 1E12 neutralization is also influenced by V5 but to a lesser degree. Overall, mAb 1A8, isolated from early HIV-1 infection, recognizes a V5-dependent epitope that is similar, if not identical to, the nAb elicited by DNA/MVA/gp120 immunization in RM.

### Mass spectrometry-based glycoproteomics reveal the natures of glycans on the Z1800M T/F Env

**Fig 7:**
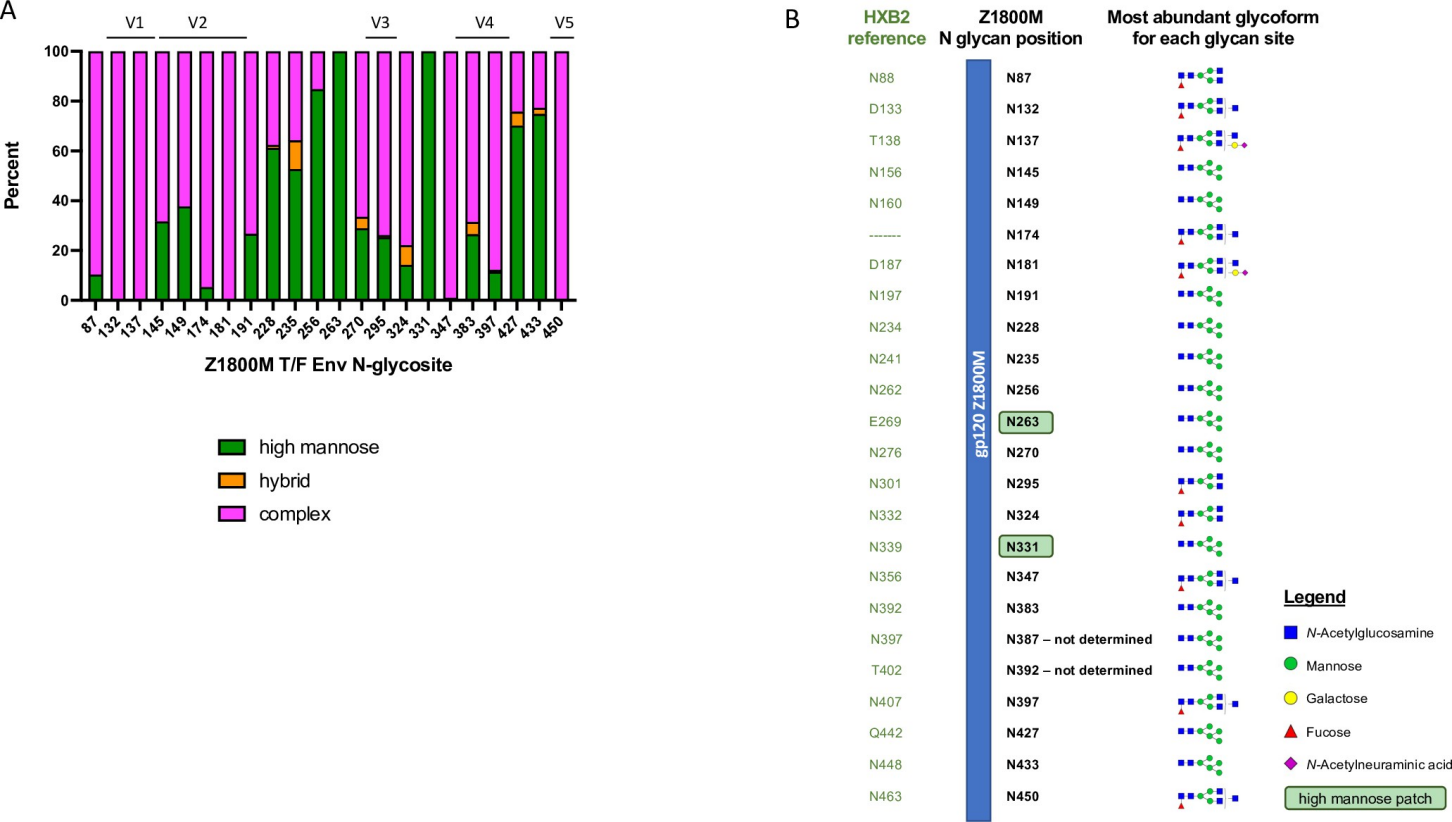
Site specific N-linked glycan profiling of the Z1800M T/F Env gp120 protein. Glycoproteomic analysis was performed using nano-LC-MS/MS and site-specific glycosylation profiling was performed for 22 of 24 putative N-linked glycan sites (N387 and N392 could not be separated and therefore not assigned with confidence). **(A)** The bar graph depicts the relative percentage of classified glycoforms on each N-glycan site (numbered according to the Z1800M T/F Env), indicated as complex (pink), hybrid (orange), or high mannose (green) types. The hyper-variable domains are indicated above the graph. **(B)** For each glycan position, the equivalent HXB2 reference position is indicated as well as the most abundant glycoform observed at that site that was subsequently used for computational modeling. For N-linked glycosylation sites N387 (HXB2 N397) and N392 (HXB N402) no glycopeptides were detected that only contained the N-glycosylation site of interest. Therefore, both sites were represented by Man5.

Vaccine elicited autologous nAb often target glycan hole regions that are exposed on both the immunogen and the native Env trimer [12,13,40]. Because the Z1800M DNA/MVA/gp120 elicited autologous nAb after the protein boosts that mapped to the V5 region, we performed mass spectrometry-based glycoproteomics to better understand how glycosylation influenced immunogenicity. There are 24 N-linked glycan motifs in the Z1800M T/F Env gp120 sequence, and glycoform profiling was accomplished at 22 of the 24 sites. For two sites located within the V4 loop, N387 (HXB2 residue 397) and N392 (HXB2 residue 402), a definitive glycoform assignment was not possible since the two sites could not be enzymatically separated. However, it was evident that both sites were significantly glycosylated. Overall, glycans on the Z1800M T/F Env gp120 were predominantly of the complex type, with subpopulations of hybrid or high mannose types (Man_5-9_GlcNAc_2_) (Fig 7A). The majority of glycosites were occupied by multiple glycoforms, with exceptions being N263 (HXB2 residue 269) and N331 (HXB2 residue 339), which were only occupied by Man_5_GlcNAc_2_ (Man5). These findings are consistent with previous studies that have demonstrated heterogeneity with a high proportion of complex type glycans on recombinant gp120 molecules [49–51].

### Computational modeling indicates exposure of the V5 loop in the Z1800M T/F Env

**Fig 8:**
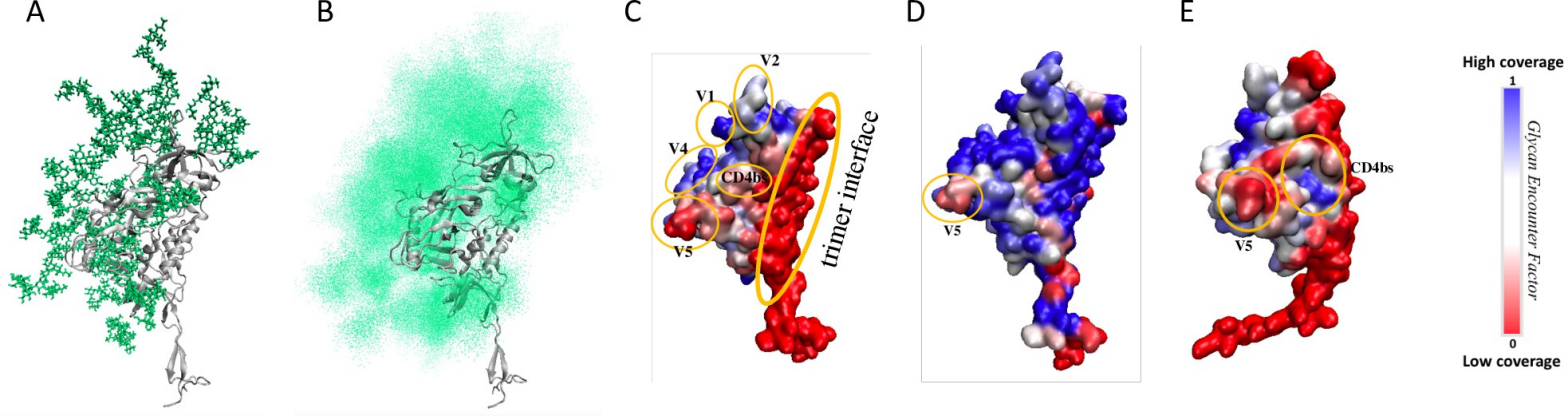
Structural model of the glycan shield topology over Z1800M gp120 glycoprotein. **(A)** A single protomer of Z1800M gp120 glycoprotein in a trimeric fold structure. Static representation of native glycans (green sticks) over Env protein (grey ribbon), with each glycan occupying only one of many possible positions. **(B)** Cumulative shielding effect due to the flexibility of glycans represented by a density of points over the monomer surface**. (C)** Z1800M gp120 surface colored by the normalized Glycan Encounter Factor (GEF) resulting from all the glycans over the monomer. Regions having high GEF or shielding are colored in blue, exposed regions are red. **(D)** Z1800M gp120 structure as part of a Man9-glycoform SOSIP trimer, colored by normalized GEF. This model includes gp120 and gp41 protein and glycans from the complete trimeric SOSIP. **(E)** Z1800M gp120 glycoprotein with monomeric conformation different from the trimer, colored by normalized GEF. This monomer was modeled using previously observed X-ray diffraction structures of HIV-1 Env monomers. The V5 loop remains exposed in all three different models.

The heterogeneity and flexibility of glycans over Env makes high resolution structural characterization of the glycan shield extremely challenging. We therefore employed computational methods to model the Z1800M Env glycoprotein and its glycan shield topology in atomistic detail as described [52,53]. The most abundant glycoform was selected for each N-glycan site, as determined by mass spectrometry (Fig 7B). Of the 22 N-linked glycans that were characterized, 12 displayed Man5 as the predominant glycoform with others as minor glycoforms. Therefore, the two glycosites that could not be assigned (N387 and N392, HXB2 N397 and NT402) were modeled with Man5. This approach resulted in a model ensemble close to the native-like glycan shield. In a static representation of the glycoprotein, each glycan takes a single conformation (Fig 8A). However, glycans are much more dynamic than the underlying protein and the overall cumulative effect resembles a cloud that provides a physical barrier over the antigen surface (Fig 8B). We quantified the shielding effect over each residue on the Z1800M T/F Env protein surface using a parameter called the Glycan Encounter Factor (GEF) [52], which provides the relative number of glycan (or protein) heavy atoms that could interfere with an approaching probe (mimicking an antibody) before it reaches the underlying protein. In other words, residues that have high GEF are shielded, while those with a low GEF are exposed. Here, we considered a protomer of a Z1800M trimer that was modeled based on the BG505 SOSIP trimer. Expressing the GEF as a colormap over the surface of the monomeric gp120 protein demonstrates that the apex and the V4 loop regions have high shielding, whereas, as expected, the region that would form the interface between monomers within the trimer is highly exposed. Interestingly, the CD4bs as well as the V5 region have lower GEF, with the entire V5 loop being highly exposed (Fig 8C). A protomer gp120 within a trimer construct may not fully represent a native gp120 monomer conformation. Due to the conformational plasticity of Env, a monomer gp120 could fold differently from a trimer construct, not necessarily having the same placement of flexible regions such as V1, V2, and V3. Therefore, we generated an independent glycosylated gp120 monomer model based on previously observed x-ray diffraction structures of HIV-1 Env monomers (Fig 8E). Further, gp120 monomers have been found to have a different glycosylation pattern with higher processing of glycans as compared to stabilized trimers [51,54]. Therefore, we generated a trimer Z1800M SOSIP model decorated with Man9 (Fig 8D). Remarkably, the exposure of V5 persisted both in the context of the independently folded gp120 monomer model and the Z1800M Env SOSIP trimer modeled with long Man9 glycans.

### Exposure of the V5 loop is dependent on the orientation of the N450 glycan

**Fig 9:**
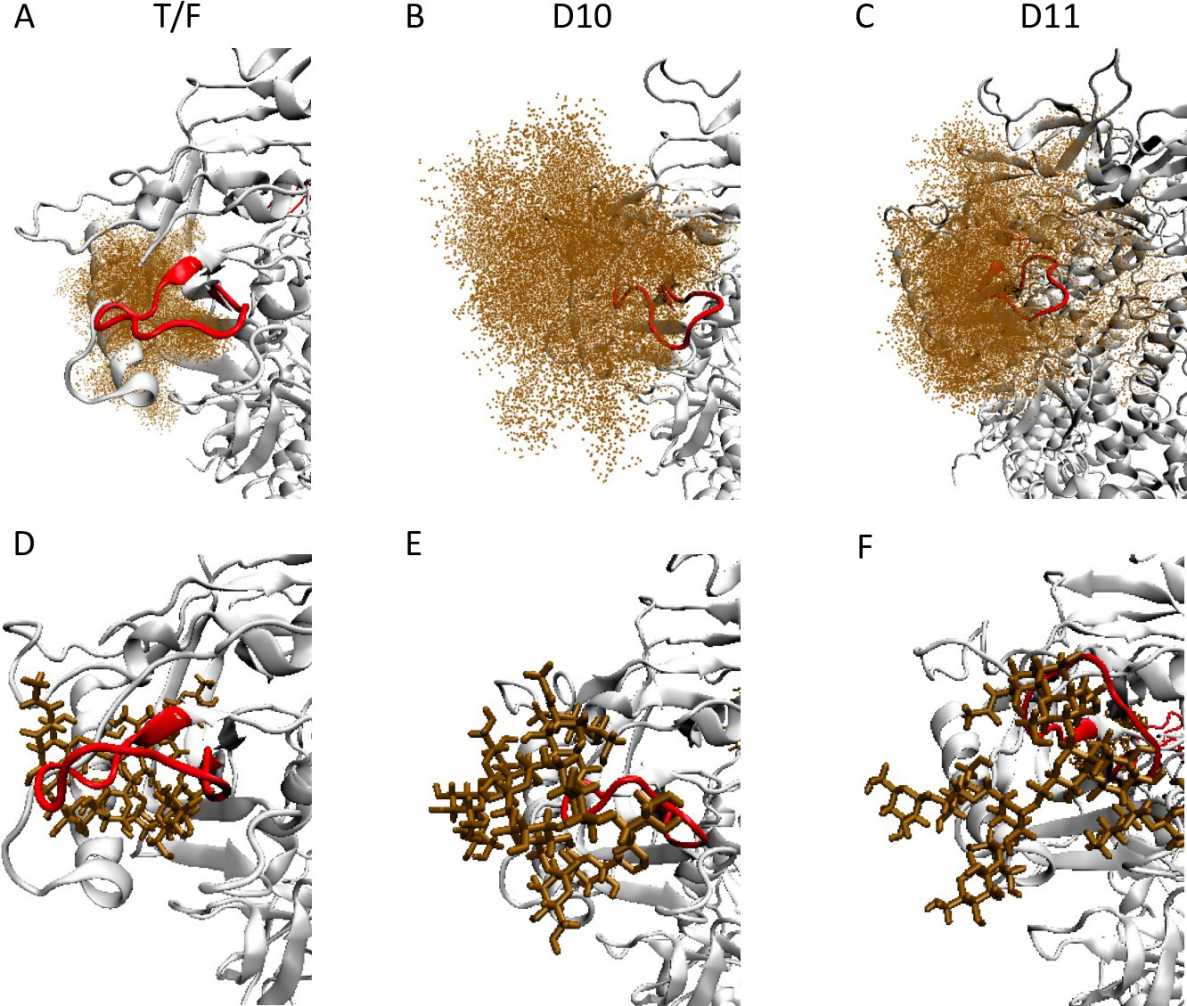
Glycan N450 reorientation leads to higher coverage of V5 in Z1800M D10 and D11. Ensemble model using the wildtype T/F, D10, and D11 Env gp120 sequences shows the glycan at N450 (HXB2 N463) on the top (brown points) and a single conformation of the glycan on the bottom (brown sticks). **(A, D)** Z1800M T/F Env where the glycan is inserted below the loop, exposing V5 and **(B, E)** D10 and **(C, F)** D11 generations where the glycan is oriented on top of the protein surface, shielding V5. The V5 hypervariable loop is shown in red.

**Fig 10:**
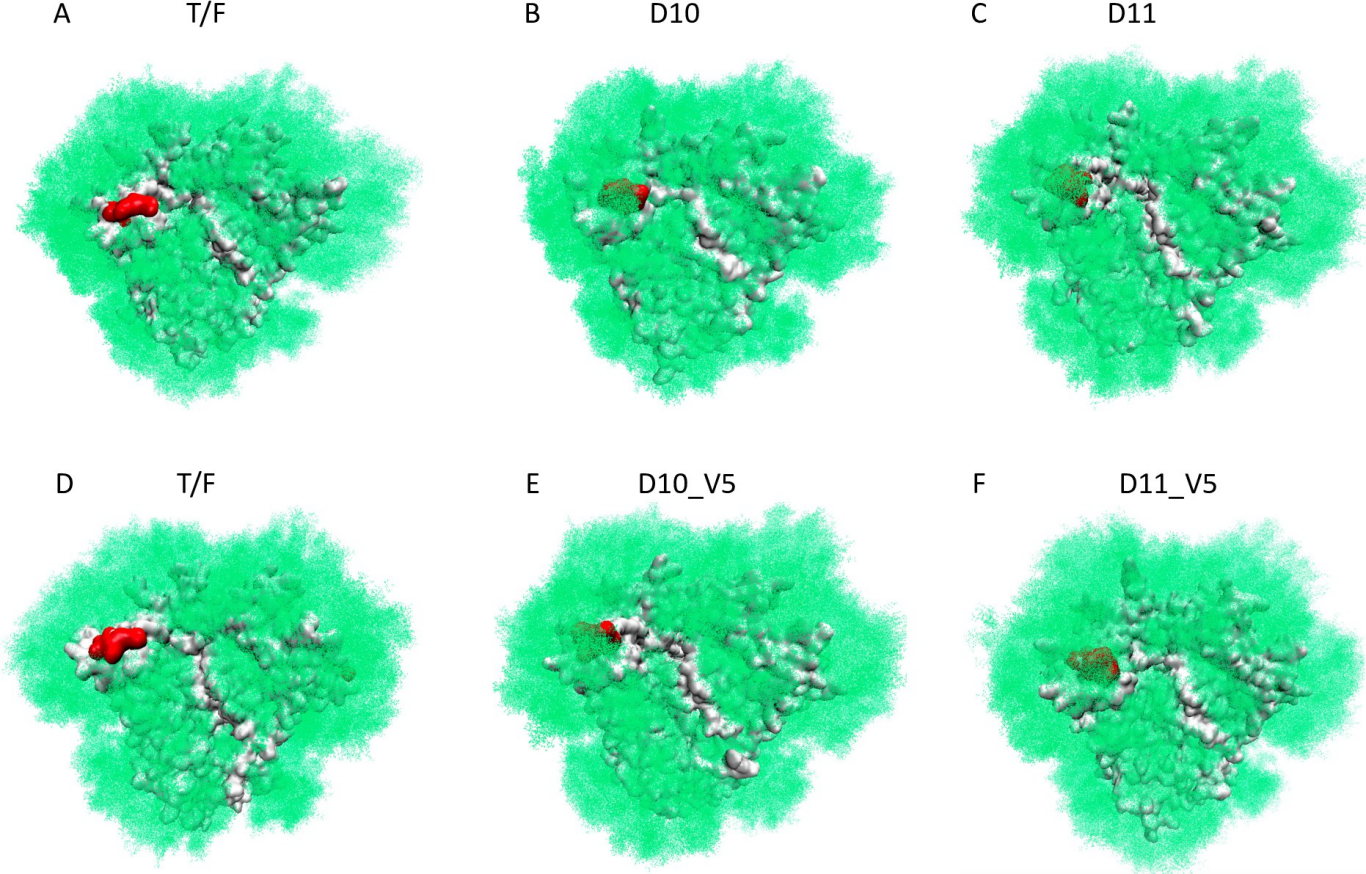
Complete glycan shield over Z1800M SOSIP structure. The glycan structural distribution is represented by green density of points. The underlying protein surface is represented in gray and the V5 loop in red. SOSIP-based models using the **(A)** T/F Env, **(B)** D10 Env and **(C)** D11 Env sequences illustrate that the V5 region remains exposed in the T/F but is shielded in the escape Envs. Because D10 and D11 contained other sequence and glycan differences outside of V5 compared to the T/F Env, the model was repeated with just the D10 and D11 V5 changes. The reorientation of the V5 glycan at N450 held true for this model suggesting that the V5 changes are sufficient: **(D)** another depiction of the T/F Env, **(E)** T/F Env with D10 V5 modifications and **(F)** T/F Env with D11 V5 loop modifications.

There are 6 N-linked glycans located proximally to the V5 loop on the T/F Env on glycosites N228, N270, N331, N347, N383, and N450 (HXB2 N234, N276, N339, N356, N392, N463). Of these, the glycan at N450 (HXB2 N463) is spatially closest to V5 but is oriented such that it is inserted below the loop. This positioning of the glycan at N450 (HXB2 N463) in relation to V5 effectively results in a hole that exposes the V5 loop (Fig 9A and 9D). As shown above, changes in V5 found in Z1800M Envs D10 and D11 caused resistance to neutralization by immunized RM serum, and plasma and a mAb from subject Z1800M. To gain further insight into mechanisms of resistance, D10 and D11 Env sequences were subjected to modeling. In Envs D10 and D11, there are several glycan and sequence differences from the T/F Env outside of the V5 loop (Fig 6A). N387 (HXB2 N397) and N174 (no analogous HXB2 glycan) are absent in D10 and D11, while D11 is also missing N397 (HXB2 N407) compared to the T/F Env. In the gp120 monomer models of D10 and D11, which contained sequence differences from the T/F Env including these glycan motifs, the V5 glycan at N450 (HXB2 N463) is projected outward, shielding the V5 loop (Fig 9B, 9C, 9E and 9F). To determine whether any sequence differences outside of the V5 loop in D10 and D11 influenced V5 loop exposure, we repeated the models with the T/F and D10/D11 with all sequence differences (Fig 10A–10C) and with just the V5 sequence changes in the T/F Env background (two substitutions in D10 and a single amino acid deletion in D11) (Fig 10D–10F). Remarkably, the local V5 changes in D10 and D11 were sufficient to drive the outward projection of the glycan at N450, indicating that the other glycans (N174, N387, N397) (HXB2 N397 and N407 with no corresponding glycan at N174) and sequence differences outside of V5 do not contribute to the evolving V5 glycan coverage.

**Fig 11:**
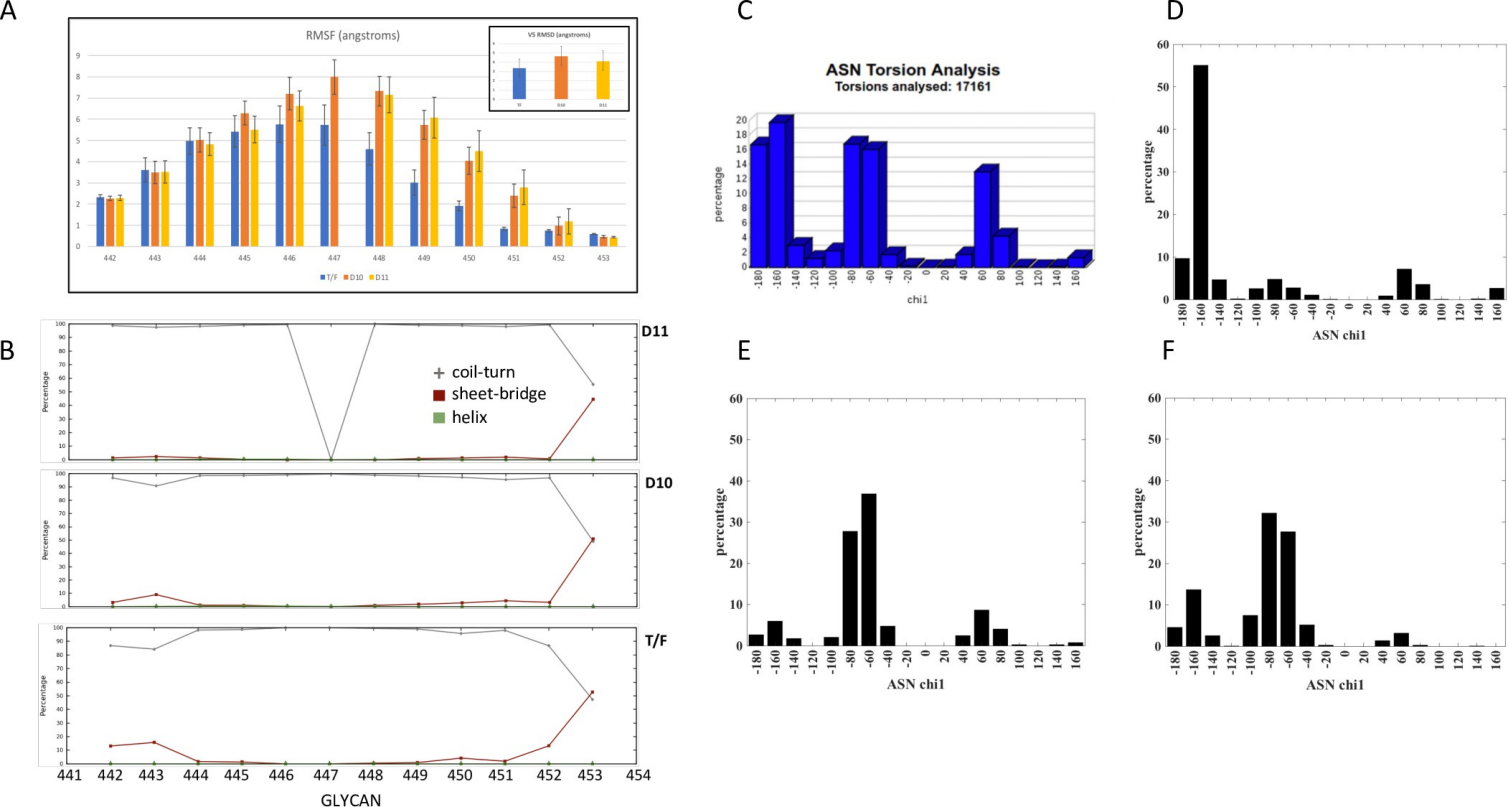
Structural variations of V5 and torsional distribution of N450 between Z1800M Envs. **(A)** Residue-wise Root Mean Squared Fluctuations (RMSF) comparison between T/F, D10 and D11, with the overall V5-loop RMS Deviation shown in inset. **(B)** Secondary structural analysis of the V5 loop indicates higher beta-strand propensity at the two terminal ends of the loop for the T/F (3^rd^ row) as compared to D10 and D11 (2^nd^ and 1^st^ rows respectively). **(C)** Chi1 distribution of glycosylated asparagines obtained from 17161 pdb structures, calculated using GlyTorsion [106]. Chi1 distribution of N450 in the T/F Env **(D)** peaks at -160°, while in D10 **(E)** and D11 **(F)** the peak is around -60°. This difference in chi1 dihedral influences the overall orientation of the glycan. The numbering in panels A and B correspond to HXB2 residues 455–465 and 456–466, respectively.

To further examine how a substitution in the protein backbone could have such a significant effect on the glycan N450 coverage of the V5 loop, we considered the V5 loop conformation as well. A detailed structural characterization of the V5 loop demonstrated that the positional deviation of the loop and the residue-wise fluctuations arising from the simulated annealing based structural relaxation of the models, are significantly higher in D10 and D11 compared to the T/F Env, especially on the C-terminal region of the loop (Fig 11A). There are minor changes to the secondary structural content of the loop upon these modifications. The residues 442, 443 and 452, 453 (HXB2 457, 458, and 465, 466) at the N- and C-terminal ends exhibit a higher percentage (occurrence of 10% or more) of beta bridge or sheet in the T/F Env but are reduced in D10 and D11 (Fig 11B). Also, the unstructured random coil length is longer in D10 and D11, leading to a differentiated distribution of torsion at N450 compared to the T/F Env. While the asparagine chi1 angles can sample three different peaks around -160°, -60° or 60°, as observed from PDB structures (Fig 11C), the T/F Env N450 (HXB2 N463) mainly peaks at -160° (Fig 11D). On the other hand, Envs D10 and D11 sample around -60° as the highest dihedral probability (Fig 11E and 11F). This 100° difference in the asparagine of N450 (HXB2 N463) results in the observed switching of the glycan from under the V5 loop to being oriented above the protein surface.

### The V5 N450 glycan affects exposure of the CD4bs

**Fig 12:**
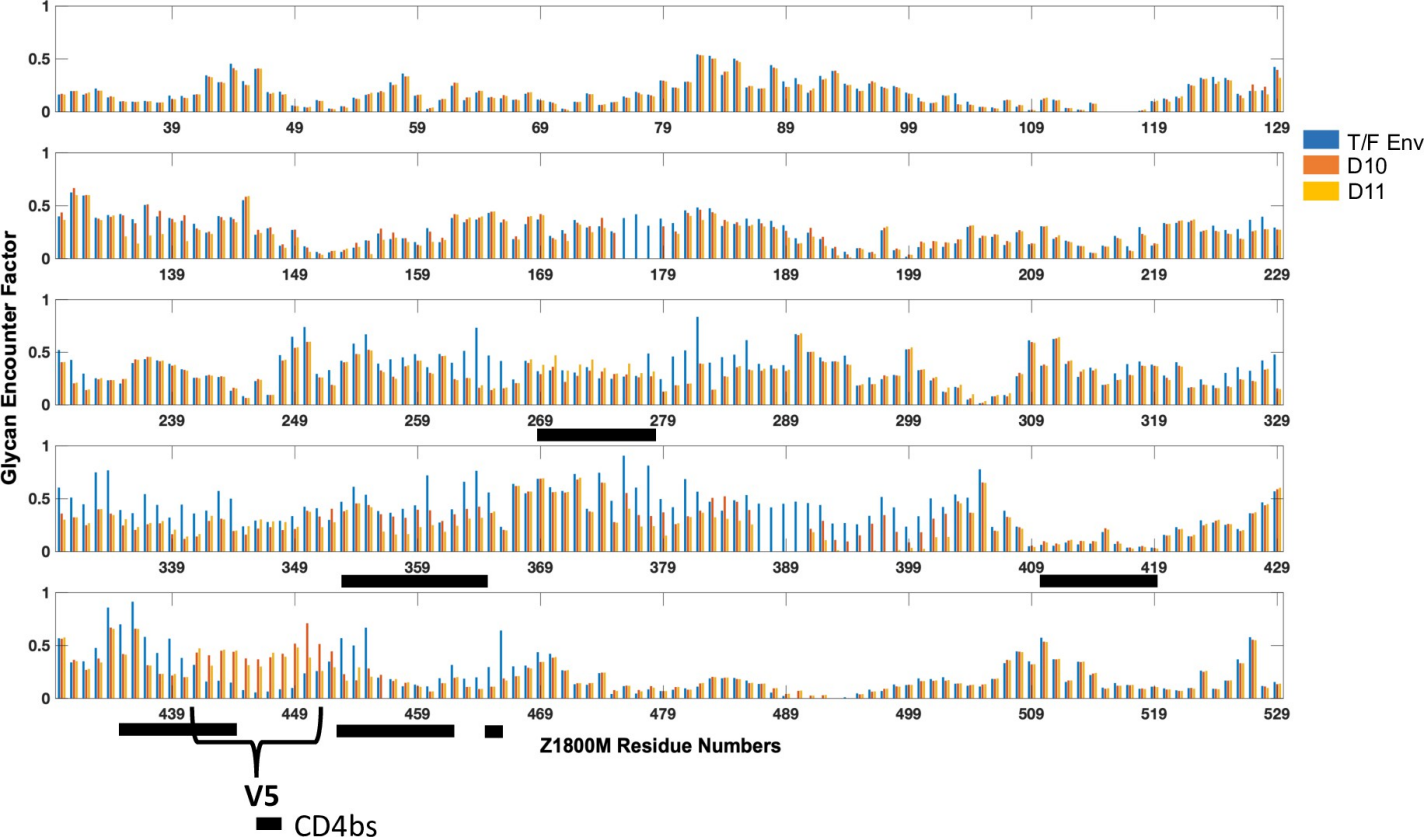
Comparison of residue-wise glycan shielding by GEF, between the T/F, D10 and D11 Envs. Amino acid positions are shown below each bar graph panel. The GEF for the T/F, D10, and D11 Envs at each position are shown on the y axis. The V5 loop region is indicated, and residues that comprise the CD4bs are marked by black horizontal bars. The bars correspond to HXB2 residues 275–286, 362–374, 425–434, 450–459, 466–475, 478–479,

The differences in glycosylation, steric as well as in number of glycosites, between the T/F Env and D10/D11 caused the glycan shielding to change over several regions on the modeled SOSIP surface (Fig 12). The primary reasons for these differences appear to be the deletion of glycans N174, N387, (and N397 in D11), (corresponding to HXB2 N397 and N407, with no corresponding glycan at N174) and the reorientation of glycan N450 (HXB2 N463). For example, changes in glycan coverage around residues 168–190, 279–289, 327–344, 376–382, and 392–403 (HXB2 179–196, 285–296, 335–352, 385–391, 402–420) are due to the N174 (no HXB2 residue) and N387 (HXB2 N397) glycan deletions. Deletion of N397 (HXB2 N407) in D11 reduces shielding in residues 134–140, 356–359, and 376–402 (HXB2 135–140, 365–368, 385–417) as compared to the T/F and D10 Envs. In contrast, the shielding of residues 249–266, 269–278, 354–384, 433–440, and 453–466 (HXB2 255–272, 268–284, 363–379, 448–455, 466–479) are driven by the change in N450 (HXB2 N463) glycan orientation. Most of the latter residues (269–278, 354–384, 433–440, 453–466) (HXB2 268–284, 363–379, 448–455, 466–479) comprise the CD4bs, suggesting that the N450 (HXB2 N463) glycan orientation affects shielding of this conserved region. As the glycan reorients from below to above the V5 loop, the coverage of the adjacent CD4bs region is reduced. A difference between D10 and D11 is that exposure of the residue range 269–278 (HXB2 268–284) increases in D10 but not in D11 (Fig 12). This is consistent with several observations. Env D10 is more sensitive to neutralization by VRC01 than the T/F while D11 is less sensitive than the T/F Env (S8B Fig). In addition, the D10 associated V5 changes introduced into the T/F Env caused increased sensitivity to VRC01, while the D11 V5 change caused a modest decrease in VRC01 neutralization, compared to the T/F Env (S8B Fig). These observations suggest that projection of the N450 (HXB2 N463) glycan with respect to the V5 loop is sufficient to significantly change exposure of the CD4bs. This was likely one of several viral escape pathways that occurred in response to autologous nAb and potentially contributed to the development of nAb breadth.

### Isolation of neutralizing mAbs from an immunized RM

**Fig 13:**
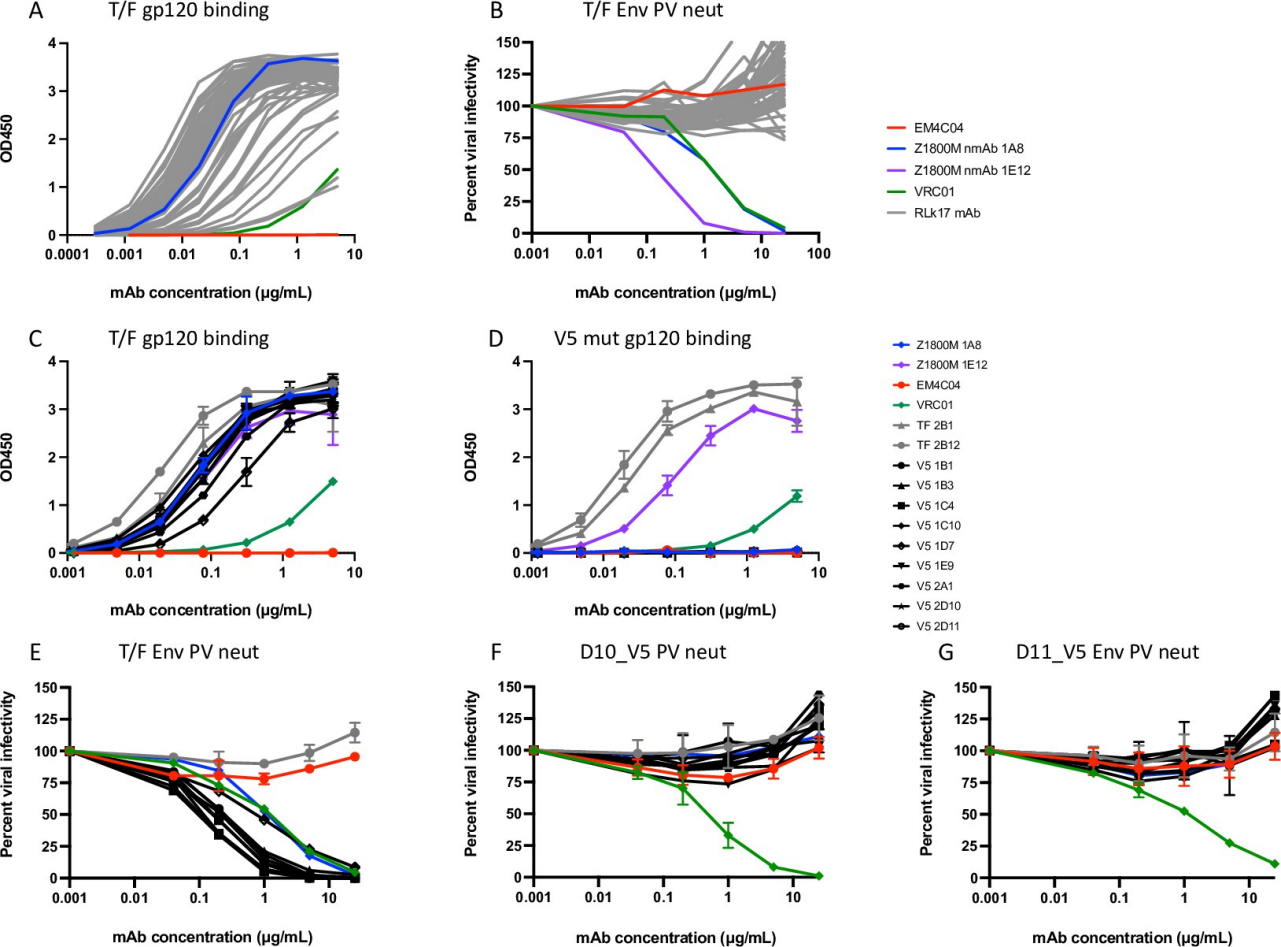
Monoclonal antibodies isolated from neutralization positive RM RLk17. 54 RLk17 mAbs recovered using the T/F Env gp120-specific B cell sort were tested in ELISA for binding to the Z1800M T/F Env gp120 protein starting at 5 μg/ml in **(A)** and for neutralization of the Z1800M T/F Env PV in the TZM-bl assay starting at 25 μg/ml in **(B)**. The bnAb VRC01; the anti-influenza HA mAb EM4C04, and the Z1800M neutralizing mAbs 1A and 1E12 are also shown. Two non-neutralizing RLk17 mAbs from the T/F Env gp120 B cell sort and nine mAbs from the expanded clonotype isolated during the T/F Env gp120 positive/V5 mutant negative B cell sort were tested for binding to the Z1800M T/F Env gp120 protein in **(C)** and to the T/F Env gp120 protein with the D11 mutated V5 loop in **(D)**. The Z1800M mAbs 1A8 and 1E12, VRC01, and EM4C04 were also included. mAbs were also tested for neutralization of the Z1800M T/F Env PV **(E)**, the D10_V5 **(F)** and D11_V5 **(G)** chimeras starting at 25 μg/ml. All assays contained duplicate wells and were repeated independently at least twice, with the means and standard deviation shown.

To gain a higher resolution view of the nAb elicited by Z1800M DNA/MVA/gp120 immunization, antigen-specific IgG+ B cells from neutralization-positive RM RLk17 were sorted into single wells from week 63 cryopreserved PBMC, which was 2 weeks post-second gp120 immunization and the peak serum nAb time point, using previously described approaches [12,15]. Using the Z1800M T/F Env gp120 protein as bait for the antigen-specific B cells, 54 paired VH and VL sequences were obtained and cloned into expression plasmids for mAb production. The mAbs were tested for binding to the T/F Env gp120 in ELISA and for neutralization of the T/F Env PV in the TZM-bl assay. All mAbs bound well to the gp120 protein, except for 2 mAbs that bound weakly like VRC01 (Fig 13A); however, none of the 54 antigen-specific mAbs had autologous neutralizing activity (Fig 13B). Notably, the human nmAbs 1A8 and 1E12, as well as the bnAb VRC01, mediated strong neutralizing activity.

**Fig 14:**
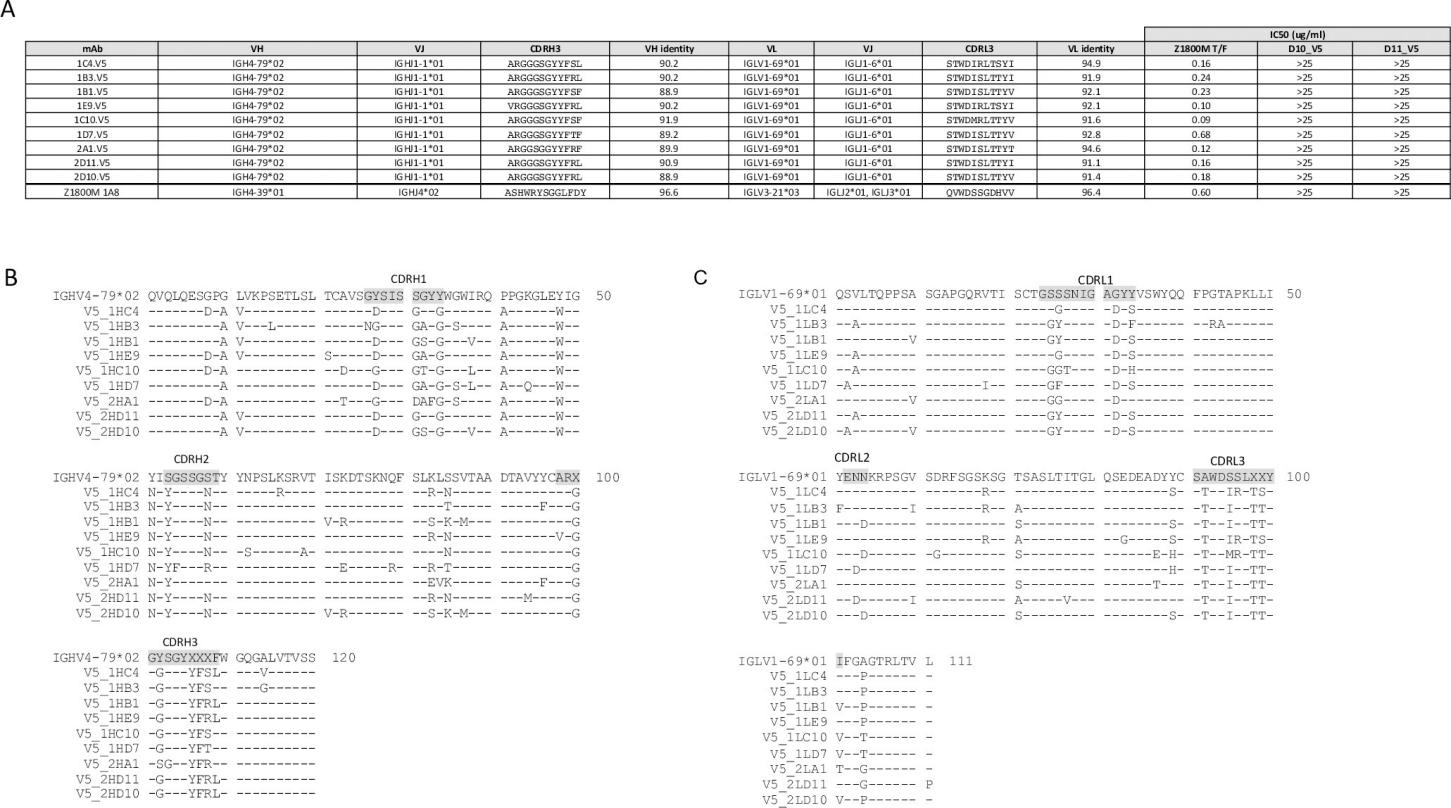
Characterization of the RLk17 neutralizing mAb clonotype. The 9 neutralizing mAbs from the expanded clonotype are shown in **(A)** along with the germline assignments for VH, VJ, VL, and VJ. The CDRH3 and CDRL3 amino acid sequences are shown, as are the percent nucleotide identity to germline for VH and VL. The last row shows the same characteristics for the Z1800M human neutralizing mAb 1A8. The last 3 columns show the IC_50_ values for each mAb against the autologous T/F Env PV (from Fig 14E–14G). A value of >25 indicates that there was no neutralization at the highest mAb concentration tested. Amino acid alignments of the VH **(B)** and VL **(C)** sequences are shown along with the corresponding germlines. Dashes represent identical residues and substitutions are shown. The CDR regions are highlighted in the germline sequences.

Since RLk17 serum nAb mapped to changes within the V5 loop, we generated a T/F Env gp120 protein with the D11 V5 loop amino acid deletion described above and tested whether the Z1800M nmAb 1A8, also dependent on V5 for neutralization, could bind to this protein in ELISA. In parallel, we tested 2 non-neutralizing mAbs isolated from RLk17. As expected, nmAb 1A8 bound to the wildtype T/F Env gp120 (Fig 13C) but not the V5-mutated gp120 (Fig 13D) whereas the non-neutralizing mAbs TF 2B1 and TF 2B12 from RLk17 bound equally well to both proteins. Unlike 1A8, the human nmAb 1E12 and bnAb VRC01 recognized both proteins, but with modest decreases in binding to the V5 mutant. We then carried out a differential B cell sort using week 63 PBMC from RLk17 and a combination of the V5-mutated and wildtype T/F gp120s as baits, based on the reasoning that B cells with neutralizing receptors would recognize the wildtype but not the V5-mutated probe (S10A and S10B Fig). Encouragingly, when we compared the VH sequences isolated using the wildtype gp120 to those from the wildype T/F+ V5 mutant- sort, a different distribution of VH germline alleles was present, suggesting enrichment of certain B cell populations (S10C Fig). The frequency of one germline, IGHV4-79*02, was more than 7-fold greater in the V5 differential sort compared to the T/F sort and this was due to a single expanded clonotype that comprised 16 of the 19 isolated sequences. Nine mAbs from this clonotype were used for further characterization (Fig 14A). As expected, these 9 mAbs bound well to the wildtype T/F Env gp120 but not the V5 mutated gp120 in ELISA (Fig 13C and 13D). Remarkably, all 9 mAbs also mediated potent neutralization of the Z1800M T/F Env PV (IC_50_ titers ranging from 0.09 μg/ml to 0.68 μg/ml) (Figs 13E and 14A) but could not neutralize when the V5 changes from D10 or D11 were present (Fig 13F and 13G, respectively). Thus, this enriched clonotype completely recapitulated the V5-dependent nature of serum nAb elicited by vaccination of RLk17 and ROa17. Alignments of the VH and VL sequences for the 9 nmAbs are shown in Fig 14B and 14C. The nmAbs had nucleotide identities to germline ranging from 88.9 to 91.9% in VH and 91.1 to 94.9% in VL (Fig 14A). The CDRH3 of this clonotype was 12 amino acids long, like the CDRH3 of the human nmAb 1A8, which is 13 amino acids long (Fig 14A). Furthermore, the RLk17 nmAb clonotype and the human nmAb 1A8 both had CDRL3 lengths of 11 amino acids. Thus, this RM-derived nmAb clonotype elicited by immunization shares key properties with an analogous V5 dependent human nmAb that arose during early HIV-1 infection against the same Env.

**Fig 15:**
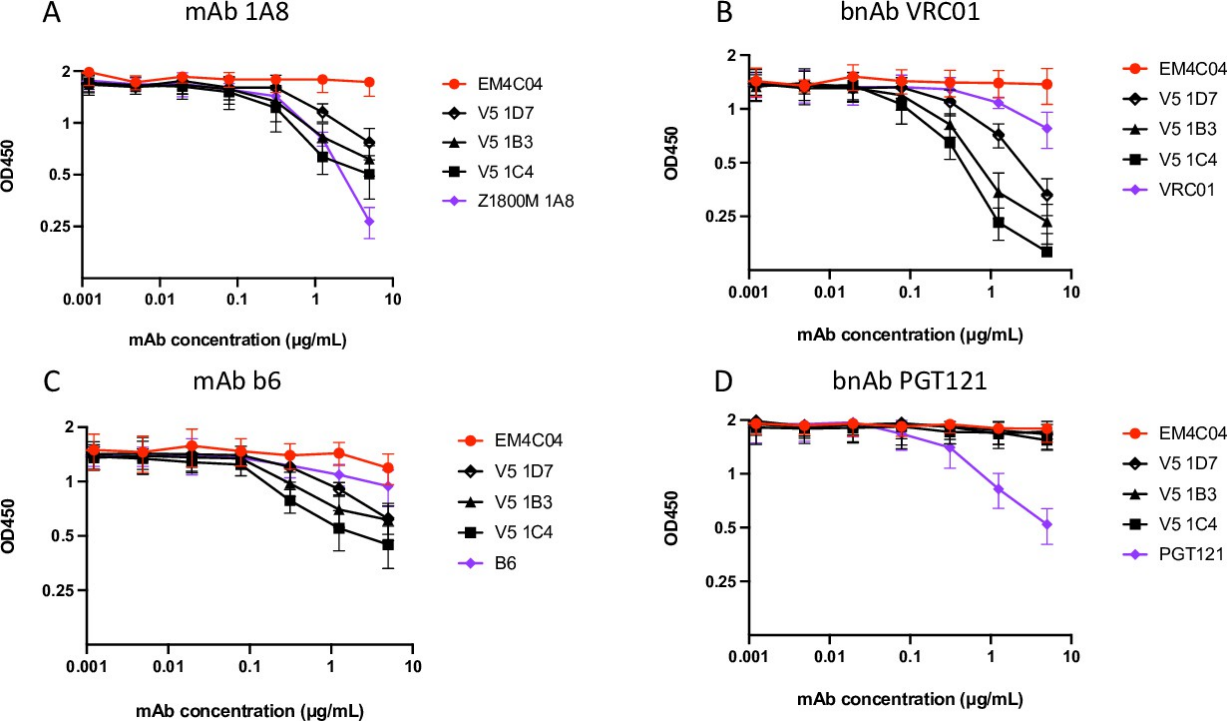
Epitope mapping through competition ELISA. The ability of representative RLk17 neutralizing mAbs V5 1D7, V5 1B3 and V5 1C4 to compete for binding of biotinylated reference antibodies Z1800M 1A8, VRC01, b6 and PGT121 is represented on each graph **(A-D)**. Immobilized Z1800M T/F gp120 protein was first incubated with serial dilutions of test antibody (starting at 5 μg/ml) and subsequently probed with biotinylated reference mAb. Concentrations of biotinylated reference mAb were used that resulted in an OD450 between 1 and 2 in absence of competitor (3 μg/ml b6 and VRC01, 0.1 μg/ml Z1800M 1A8, and 0.3 μg/ml PGT121). Each assay was independently repeated twice and the means with standard deviation are shown.

Finally, we performed a competition ELISA to further define the epitope region on the T/F Env gp120 that is recognized by the nmAb clonotype. We selected 3 representative nmAbs for these studies. First, we tested whether pre-incubation with each of the 3 clonotype nmAbs reduced binding of the human nmAb 1A8 to the T/F Env gp120 protein (Fig 15A). All 3 nmAbs tested, V5 1D7, V5 1B3, and V5 1C4, reduced binding, confirming that they recognize a similar epitope as 1A8. Because 1A8 had been shown to reduce VRC01 binding to the T/F Env gp120 (S9C Fig), we examined competition with this bnAb and a non-neutralizing CD4bs mAb b6. While VRC01 and b6 did not bind strongly to the Z1800M T/F Env gp120, and therefore did not compete well with themselves, the clonotype nmAbs were efficient at reducing their binding (Fig 15B and 15C). Consistent with these findings, the clonotype nmAbs did not reduce binding of the bnAb PGT121, which targets V3 and the high mannose patch on the outer domain (Fig 15D). EM4C04, an anti-influenza HA antibody, did not reduce binding of any of the mAbs tested. Together these results provide more evidence that the clonotype nmAbs recognize a V5 dependent epitope proximal to the CD4bs.

## Discussion

The premise underlying this study was that HIV-1 T/F Envs have inherent features that drive nAb responses during infection, and that those can be translated into vaccines to direct nAb at a certain target. In our case, we selected T/F Envs isolated from two HIV-1 infected individuals that showed distinct outcomes in terms of autologous and heterologous nAb development. We also delivered the Envs in multiple forms, as it remains unclear what the best immunogens are for eliciting autologous tier 2 nAb and for ultimately developing breadth. The findings demonstrated that the Z1800M T/F Env from the high nAb responder has an exposed, immunogenic V5 loop that was targeted by nAb in early infection and in vaccination, but only in the DNA/MVA/gp120 group. The Z1800M T/F Env DNA/MVA/trimer immunization regimen did not elicit nAb in any of the 5 RM, which could have been due to the heterogeneity of the trimer we generated. However, according to our modeling, V5 would have likely been exposed on Z1800M Env forms if it had taken on a stable trimer conformation. It could also be that the stabilizing change introduced near V5 (HXB2 A433P) altered the exposure or conformation of this region. In contrast, none of the RM immunized with R66M T/F Env vaccines (from our low nAb responder) containing gp120 or trimer developed nAb. It could be that the R66M T/F Env does not have a region that is well exposed on the native trimer, as autologous nAb titers remained low to undetectable during HIV-1 infection [4]. Indeed, there was little evidence of nAb pressure on R66M Envs during early infection, contrasting markedly with Z1800M, where there were changes in V2, V4, and V5 [4]. The only region exhibiting evidence of nAb pressure in R66M was the N-terminus of the α2 helix, which tolerates high entropy in clade C [55,56].

A unique aspect of our study was the availability of acute infection and longitudinal samples from participants in Zambia and Rwanda [57]. This enabled the use of Envs isolated from the infected subject, Z1800M, as a guide for defining vaccine elicited nAb targets. Two Envs that had escaped plasma nAb in early infection were also resistant to vaccine-elicited serum nAb. Using a combination of computational and experimental approaches, our study revealed that these viruses used a potentially novel mechanism in which the glycan shield was manipulated without removing, adding, or shifting a glycan. Essentially, an existing glycan was reoriented by changing the conformation of the V5 loop through amino acid changes outside of the sequon. The V5 loop in group M HIV-1 almost always contains 1–2 PNGS and elimination or shifting of PNGS in this region may not be tolerated because of its integration into the CD4bs [12]. However, through mutations the virus can alter the projection of the existing glycan(s). This escape mechanism employed by two early Envs during infection appears to have increased shielding of V5, while differentially influencing the accessibility of VRC01 contact sites within the CD4bs. This finding suggests that shielding of the CD4bs could be subject to strategic manipulation in this Env background. Indeed, in the clade C HIV-1 infected individual CH505, the CD4bs bnAb lineage CH103 evolved in response to escape mutations in the V5 loop of the autologous virus [67]. In that case, elongation of the V5 loop drove reorientation of CH103 bnAb to facilitate neutralization of the escape variants. In a previous study of autologous nAb responses and escape in a different clade C HIV-1 infected subject (Z185F), we described another case of V5 dependent nAb targeting at 5 months after the estimated date of infection [58]. The Z185F T/F Env V5 loop was the same length and contained a PNGS in the same position as the Z1800M T/F Env. Two amino acid substitutions in the N-terminal portion of the Z185F T/F Env V5 conferred near complete escape from contemporaneous 5-month plasma nAb and were strikingly similar to Z1800M T/F Env D10. Notably, a matching V5 peptide failed to adsorb out any plasma nAb activity against the Z185F T/F Env, leading us to posit at the time that the V5 changes affected a proximal epitope, such as the CD4bs. Taken together, these findings point to the CD4bs as a common nAb target in early clade C HIV-1 infection that is rapidly and effectively shielded by V5 dependent pathways. The possibility of exploiting these naturally occurring modifications through combined experimental and computational methods to enhance recognition of the CD4bs is a viable and enriching approach to immunogen design. In fact, phase I clinical trials based on the results of RM immunizations with broad neutralizer CH505 T/F and early Env variant gp120 immunogens are underway to recapitulate CD4bs targeting. Unfortunately, we do not have definitive information on what type or types of specificities were involved in the nAb breadth that developed in Z1800M. However, plasma from Z1800M was identified as broadly neutralizing in an independent study [16]. Using various approaches, that study ruled out targeting of N160, N332, the V1V2 apex, the glycan supersite, and the gp120/gp41 interface, but the results from gp120 and resurfaced core adsoprtion experiments were inconclusive. Therefore, isolation of mAbs from Z1800M at nAb breadth associated time points is worth pursuing.

A striking feature of our study is that we were able to eliminate all serum nAb activity by one or two changes in V5 that were derived from *in vivo* escape. This contrasts with serum nAb mapping studies of BG505 SOSIP immunized RM, where closing glycan holes results in only partial resistance [12]. This could indicate that the Z1800M DNA/MVA/gp120 vaccine elicited a single nAb specificity, whereas the BG505 SOSIP is known to elicit a polyclonal nAb response against multiple glycan holes [13,14]. The predictability of eliciting nAb that target a single region could be an advantage for driving them down a path towards breadth, with the caveat that there were also many other non-neutralizing antibody specificities elicited. The T/F Env appears to play an important role in determining the course of nAb during infection; however, it is impossible to know which of the thousands of longitudinal Envs are needed to drive breadth. In our study, a V5 dependent escape pathway in two different Env variants had distinct effects of VRC01 neutralization and exposure of the CD4bs. Thus, if the initial nAb target is well defined on an Env immunogen, then computational approaches could facilitate sequential modifications to shift nAb while avoiding other changes selected *in vivo* that could be counterproductive. A powerful way to use the Z1800M T/F Env could be to design immunogens that successively increase coverage of V5 and exposure of the CD4bs. Such re-direction of nAb could be achieved by altering the structural flexibility of the V5 loop or restricting the N450 (HXB2 N463) glycan in an orientation away from CD4bs. Moreover, CD4bs glycan coverage is relatively lower in Z1800M than other common Envs such as BG505 SOSIP (S11 Fig), potentially making it a better vehicle to elicit CD4bs-targeting antibodies.

To provide a more detailed understanding of vaccine-elicited nAb in comparison to the HIV-1 infected subject, mAbs were isolated from the immunized RM that developed the highest ID_50_ titer. From these, a single expanded clonotype with neutralizing activity against the T/F Env completely recapitulated the serum nAb. The clonotype was present at a very low frequency until we enriched for B cells whose receptors could bind to the T/F Env gp120 but did not bind when V5 was mutated. The nmAbs were derived from a commonly used VH4 RM germline and were able to achieve neutralization with modest levels of somatic hypermutation in the VH and VL. The nmAbs were also remarkably similar to the human nmAb 1A8, which was even less mutated from its assigned germline than the RM mAbs. In addition to their identical dependency on V5, the VH of the human nmAb 1A8 shared 77% amino acid identity with a representative RLk17 clonotype nmAb, V5_1C4, with CDRH3 lengths differing by only 1 residue. This suggests strikingly convergent immune evolution in VH in response to the T/F Env. The VLs were less conserved, sharing 61% amino acid identity between these two mAbs, but the CDRL3 regions were the same length. In Z1800M, there were also other nAb specificities that developed during early infection besides that represented by 1A8. For example, the nmAb 1E12 competed modestly with 1A8 but was only partially sensitive to the V5 changes, reflecting ongoing evolution of the immune response against viral variants.

Our study provides evidence that the nAb outcomes we observed were imprinted on the Z1800M and R66M T/F Envs to some extent, a concept that has been supported by other studies in RM and in HIV transmission pairs [6,59–61]. Our approach also highlights the complexity of generating soluble HIV trimers in the context of diverse variants. While R66M formed more stable trimers than Z1800M, both trimer populations exposed non-neutralizing epitopes in V3, CD4bs, and gp41. They also likely generated non-neutralizing antibodies directed against the trimer base, although we did not measure this directly. Z1800M also has some unique features that could have contributed to its lack of soluble trimer stability. For one, the V3 loop is 32 amino acids instead of the more usual 33 for clade C Env. Also, five glycans are thought to contribute to BG505 trimer stability, located at HXB2 residues N156 and N160 in the V1V2 apex, N262, N295, and N386 [53,62]. While R66M displayed all five N-linked glycosylation motifs, Z1800M lacked HXB2 residues N295 and N386. Furthermore, stabilization methods other than the UFO might improve trimerization. It is also conceivable that the small group sizes and/or administering only two protein boosts limited development of nAb in immunized RM. Nevertheless, the results illustrate variation in stabilizing genetically diverse HIV-1 Envs as well as highlighting gaps between *in vitro* stabilization vs the native Env trimer.

It is noteworthy that a gp120 Env immunogen elicited tier 2, autologous nAb in nonhuman primates. Examples of gp120 containing regimens that did not elicit autologous nAb in human subjects include the Vaxgen 003 and 004 trials that delivered recombinant gp120 proteins, and the RV144 and HVTN702 trials that administered gp120 following canarypox viral vector [63]. However, the Envs in those trials were not selected for their propensity to elicit tier 2 nAb. Here, nAb was not detectable until after the first protein immunization, suggesting that the gp120 either initiated this response *de novo* or boosted one or more pre-existing nAb-capable lineages initiated by DNA/MVA. It will be important to determine whether DNA/MVA played a role in priming the nAb clonotype we isolated, or if there are others. In addition, there are several approaches that can potentially be used to increase the frequency of immunized RM that develop autologous nAb and boost the nAb titers, including additional immunizations, different delivery methods, and a more powerful adjuvant than what was done in this study. Others have also shown that an escalating dose approach is more effective than a bolus in eliciting higher nAb titers and potentially enhancing breadth [64]. Finally, we could attempt to stabilize the Z1800M T/F Env trimer by adding targeted glycans, increasing the V3 loop length, and performing selection to enhance for well-formed trimers. However, our results do suggest that Z1800M T/F Env gp120-based regimens can reproducibly elicit V5 targeted nAb. Indeed, other studies including our own have described V5 loop targeting on other HIV-1 strains by autologous nAb following vaccination, HIV-1 infection, and SHIV infection of RM, including in settings involving the CD4bs and development of breadth [6,8,12,13,58,61,65–70]. The predictability of nAb targeting against this Env, in the vicinity of the CD4bs, the eventual development of heterologous neutralization breadth, and the availability of longitudinal samples and sequences, makes this T/F Env and its longitudinal variants a strong candidate for further immunogen design.

## Methods

### Ethics statement

This study was approved by the Institutional Animal Care and Use Committee (IACUC) at Emory University and complied with NIH guidelines. Animal research was also in compliance with the Animal Welfare Act and other Federal statutes and regulations relating to experiments involving animals. All animal research adhered to the principles stated in the 2011 Guide for the Care and Use of Laboratory Animals prepared by the National Research Council. Yerkes National Primate Research Center (YNPRC) is fully accredited by the Association for Assessment and Accreditation of Laboratory Animal Care (AAALAC). Methods of euthanasia were consistent with the American Veterinary Medical Association with Guidelines.

This study also utilized samples obtained previously from individuals enrolled in heterosexual discordant couple cohorts in Rwanda and Zambia [4,71,72]. After obtaining written informed consent, blood samples were collected from HIV-1 infected participants longitudinally. All couples in the cohorts were provided monthly counseling and testing prior to the HIV-negative partner becoming positive. The procedures for written informed consent and research activities were approved by institutional review boards at all collaborating clinical research centers, with further compliance to human experimentation guidelines set forth by the United States Department of Health and Human Services. The parent cohort study was reviewed and approved by the Republic of Rwanda National Ethics Committee, the Emory University Institutional Review Board, and the University of Zambia Research Ethics Committee. To protect confidentiality, all subject identification numbers were anonymized by assigning a coded ID that removes any identifying information.

### Animals and immunizations

20 Indian origin rhesus macaques (*Macaca mulatta*), including 17 male and 3 female, were included in this study. All animals were between 3 and 5 years of age at the start of the study. Immunizations were as follows: week 0 (lt. and rt. outer thigh) and week 8 (lt. and rt. outer thigh) with pGA1-SHIV-R66M/Z1800M DNA (1.5 mg DNA in 0.4 ml administered to each site; total 3 mg each time point); week 16 (lt. and rt. outer thigh) and week 24 (lt. and rt. deltoid) with rMVA-SHIV-R66M/Z1800M (5 x 10^7^ PFU in 0.5 ml administered to each site; total 1 x 10^8^ PFU each time point); weeks 53 (lt. and rt. deltoid) and 61 (lt. and rt. deltoid) with either gp120 or trimer protein (50 μg in 0.5 ml administered to each site; total 100 μg each time point). All immunizations were performed intramuscularly with each immunization dose split equally between left and right anatomic sites. The RM were split into 2 groups of 10 animals each and one group received reagents derived from the HIV T/F Env Z1800M E4.02 sequence while the other received reagents derived from the HIVT/F Env R66M O20.03 sequence [4]. For the protein immunizations, the groups of 10 were further subdivided with 5 animals receiving gp120 protein and the other 5 animals receiving the stabilized gp140 trimer. All protein immunizations were adjuvanted with Adjuplex (Empirion LLC) following the manufacturer’s suggested protocol. One animal in the Z1800M DNA/MVA/gp120 vaccine group was removed from the study before the protein immunizations due to clinical health issues that were unrelated to the immunization protocol.

### Rhesus macaque sample collection/processing

For serum collection, whole blood was collected into SST tubes and spun at 4°C for 30 min at 2000rpm in a tabletop centrifuge. Separated serum was heat inactivated at 56°C for 45 min, aliquoted and stored at -80°C. For PBMC isolation, whole blood was collected into CPT tubes (BD vacutainer 362761), processed according to the manufacturer’s recommendations and purified PBMC were cryopreserved in 90% FBS/10% DMSO in liquid nitrogen vapor phase storage.

### Construction of DNA plasmid vaccine vectors expressing the T/F Envs

The DNA vaccines were constructed in a plasmid vector backbone of pGA1 consisting of a eukaryotic expression cassette with a CMV immediate early promoter (CMV-IE) and intron A that facilitates gene splicing, as described previously [41,42,73,74] (S4A Fig). The DNA constructs express SIVmac239 derived Gag/Pro/RT and HIV-1 Tat, Rev and Env derived from the cloned T/F Env cassette. Safety mutations were incorporated into *gag* to inactivate RNA packaging and into *pol* corresponding to protease and reverse transcriptase to inactivate their functions. All genes were expressed from a single promoter using the sub-genomic splicing that is used by HIV/SIV. Using a pGA1 DNA construct containing the HIV-1 C.1086 K160N Env as a base plasmid, the gp160 coding region was replaced with the corresponding open reading frames from R66M and Z1800M T/F Envs. The plasmid pGA1-SHIV-R66M was synthesized by GenScript and pGA1-SHIV-Z1800M was synthesized by Aldevron. The expression of Gag and the cognate Env was confirmed by flow cytometry (S4B Fig). Nucleotide sequences were confirmed.

### Generation of recombinant MVA vaccines expressing the T/F Envs

A wildtype MVA (isolated before 1974) was used to generate the recombinant MVA (rMVA) vaccines, as described previously [41,42,74]. The SIVmac239 Gag/Pro/RT coding region was inserted into the Deletion III region of MVA and the HIV-1 Env gp150 coding region (with a cytoplasmic truncation at residue 744 for R66M or 728 for Z1800M, 741 in HXB2) from either R66M or Z1800M was inserted in between two essential MVA genes, I8R and G1L. R66M and Z1800M Env DNAs were optimized for vaccinia codon usage, synthesized (GenScript), cloned into the pLW-73 vector (which contains GFP) and sequence verified. To make MVA recombinants, DF-1 cells were infected with MVA expressing Gag/Pro/RT and co-transfected with the pLW-73 plasmid vector. After 48 hours, lysates were prepared and used to infect DF-1 cells. Thirty-six hours later, these cells were stained using the anti-HIV-1 Env V3 glycan bnAb PGT121 to detect Env expression on the surface and to sort for Env+ GFP+ double positive cells. Sorted cells were lysed, the lysate was used to infect DF-1 cells, and GFP+ plaques were isolated and stained to identify recombinants using anti-SIV Gag mAb 2F12 (intracellularly) and anti-Env bnAb PGT121 (on the surface). After 7 rounds of plaque purification, seed stocks were developed from a final recombinant that expresses Gag and Env but not GFP. Vaccine stocks were developed using seed stocks, purified on sucrose cushions, characterized by flow cytometry and western blot, sequence verified, titered, and used for immunization. As can be seen in S4C Fig, both Z1800M and R66M rMVAs express Gag and Env intracellularly and on the cell surface. Western blot analysis confirmed Gag and Env in the lysate and in supernatants of the infected cells suggesting virus-like particle formation (S4D Fig).

### Production of T/F Env protein immunogens

To produce the gp120 proteins, the nucleotide region 6225 to 7757 encoding amino acid residues 1 to 511 (HXB2 numbering) followed by a stop codon was synthesized for each T/F Env by GeneArt (Life Technologies, ThermoFisher Scientific) using gene optimization and then subcloned into the pcDNA3.4 expression vector. Plasmids were transiently transfected into Expi293F cells using the ExpiFectamine 293 Transfection Kit (Thermo Fisher Scientific, A14525) according to the manufacturer’s instructions. The transfected culture supernatant was collected at days 5–7, clarified via low-speed centrifugation, and passed through a 0.22 μm Steritop vacuum filtration unit (Millipore, S2GPT02RE). The clarified, filtered supernatant was passed through *Galanthus nivalis* lectin coupled agarose beads (Vector Laboratories, AL1243) reconstituted in an Econo-Column chromatography column (Bio-Rad, 7372512) for purification of the protein. After overnight drip at 4°C, the column was washed with 1X PBS, and protein was eluted in 7–10 volumes of 1 M α-methyl mannopyrannoside and fractions were pooled. Purified protein was concentrated, and buffer exchanged into 1X PBS, 500 mM NaCl using an Amicon Ultra-15 centrifugal filter unit (Millipore, UFC903024). The protein was further purified using Size Exclusion Chromatography using a HiLoad Superdex S200PG column (GE Life Sciences, 28989335) on an AKTA Pure HPLC system (GE Life Sciences). Peak fractions corresponding to monomeric gp120 were further visualized on Blue Native gel electrophoresis. The gp120 containing fractions were pooled and the final yield of each purified gp120 protein was ~1.5mg. Protein was stored in aliquots of 50 μl at a concentration of 2–3 μg/μl at -80°C.

To produce trimeric gp140 proteins, the nucleotide region 6225–8216 encoding amino acid residues 1 to 664 (HXB2 numbering) followed by two stop codons was synthesized for each T/F Env by GeneArt (Life Technologies, ThermoFisher Scientific) using gene optimization and then subcloned into the pcDNA3.4 expression vector, with the following modifications: residues 1–29 were replaced with the CD5 leader sequence (MPMGSLQPLATLYLLGMLVASVLA) and residues 508–511 representing the furin cleavage site were replaced with a linker (GGGGSGGGGS). In addition, each construct contained a series of amino acid substitutions that had been described previously to stabilize other HIV-1 Env gp140 proteins. The R66M NFL contained V65K, A73C, Q106T, I201C, N302Y, E429R, R432Q, A433C, K500R, F519R, I520R, Q543N, I559P, T569G, K588R, A561C [18–21]. The Z1800M UFO contained H66R, H170Q, T316W, A433P, A501C, L535N, T588E, D589V, T605C, E662A [18,27,30,44], and residues 547–568 were replaced with NPDWLPDM [29]. DNA plasmids were transiently transfected into Expi293F cells using the ExpiFectamine 293 Transfection Kit (ThermoFisher, A14525) according to manufacturer’s instructions. Large cultures of 400 to 600 ml were maintained at 8% CO_2_ and 80% humidity in a shaking platform incubator (Infors HT Multitron) at 120 rpm in baffled, vented flasks (VWR). After 5 days, supernatant was collected, with all subsequent procedures performed at 4°C. The supernatant was centrifuged at 3,500 rpm for 45 min, followed by filtration through a 0.22 μm Steritop vacuum filter (Millipore, S2GPT02RE). The clarified, filtered supernatant was passed through *Galanthus nivalis* lectin coupled agarose beads (Vector Laboratories, AL1243) reconstituted in an Econo-Column chromatography column (Bio-Rad, 7372512) for purification of the protein. After overnight drip at 4°C, the column was washed with 1X PBS, and protein was eluted in 7–10 volumes of 1 M α-methyl mannopyrannoside and fractions were pooled. Purified protein was concentrated, and buffer exchanged into 1X PBS, 500 mM NaCl using an Amicon Ultra-15 centrifugal filter unit (Millipore, UFC903024). SEC was performed using a 16/600 Superdex 200 Column (GE Life Sciences, 28-9893-35) on an AKTA Pure HPLC system (GE Life Sciences). The BG505 NFL trimer DNA plasmid, kindly provided by Dr. Richard Wyatt, was used to express the BG505 NFL trimer as a procedural control. Trimeric protein containing fractions were collected, pooled, and concentrated in 1X PBS, 150mM NaCl. Trimer protein was aliquoted and stored at -80°C.

### Binding antibody multiplex analysis (BAMA)

BAMA was used to measure serum IgG binding to 8 gp120, 8 gp140, and 16 V1V2 scaffold antigens as described [17]. The immunogen proteins Z1800M gp120, Z1800M trimer, R66M gp120, R66M trimer, were also included. All Env antigens were produced in 293F cells. Preimmunization serum samples (week -4) and those collected at weeks 2, 10, 18, 26, 55, and 63 from 20 immunized RM were analyzed (one did not have samples at weeks 55 and 63). Six 5-fold serial dilutions of serum starting at 1:80 were analyzed for IgG binding to the antigen panel and the mean fluorescence intensity at each dilution was then used to generate an area under the curve (AUC) value for each sample. Bound IgG was detected using biotinylated anti-macaque IgG. The criteria for positivity were as follows: at dilution 1:80, MFI > 100, MFI > antigen-specific cutoff established using the 95^th^ percentile of all baseline sample binding levels per antigen, MFI > 3-fold of that of the matched baseline before and after Blank/MuLVgp70_His6 subtraction. BAMA was also used to measure HIVIG and monoclonal antibody binding to the immunogen proteins Z1800M gp120, Z1800M trimer, R66M gp120, R66M trimer. The immunogen proteins were directly coupled to polystyrene beads and binding of antibodies was measured using mouse anti-human IgG-biotin followed by streptavidin-PE. Each antibody-protein combination was tested in duplicate. PGT145 and PGT151 were tested starting at a concentration of 20 μg/ml followed by ten 4-fold serial dilutions. All other mAbs were tested at a single concentration of 20 μg/ml. HIVIG was tested starting at a 1:100 dilution followed by ten 6-fold serial dilutions (S3 Fig). Normal human serum was used as a negative control.

### Multi-parameter statistical analysis of BAMA serum IgG binding profiles

An analysis was conducted using a two-level linear mixed effects model (LMM) with the function lmer() under the package lme4 1.1.26 in R 4.0.3. Time points and subject ID (antiID) were considered to have random effects, for which a random intercept as well as a random slope were added to the model. Four dummy variables were created and corresponded to four vaccines, respectively. Another dummy variable was created to indicate the type of protein (gp120 or trimer). The antigen gp70-TT31P.2F10.2792 V1V2 was excluded since data was missing and subject 6S0 was also excluded since the visits were not complete. The effects of the four vaccines were evaluated by LMM with time points (visits), specific vaccine and their interactions as the covariates. Additionally, the effects of the two proteins were compared with visits, proteins, and their interactions as the covariates. In the LMM model, the protein “gp120” served as the reference level.

### Isolation of mAbs from human subject Z1800M and immunized rhesus macaque RLk17

mAbs were recovered from single, antigen-specific B cells using procedures described previously for both HIV-1 infected subject Z1800M and immunized RM RLk17 [12,15]. The variable domain sequences are available in Genbank (accession numbers MK269911-270058 and OM829895—OM830020). The Z1800M T/F Env gp120 protein was produced with a C-terminal 6XHis-tag was produced by GeneArt (ThermoFisher). To produce the protein, the coding sequence was synthesized and inserted into pcDNA3.3, transfected into FreeStyle 293 cells, and purified from the cell supernatant via 5 ml HisTrap Fast Flow column, linear gradient from 20–500 mM Imidazole in PBS, 500 mM NaCl at 6 days post-transfection, with a HiLoad 26/600 Superdex 200 polishing step. Methods for the isolation of IgG variable domains from Z1800M antigen-specific B cells and production of mAbs has been described [15]. For isolation of mAb from RM RLk17, week 63 cryopreserved PBMC (∼10 million cells per sort) were washed and resuspended in PBS + 5% FBS. After counting, cells were incubated with Z1800M T/F gp120-His and stained with live/dead fixable viability dye eFluor 780 (65-0865-14, eBioscience,), anti-CD14 PE-Cy7 (367111, BioLegend), anti-CD3 Pacific Blue (317313, BioLegend), anti-CD20 BV650 (302335, BioLegend), anti-IgG FITC (555786, BD Pharmingen). Anti-His PE (130-120-718, Miltenyi Biotec) and anti-His APC (130-119-782, Miltenyi Biotec) were used to detect RM B cells that recognized the gp120 probe. Sorts were carried out using a FACS Aria Cell Sorter with the following gating strategy: size, singlets, live, CD14-, CD3-, CD20+, IgG+, gp120/His+. Live CD20+ IgG+ B cells that were double positive for Z1800M T/F gp120 (PE and APC) were single cell sorted into 96-well plates containing 10 μl cell lysis buffer (Superscript III RT buffer, Tween, DTT, 18080–044, Invitrogen) and RNaseOUT (1077019, Invitrogen). To select for B cells from RLk17 that were dependent on V5, a Z1800M Env gp120 protein containing the D11 V5 deletion at E447 (HXB2 460) was produced by GENEART (ThermoFisher). This protein was labeled with the Alexa Fluor 647 Microscale Protein Labeling kit (ThermoFisher) according to the manufacturer’s instructions. Staining and sorting were performed as above with selection for cells positive for Z1800M T/F gp120-His (labelled with anti-His PE) binding and negative for binding to Z1800M D11 V5-AF-647. Plates were temporarily placed on dry ice before moving to -80°C for storage.

Immunoglobulin heavy and light chain variable domain regions were PCR-amplified as previously described, using oligo-dT (ThermoScientific, AM5730G) and SuperScriptIII (Invitrogen, 18080–044) [12,15,75]. For RLk17, full-length cDNA amplification from single cells was performed using a modified version of the SMART-Seq II protocol [76], as previously described [77]. One μl of the RT reaction was used to amplify heavy (IgG only), kappa, and lambda chain variable regions using high performance liquid chromatography (HPLC) purified primers as described in [78] using Phusion Hotstart II High Fidelity DNA Polymerase (F537S, Thermo Fisher Scientific) for first round PCR. For the second round PCR reaction, 2.5 μl of the first round was used as a template in addition to external primers used in Liao et al. [75]. PCR variable region amplicons were combined with a CMV promoter containing DNA fragment, and the appropriate corresponding constant region DNA fragment (including a poly A tail) via overlapping PCR [75]. Plasmids HV0024, HV0023, HV0025 and HV0026 were kindly provided by Dr. Larry (Huaxin) Liao from the Duke Human Vaccine Institute. VH and VL plasmid pairs were co-transfected at a 1:2 ratio into 6-well plates containing Expi293F cells (Thermo Fisher Scientific, Expi293 Expression System Kit, A14635). Five to seven days post-transfection, mAbs were purified from the cell culture supernatant using Ab SpinTrap with Protein G Sepharose High Performance (GE Healthcare, 28408347). The concentrations of the purified mAbs were quantified on an Octet RED96 using Anti-Human IgG Fc AHQ biosensors (ForteBio, 18–5001). RM Ig germlines were assigned to VH and VL sequences using IgBLAST version 1.16.0 and a custom database [64]. Analysis of somatic hypermutation was performed using Change-O and SHazaM packages from the Immcantation pipeline (Version 4.1.0) [79].

### Assessment of antigenicity and epitope binning (competition) using biolayer interferometry (BLI)

Binding was assessed on an Octet RED96 (ForteBio, Inc, Menlo Park, CA) at 30°C, with 1,000 rpm agitation. Each mAb was immobilized on an Anti-hIgG Fc Capture (AHC) biosensor (Fortebio, 18–5060) at a concentration of 25 or 10 μg/ml for 300 s, and a baseline reading was recorded for 60 s in kinetics buffer (PBS with 0.01% BSA, 0.02% Tween20, and 0.005% sodium azide). Sensors were then immersed in varying molarities of the corresponding autologous T/F gp120 protein (0.1875 to 1.5 μM) or trimer (0.037 to 1 μM) for 300 s, then allowed to dissociate in kinetics buffer for 600 s. Sensors were regenerated before each kinetics assay. Human IgG at 25 or 10 μg/ml dipped into kinetics buffer was included on every assay plate for reference during analysis (SouthernBiotech, 0150–01). Data was analyzed using ForteBio Data Analysis 9.0. Binning (competition) assays were performed at 30°C, with 1,000 rpm agitation. Synthesized Z1800M T/F gp120 at a concentration of 25 μg/ml was immobilized on an Anti-Penta-HIS (HIS1K, ForteBio, 18–5120) biosensor for 300 s, and a baseline reading was recorded for 30 s in kinetics buffer. Sensors were then immersed in 0.66 μM primary mAb for 10 minutes. After a second baseline reading for 30 s in kinetics buffer, sensors were immersed in 0.66 μM secondary antibody to assess reference mAb binding capacity in the presence of primary mAb binding (‘reference mAb’- VRC01, 3074, PGT121). Reference mAb as primary and secondary mAb was included as a positive control for competition (reduced reference binding). Binning values were calculated using ForteBio Data Analysis 9.0.

### Reference monoclonal antibodies

The VRC01 mAb VH and VL expression plasmids were obtained through the NIH AIDS Reagent Program, Division of AIDS, NIAID, NIH: from Dr. John Mascola (12035 and 12036) [80,81]; mAb 3074 was kindly provided by Xiangpeng Kong, NYU School of Medicine; mAb PGT121 was obtained through the NIH AIDS Reagent Program, Division of AIDS, NIAID, NIH: Anti-HIV-1 gp120 monoclonal (12343) [82]. Plasmids expressing the VH and VL for PGT121, b6, F105, PG16, PG9, VRC06b, 447-52D, PGDM1400, 17B, PGT145, PGT128 were kindly provided by Dr. Richard Wyatt at the Scripps Research Institute. Anti-influenza HA antibody EM4C04 was kindly provided by Dr. Jens Wrammert at Emory University.

### Neutralization assay

Neutralization was measured using serially diluted, heat-inactivated immunized RM serum, RM or human plasma, or mAbs in the TZM-bl assay as previously described, using cells plated one day prior to the assay [4,15,58,83–94]. In brief, Env PV was generated by transfecting the Env-expressing plasmid DNA alongside the HIV-1 SG3ΔEnv proviral backbone DNA into 293T cells, using the Fugene HD reagent as recommended (Promega). PV stocks were collected from the 293T cell supernatants at 48–72 hours after transfection, clarified by centrifugation, divided into small volumes, and frozen at –80°C. 2,000 IU of each titered Env PV (or virus) (in DMEM with ∼3.5% (vol/vol) FBS (Hyclone) and 40 μg/ml DEAE-dextran) was mixed with five-fold serial dilutions of heat-inactivated serum or plasma samples, or mAb, and assayed for inhibition using the TZM-bl indicator cell line, with luciferase as the readout. At 48 h post-infection, the cells were lysed and luciferase activity was measured using a BioTek Cytation3 multimode microplate reader. The average background luminescence from a series of uninfected wells was subtracted from each experimental well. Experimental wells were compared against virus without a test reagent (100% infectivity). All assays contained duplicate wells and were repeated at least once independently. Neutralization ID_50_ or IC_50_ titer values were calculated in Graphpad Prism using the dose–response inhibition analysis function with variable slope, log-transformed x values, and normalized y values.

### ELISA

To measure Z1800M or R66M-specific IgG antibodies in RM serum, 96-well plates (Nunc MaxiSorp, flat bottom, 44-2404-21) were coated at 4°C overnight with 1ng/μl Z1800M or R66M gp120 (produced as described above) in carbonate-bicarbonate buffer (1.5g Na_2_CO_3_ + 2.9g NaHCO_3_ in 1L H_2_O). The plates were washed 3x with PBS-T (0.05% Tween in PBS) and incubated for 2 hours in blocking buffer (3% BSA in PBS-T diluted from Blocker BSA 10% in PBS stock, Thermo Fisher Scientific, 37525) followed by an additional 3 washes in PBS-T prior to addition of samples. Heat inactivated RM serum samples were diluted in blocking buffer to starting concentrations between 1:100 and 1:1000. This initial dilution was then serially diluted 3-fold in blocking buffer and 100 μl was added to each well (8 concentrations tested for each sample) and incubated for 2 hours at room temperature. After 6 washes in PBS-T, HRP-conjugated anti-monkey IgG (Alpha Diagnostic Intl., 70021) was added to each well at a 1:10,000 dilution in blocking buffer and incubated for 1 hour at room temperature. The plates were washed 6 times in PBS-T, and then tetramethylbenzidine SureBlue 1-component microwell peroxidase substrate (KPL, 52-00-03) was added. The plate was incubated in the dark at room temperature for 15 min, at which time 4 N H_2_SO_4_ stop solution (Fisher Scientific, SA818-1) was added. The optical density at 450 nm was determined on an Epoch Microplate Spectrophotometer (BioTek), and the data were analyzed using Gen5 software. Quantitation of gp120-specific serum IgG was performed by including a standard curve for IgG on each plate. For this, 2 columns of each plate were treated as above except for coating with goat anti-rhesus IgG unlabeled (Southern Biotech, 6200–01) and incubating with serial dilutions of Rhesus IgG (Southern Biotech 0135–01; 7 dilutions in blocking buffer starting at 100ng/ml). Concentrations of serum IgG that bound to gp120 were determined using all dilutions that fell within the quantitative range of the standard curve and were represented by the average for each sample. Each assay was run with duplicate wells and one independent repeat.

To measure mAb binding to Z1800M Env protein, 96-well plates (Nunc MaxiSorp, flat bottom, 44-2404-21) were coated at 4°C overnight with 1 ng/μl Z1800M-T/F-His or Z1800M-V5-D11-His gp120 in carbonate-bicarbonate buffer (1.5g Na_2_CO_3_ + 2.9g NaHCO_3_ in 1L H_2_O). The plates were washed 3x with PBS-T (0.05% Tween in PBS) and incubated for 2 hours with blocking buffer (3% BSA in PBS-T diluted from Blocker BSA 10% in PBS stock, Thermo Fisher Scientific, 37525) followed by an additional 3 washes in PBS-T prior to addition of samples. An initial 5 μg/ml mAb were serially diluted 4-fold in blocking buffer and 100 μl was added to each well (8 concentrations tested for each sample) and incubated for 2 hours at room temperature. After 6 washes in PBS-T, HRP-conjugated anti-human IgG (Southern Biotech, 2040–05) was added to each well at a 1:10,000 dilution in blocking buffer and incubated for 1 hour at room temperature. The plates were washed 6 times in PBS-T, and then tetramethylbenzidine SureBlue 1-component microwell peroxidase substrate (KPL, 52-00-03) was added. The plate was incubated in the dark at room temperature for 10 min, at which time 4 N H_2_SO_4_ stop solution (Fisher Scientific, SA818-1) was added. The optical density at 450 nm was determined on an Epoch Microplate Spectrophotometer (BioTek), and the data were analyzed using Gen5 software. Each assay was run with duplicate wells and one independent repeat. For Z1800M T/F gp120 competition ELISAs, mAb b6, VRC01, Z1800M 1A8 and PGT121 were biotinylated using the EZ-Link-Sulfo-NHS-LC-Biotinylation Kit (Thermo Fisher Scientific) and ELISA assays were performed as above with minimal modification. Specifically, His-tagged Z1800M T/F gp120 protein was pre-bound to Qiagen Ni-NTA HiSorb 96 well plates (Qiagen 35061) for two hours. Following the 2 hour mAb incubation step performed as above (8 4-fold serial dilutions starting at 5ug/ml) the plates were washed and incubated with biotinylated mAb for an additional 2 hours. This was followed by an additional 6 washes with PBS-T before the addition of 100 μl/well of a 1:200 dilution of Pierce High Sensitivity Streptavidin-HRP (Thermo Fisher Scientific 21134) for 1 hour.

### Purification and quantitation of RM serum IgG

IgG was purified from 100 μl RM serum samples using protein G Ab Spin Trap columns (Cytiva LifeSciences) according to the manufacturer’s suggested procedure with a final elution volume of 200 μl. The amount of IgG present in the initial serum sample as well as in the flow through and elution fractions were quantified by ELISA. 96-well plates (Nunc MaxiSorp, flat bottom 44-2404-21) were coated at 4°C overnight with 1 ng/μl Goat anti-rhesus IgG UNLB (Southern Biotech 6200–01) in carbonate-bicarbonate buffer. The plates were washed 3x with PBS-T (0.05% Tween in PBS) and blocked for 2 hours with blocking buffer (3% BSA in PBS-T diluted from Blocker BSA 10% in PBS stock, Thermo Fisher Scientific 37525) followed by an additional 3 washes in PBS-T prior to addition of samples. Heat inactivated RM serum samples, flow through and elution fractions from the column were diluted in blocking buffer to starting concentrations of 1:500. This initial dilution was then serially diluted 4-fold in blocking buffer and 100 μl added to the wells (8 concentrations tested for each sample) and incubated for 2 hours at room temperature. After 6 washes in PBS-T, HRP-conjugated anti-monkey IgG (Alpha Diagnostic Intl 70021) was added to each well at a 1:10,000 dilution in blocking buffer and incubated for 1 hour at room temperature. The plates were washed 6 times in PBS-T, and then tetramethylbenzidine SureBlue 1-component microwell peroxidase substrate (KPL 52-00-03) was added. The plate was incubated in the dark at room temperature for 15 min, at which time 4 N H_2_SO_4_ stop solution (Fisher Scientific SA818-1) was added. The optical density at 450 nm was determined on an Epoch Microplate Spectrophotometer (BioTek), and the data were analyzed using Gen5 software. Quantitation of gp120-specific serum IgG was performed by including a standard curve for IgG on each plate. For this, 2 columns of each plate were incubated with serial dilutions of Rhesus IgG (Southern Biotech 0135–01; 7 dilutions in blocking buffer starting at 100 ng/ml). Concentrations of IgG present were determined using all dilutions that fell within the range of the standard curve and were represented by the average for each sample. Each assay was run with duplicate wells.

### Plasmids and cloning

Cloning of HIV-1 Env expression plasmids R66M_07MAR06_O20, Z1800M_26JUN07_E4, Z1800M_30NOV07_D10 and Z1800M_30NOV07_D11 was described previously [4]. These plasmids include the full-length patient derived Env coding region as derived from single genome PCR amplification as well as upstream and downstream flanking sequences. Chimeras were generated between the T/F Z1800M_26JUN07_E4 (Z1800M T/F Env) sequence and the escape variants Z1800M_30NOV07_D10 (Z1800M D10) and Z1800M_30NOV07_D11 (Z1800M D11). A section of Z1800M D10 and Z1800M D11 between the vector BamHI restriction site and the BsrGI restriction site located at Env residue C296 (HXB2 numbering) (a fragment including upstream SGA sequences, signal peptide, V1 and V2) was used to replace the corresponding segment in the T/F Env to generate Z1800M_TF1_D10 and Z1800M_TF1_D11 chimeras respectively by standard digestion and T4 DNA ligase mediated cloning. A second set of chimeras, Z1800M_TF2_D10 and Z1800M_TF2_D11 was generated in a similar manner by replacing the region between restriction sites BsrGI (HXB2 Env residue C296) and PmlI (HXB2 Env residue R456) (a fragment including V3, CD4bs, V4) in the T/F Env with the sequences from Z1800M_D10 and Z1800M_D11. To generate the third set of chimeras, Z1800M_TF3_D10 and Z1800M_TF3_D11 where the C-terminal part of the T/F Env (from V5 onward) was replaced with the corresponding sequence from Z1800M_D10 and Z1800M_D11, forward primers Z1800M TF3 D10 Pml1 F (5’ GAA TAA TAT TAA CAC GTG ATG GAG GAA CTA CAG GGG AGT CA 3’) and Z1800M TF3 D11 Pml1 F (5’ GAA TAA TAT TAA CAC GTG ATG GAG GAA TTA CAG AGT CAA AT 3’) and reverse primer Z1800M TF3 D10/D11 Xho1 R (5’- GCC CTC TAG ACT CGA GCG GCC GCC ACT GTG CTG -3’) were used to amplify the region between restriction sites PmlI (Env residue 456) and XhoI (located in the vector following the end of the SGA fragment) followed by InFusion (Takara) insertion of this fragment into the T/F Env backbone cut with these same enzymes. Finally, to generate Z1800M_V5_D10 and Z1800M_V5_D11, fragments containing the T/F Env nucleotide sequence from PmlI to XhoI with the V5 changes found in Z1800M_D10 and Z1800M_D11 were synthesized (IDT) and inserted via InFusion into the T/F Env plasmid. All final constructs were verified by nucleotide sequencing. Heterologous Env expression constructs were obtained from the NIH HIV Reagent Program, Division of AIDS, NIAID, NIH: Human Immunodeficiency Virus 1: 93MW965.26 gp160 Expression Vector (pSVIII-93MW965.26), ARP-3094, contributed by Dr. Beatrice Hahn and the Panel of Global Human Immunodeficiency Virus 1 (HIV-1) Env Clones, ARP-12670, from Dr. David Montefiori.

### Site-specific glycan profiling of Z1800M gp120 via mass spectrometry

*Glycoproteomic sample preparation*, *nano-LC-MS/MS data acquisition and data analysis of monomeric Env gp120 Z1800M*: Sequencing-grade modified trypsin and chymotrypsin were purchased from Promega (Madison, WI). Sequencing-grade modified endoproteinase Glu-C was acquired from Roche Diagnostics (Indianapolis, IN). All other reagents were purchased from Sigma Aldrich unless indicated otherwise. Data analysis was performed using Byonic 2.3 software and manually using Xcalibur 4.2 and GlycoWorkbench 1.1. *Protease digestion for glycoproteomics of gp120 Z1800M*: The purified and lyophilized monomeric T/F Env gp120 protein Z1800M was dissolved in 50 mM ammonium bicarbonate solution. The protein was reduced in 12 mM dithiothreitol solution, and subsequently alkylated in 30 mM iodoacetamide. In total, three enzymatic digestions were set up for best coverage: (i) tryptic digest for 16 h at 37°C, (ii) tryptic digest (16 hours at 37°C) followed by a chymotryptic digest at room temperature for 16 hours, (iii) tryptic digest (16 hours at 37°C) followed by a digest with endoproteinase Glu-C at 37°C for 16 hours in phosphate buffer. The three digests were individually filtered through 0.2 μm filter and directly analyzed by LC-MS/MS. *Data acquisition of protein digests using nano-LC-MS/MS*: The glycoprotein digests were analyzed on an Orbitrap Fusion Tribrid mass spectrometer equipped with a nanospray ion source and connected to a Dionex Ultimate 3000 RSLC nano system (Thermo Fisher Scientific, Waltham, MA). A pre-packed nano-LC column (Cat. No. 164568, Thermo Fisher Scientific, Waltham, MA) of 15 cm length with 75 μm internal diameter (id), filled with 3 μm C18 material (reverse phase) was used for the chromatographic separation of the samples. The precursor ion scan was acquired at 120,000 resolutions in the Orbitrap analyzer and precursors at a time frame of 3 s were selected for subsequent MS/MS fragmentation in the Orbitrap analyzer at 15,000 resolution. The LC-MS/MS runs of each digest were conducted for 72 min. 0.1% formic acid and 80% acetonitrile-0.1% formic acid was used as mobile phase A and B, respectively, to separate the glycopeptides. The intensity threshold for triggering an MS/MS event was set to 2000 counts, and monoisotopic precursor selection was enabled. MS/MS fragmentation was conducted with a stepped HCD (Higher-energy Collisional Dissociation) product triggered CID (Collision-Induced Dissociation) (HCDpdCID) program. Charge state screening was enabled, and precursors with unknown charge state or a charge state of +1 were excluded (positive ion mode). Dynamic exclusion was enabled for an exclusion duration of 30 s after 2x detection within 20 s. *Data analysis of glycoproteins*: The LC-MS/MS spectra of all three enzymatic digests of the monomeric gp120 Z1800M mutant were searched against the FASTA sequence using the Byonic software 2.3 by choosing appropriate peptide cleavage sites (non-specific cleavage option enabled). Carbamidomethylation of cysteine was set as a fixed modification, whereby oxidation of methionine and common human N-glycans found in plasma (variable) were set as variable modifications. The LC-MS/MS spectra were also analyzed manually for the glycopeptides with the support of the Thermo Fisher Scientific Xcalibur 4.2 software, GlycoWorkbench 1.1., GlycoMod tool, and ProteinProspector v6.2.1. The HCDpdCID MS^2^ spectra of glycopeptides were evaluated for the glycan neutral loss pattern, oxonium ions and glycopeptide fragmentations to assign the sequence and the presence of glycans in the glycopeptides. Spectra for the most common glycoform at each site used for computer modelling are included in S1 Appendix that also includes the glycoproteomics workflow, which was created with the help of BioRender.com.

### Computational modeling of Z1800M glycoprotein structures

Glycoprotein conformational ensemble was generated by a previously established high throughput atomistic modeling pipeline (HTAM)[52,53]. In this method, robust ensembles of fully glycosylated Z1800M monomer and trimer structures are modeled based on the CHARMM36 forcefield[95]. Ten distinct underlying protein scaffolds were homology modeled from different experimentally determined SOSIP structures deposited in the Protein Data Bank, by assimilating the variations in local structural regions including loops, that arise due to flexibility. 100 template-free glycan conformations were generated for each of the 10 scaffolds, having random initial orientations. The resulting collection of 1000 glycoprotein conformations were relaxed by conjugate gradient energy minimization followed by simulated annealing, with template-based protein backbone restraints and glycan topological restraints to enforce proper stereochemistry. This integrated technique can sufficiently sample a physiologically relevant conformational space, as has been extensively validated with cryo-electron microscopy experiments [53]. The most probable glycan-type was selected for each N-glycan site, as determined by the site-specific mass spectrometry results. Protein templates used for trimers and trimeric sfold of gp120 are as listed in Table S2 of reference [52]. The non-trimeric gp120 structure was built by a combination of different intact segments of monomeric structures. The core of the monomer was modeled based on PDB ID 3JWD; the V1-V2 hypervariable loop region was built based on PDB structure 3U4E, and the template for the V3 hairpin structure was a JR-FL gp120 structure with an intact V3 loop (PDB ID 2B4C). Shielding effect over the protein surface was calculated per-residue, based on the glycan encounter factor (GEF) score [52]. This was calculated as the geometric mean of the probability that a probe approaching a surface residue would encounter glycan heavy atoms, in perpendicular and tangential directions. Probe size of 6 angstrom diameter was chosen to mimic a typical hairpin loop. All homology modeling was performed using MODELLER [96] and ALLOSMOD libraries [97]. Computational modeling calculations were implemented with VMD 1.9 [98], Python [99] and MATLAB 2018 [100]. All structural graphics were generated using VMD 1.9.

### Negative-stain electron microscopy and image processing

EM analysis of R66M, BG505, and Z1800M Env trimers were performed as described before [101]. Briefly, 4 μl of aliquot containing Env trimers (0.001–0.003 mg/mL) was applied for 1 min onto a freshly glow-discharged, 300-mesh carbon-film Cu mesh grid (EMS-CF300, Hatfield, PA, USA), and then negatively stained with 0.75% uranyl formate for 1 min. Data were collected using an FEI Tecnai T12 electron microscope operating at 120 kV, with a low electron dose of below 40 e^-^/Å^2^, and a magnification resulting in a pixel size of 1.011 Å for BG505 and 1.648 Å for R66M and Z1800M. Images were acquired on a Gatan OneView CMOS camera with a nominal defocus range of 0.5 to 1 μm using the SerialEM program [102]. Particles were picked automatically using Relion 3.1 [103,104]. Extracted picked particles (non-CTF corrected) were imported into EMAN 2.9 and an initial per-particle CTF correction followed by a reference-free 2D classification using binned (2X) particles via MSA/MRA (e2refine2d.py) was performed [105]. Particles corresponding to trimers, not aggregates or fragments, were selected into a subset for another round of 2D reference-free alignment. In the end, 20 classes were generated.

## Supporting information

S1 Fig Antigenicity of Z1800M and R66M T/F Env gp120 and trimer immunogens. The Z1800M and R66M T/F Env gp120 proteins **(A)**, the Z1800M UFO trimer and R66M NFL trimer **(B)**, and the BG505 NFL trimer as a process control **(C)** were analyzed by measuring the binding of HIV bnAbs, non-neutralizing mAbs, and HIVIG through BAMA or BLI. For the BLI experiments, which were conducted on an Octet Red96, each antibody was immobilized on a biosensor at a concentration of 10 μg/ml **(A)** or 25 μg/ml **(B)** and the sensors were immersed in varying molarities of each protein. The gp120 proteins were tested at concentrations ranging from 0.1875 to 1.5 μM and the trimers were tested at concentrations ranging from 0.037 to 1 μM. The individual sensorgrams are shown in S2 Fig. For the BAMA experiments, the untagged immunogen proteins were directly coupled to polystyrene beads and antibody binding was measured using mouse anti-human IgG-biotin primary followed by a streptavidin-PE secondary detection reagent, which was quantified by fluorescence intensity. The BAMA fluorescence units are shown in S3 Fig. In panels **A-C**, the antibody, binding specificity, and detection method is shown for each antibody-protein combination. The color intensity and number of plus signs indicate the relative strength of binding; gray boxes with a dash indicate that binding was below detection; NT indicates ‘not tested’. Some antibody-protein combinations (e.g. F105, VRC01, 17b) were analyzed using both methods and the results of both are shown.
(TIF)

S2 Fig Antigenic characterization of the protein immunogens using biolayer interferometry. **(A)** The R66M and Z1800M gp120 immunogen proteins were analyzed by BLI, using an Octet Red96, using HIV bnAbs PG16, VRC01, PGT121, as well as non-neutralizing CD4 binding site mAb F105, and autologous non-neutralizing R66M mAb7A2 and autologous neutralizing Z1800M mAb 1A8 as controls. Each antibody was immobilized on a biosensor at a concentration of 10 μg/ml and the sensors were immersed in varying molarities of each protein. The gp120 proteins were tested at concentrations ranging from 0.1875 to 1.5 μM. **(B)** The R66M NFL trimer and Z1800M UFO trimer were also analyzed by BLI, using V3-glycan bnAbs PGT121, PGT128; V1V2 apex bnAbs PGT145, PGDM1400, PG9, PG16; CD4 binding site bnAb VRC06b and non-neutralizing mAb F105; CD4-induced non-neutralizing mAb 17b; and V3 bnAb 447-52D. The trimers were tested at concentrations ranging from 0.037 to 1 μM with antibody immobilized at 25 μg/ml. Response units are indicated on the y axis and are plotted against time on the x axis. Related to Fig 1.
(TIF)

S3 Fig Antigenic characterization of the protein immunogens by BAMA. The fluorescence intensity data generated via antibody binding to the immunogen proteins in the BAMA assay is shown. Proteins were analyzed using V1V2 apex bnAb PGT145, gp120-gp41 interface bnAb PGT151, both with serial dilutions starting at 20 μg/ml; a panel of bnAb and non-neutralizing antibodies PGT125, 19B, 17B, VRC01, CH102, F105, 7B2, and an anti-influenza bnAb CH65 at 20 μg/ml; HIVIG at serial dilutions starting at 1:100; and normal human serum (NHS-60) diluted at 1:500 as a negative control. Related to Fig 1.
(TIF)

S4 Fig DNA and MVA vaccine constructs. **(A)** A schematic representation of the recombinant DNA plasmid that expresses SIVmac239 Gag/Pro/Pol and HIV-1 Env is shown. **(B)** Flow cytometric detection of HIV-1 Env (bnAb PGT121) and SIV Gag (mAb 2F12) expression in R66M and Z1800M T/F Env recombinant DNA plasmid transfected 293T cells. **(C)** Flow cytometric detection of SIVmac239 Gag and HIV-1 Env in DF-1 cells infected with the R66M and Z1800M T/F Env recombinant MVA vectors. Surface Env was stained using PGT121 and intracellular Env was detected using mAb ID6. Surface and intracellular Gag was detected using mAb 2F12. Gag and Env expression were plotted on live cells infected with rMVA, stained intracellularly for MVA E3 protein (mAb TW2.3, BEI Resources). **(D)** Lysates (L) and supernatants (S) from rMVA infected DF-1 cells were probed with serum from a BG505 SOSIP-immunized RM to detect HIV-1 Env on western blot and SIV Gag was detected using mAb 2F12.
(TIF)

S5 Fig Serum IgG binding to Env protein panel by BAMA. Serum IgG binding to 8 gp120 proteins, 8 gp140 proteins, and 16 scaffolded V1V2 antigens from the BAMA antigen panel, as well as the R66M gp120 and trimer and Z1800M gp120 and trimer, was analyzed with respect to time (weeks). The data was colored according to the type of protein in **(A)** and the subtype of the Env proteins in **(B)**. The top row contains Z1800M T/F Env immunized RMs that received the trimer or gp120 immunogen, as indicated, and each line represents the mean response of RM in that group against one gp120/gp140/V1V2 binding antigen. The bottom row contains the same information for R66M T/F Env immunized RMs. The mean MFI (mean fluorescence intensity) was calculated for RMs in the four vaccination groups and is plotted on the y-axis on a log10 scale for each binding antigen. The time points of the immunizations are indicated on each graph. Error bars represent the standard deviation of mean serum IgG binding to the antigen panel for the RMs in each vaccination group.
(TIF)

S6 Fig Neutralizing IgG purified from the serum of RLk17 and ROa17. IgG was purified from serum at week 55 and 63 from RLk17 and ROa17 and tested for neutralization of the Z1800M T/F Env in the TZM-bl assay. Each graph shows neutralization by serum (normalized by IgG content), purified IgG, and the flow through fraction at a 1:40 dilution (FT). RPz16, an RM from the same immunization group that lacked serum nAb activity, was also included in the analysis, as well as normal RM serum (NMS).
(TIF)

S7 Fig R66M and Z1800M have a tier 2 neutralization phenotype. A vertical point plot depicts neutralization of four previously characterized HIV-1/SHIV Env PV: a clade C tier 1 HIV-1 Env (93MW965.26); two tier 2 HIV-1 Envs from a global panel (clade A 398F1 and CRF01_AE CNE55); and a SHIV Env (1157 PV) cloned from clade C SHIV1157pd3N4 challenge stock with a tier 2 phenotype. The clade C tier 2 SHIV1157pd3N4 challenge stock grown in RM PBMC (SHIV.1157) was also included. Z1800M and R66M T/F Env PV were included as the unknowns with respect to neutralization tier. Each Env PV/virus was tested for susceptibility to neutralization by CD4-induced epitope mAb 17b, HIVIG, V3 bnAb 447-52D, individual plasma samples from five Rwandan (R) and five Zambian (Z) HIV+ individuals, and pools of plasma from a group of Rwandan and Zambian HIV+ individuals. The graph depicts residual infectivity when each mAb was tested at 5 μg/ml and each plasma/serum sample diluted to 1:100. Lower values indicate greater susceptibility to neutralization. A two-way ANOVA with Dunnett’s correction for multiple comparisons was performed and revealed that all Envs, including the Z1800M and R66M T/F Envs, were statistically different from the tier 1 Env 93MW965.26 (all p<0.0001).
(TIF)

S8 Fig Neutralization activity controls. Negative controls including non-neutralizing serum from Z1800M T/F gp120 immunized animal RPz16 (wk 55 and wk63) and normal (naïve) RM serum **(A)** as well as positive control bnAb VRC01 **(B)** were tested in the TZM-bl assay against the Z1800M T/F Env, 5-month Envs D10 and D11, and Env chimeras in which the T/F Env contained fragments from D10 and D11 or mutants in which changes in V5 from D10 and D11 were introduced into the T/F Env.
(TIF)

S9 Fig Characteristics of autologous neutralizing mAbs from Z1800M. **(A)** 64 mAbs were isolated from Z1800M at 7 months post-infection by sorting B cells with the autologous T/F Env gp120. The mAbs were tested for neutralization of the autologous T/F Env PV at a concentration of 10 μg/ml. Percent viral infectivity is shown on the y-axis relative to no test mAb. Three neutralizing mAbs from Z1800M are indicated by color. VRC01 and EM4C04 were used as positive and negative controls, respectively. **(B)** Genetic characteristics of the 3 Z1800M neutralizing mAbs are shown, using IMGT VQuest. mAbs 1A8 and 2H10 are considered clonally related (shaded gray). **(C)** A binning competition assay was conducted on the Octet Red96 between each of the Z1800M neutralizing mAbs and reference bnAbs VRC01, 3074, and PGT121, as well as the autologous 1A8. The percent residual binding of the reference mAb in the presence of the competitor is shown, with red indicating strong competition and blue representing weak or no competition.
(TIF)

S10 Fig Selection of antigen specific B cells from RLk17 that recognize wildtype but not V5 mutated gp120. **(A)** The gating strategy used to isolate single B cells from cryopreserved week 63 PBMC that bound to Z1800M T/F gp120 but not the same protein when V5 was mutated is shown. **(B)** Live CD20+ IgG+ B cells that were positive for Z1800M T/F gp120-AF647 and negative for D11 mutated V5 gp120-PE were selected (upper left). **(C)** Germlines from all VH sequences isolated from single B cells selected for binding to Z1800M T/F gp120 (TF) or differential binding to the T/F but not the D11 V5 mutated protein (V5) are shown in donut plots. The total number of VH sequences is indicated. Enrichment of the neutralizing clonotype derived from IGHV4-79*02 is shown by the arrows.
(TIF)

S11 Fig Glycan Encounter Factor over CD4bs epitope residues. Comparison between BG505 and Z1800M T/F Env SOSIP models. Amino acid residues based on HXB2 are shown.
(TIF)

S1 Appendix Glycoproteomics workflow, methods and spectra. (DOCX)
