# Supplementary figures and images for: A neutralizing antibody target in early HIV-1 infection was recapitulated in rhesus macaques immunized with the transmitted/founder envelope sequence
Source: PLoS Pathog. 2022 May 3;18(5):e1010488. doi: 10.1371/journal.ppat.1010488 (PMC9106183; doi:10.1371/journal.ppat.1010488)

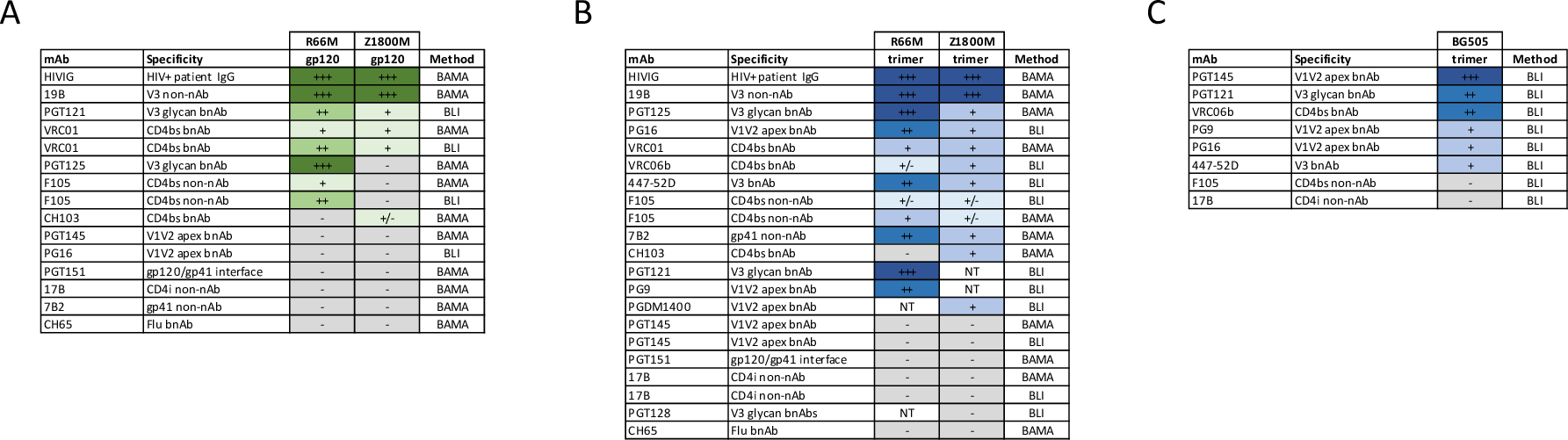

Supplement: S1 Fig — The Z1800M and R66M T/F Env gp120 proteins (A), the Z1800M UFO trimer and R66M NFL trimer (B), and the BG505 NFL trimer as a process control (C) were analyzed by measuring the binding of HIV bnAbs, non-neutralizing mAbs, and HIVIG through BAMA or BLI. For the BLI experiments, which were conducted on an Octet Red96, each antibody was immobilized on a biosensor at a concentration of 10 μg/ml (A) or 25 μg/ml (B) and the sensors were immersed in varying molarities of each protein. The gp120 proteins were tested at concentrations ranging from 0.1875 to 1.5 μM and the trimers were tested at concentrations ranging from 0.037 to 1 μM. The individual sensorgrams are shown in S2 Fig. For the BAMA experiments, the untagged immunogen proteins were directly coupled to polystyrene beads and antibody binding was measured using mouse anti-human IgG-biotin primary followed by a streptavidin-PE secondary detection reagent, which was quantified by fluorescence intensity. The BAMA fluorescence units are shown in S3 Fig. In panels A-C, the antibody, binding specificity, and detection method is shown for each antibody-protein combination. The color intensity and number of plus signs indicate the relative strength of binding; gray boxes with a dash indicate that binding was below detection; NT indicates ‘not tested’. Some antibody-protein combinations (e.g. F105, VRC01, 17b) were analyzed using both methods and the results of both are shown. (TIF) [file ppat.1010488.s001.tif]

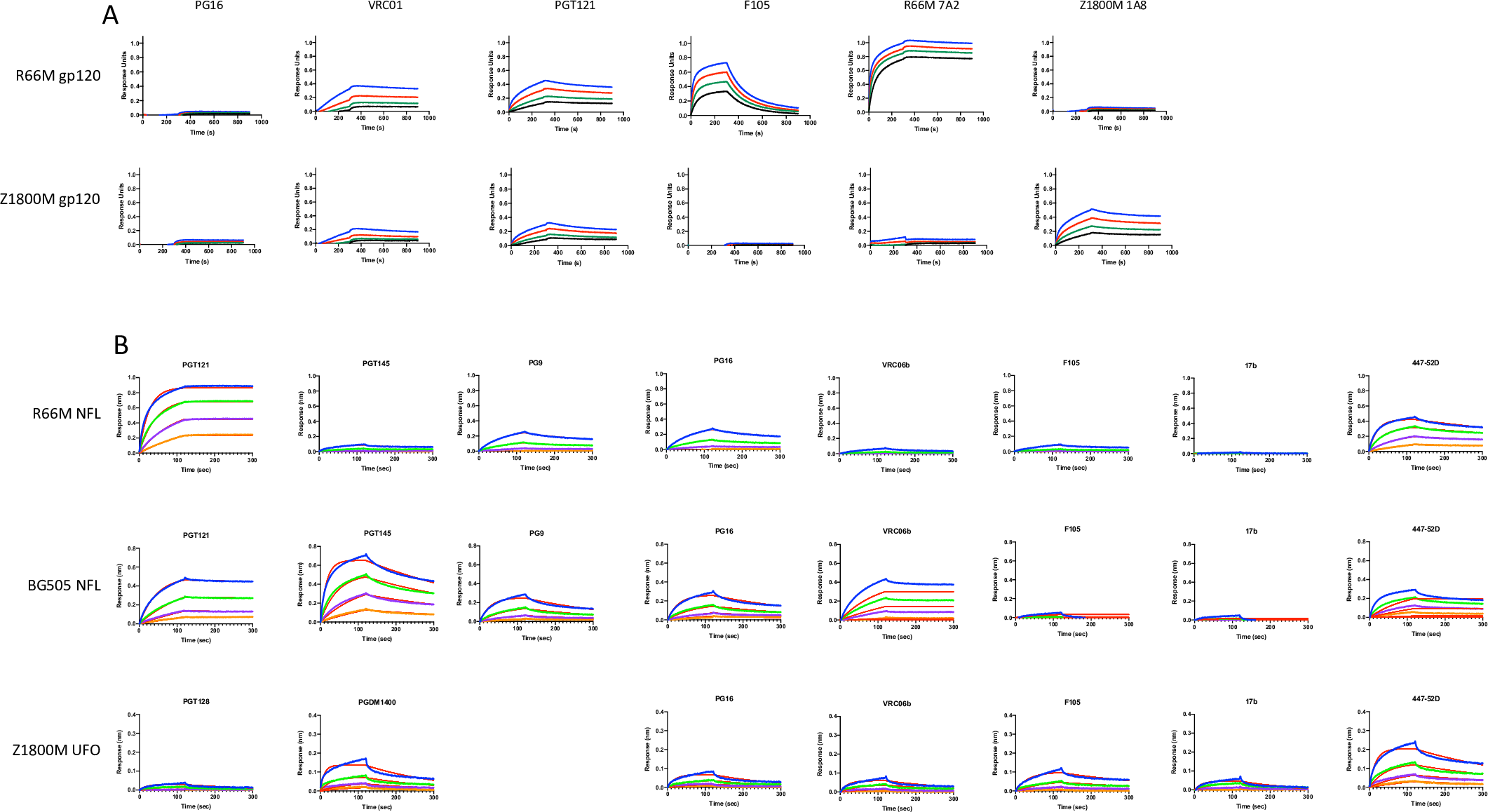

Supplement: S2 Fig — (A) The R66M and Z1800M gp120 immunogen proteins were analyzed by BLI, using an Octet Red96, using HIV bnAbs PG16, VRC01, PGT121, as well as non-neutralizing CD4 binding site mAb F105, and autologous non-neutralizing R66M mAb7A2 and autologous neutralizing Z1800M mAb 1A8 as controls. Each antibody was immobilized on a biosensor at a concentration of 10 μg/ml and the sensors were immersed in varying molarities of each protein. The gp120 proteins were tested at concentrations ranging from 0.1875 to 1.5 μM. (B) The R66M NFL trimer and Z1800M UFO trimer were also analyzed by BLI, using V3-glycan bnAbs PGT121, PGT128; V1V2 apex bnAbs PGT145, PGDM1400, PG9, PG16; CD4 binding site bnAb VRC06b and non-neutralizing mAb F105; CD4-induced non-neutralizing mAb 17b; and V3 bnAb 447-52D. The trimers were tested at concentrations ranging from 0.037 to 1 μM with antibody immobilized at 25 μg/ml. Response units are indicated on the y axis and are plotted against time on the x axis. Related to Fig 1. (TIF) [file ppat.1010488.s002.tif]

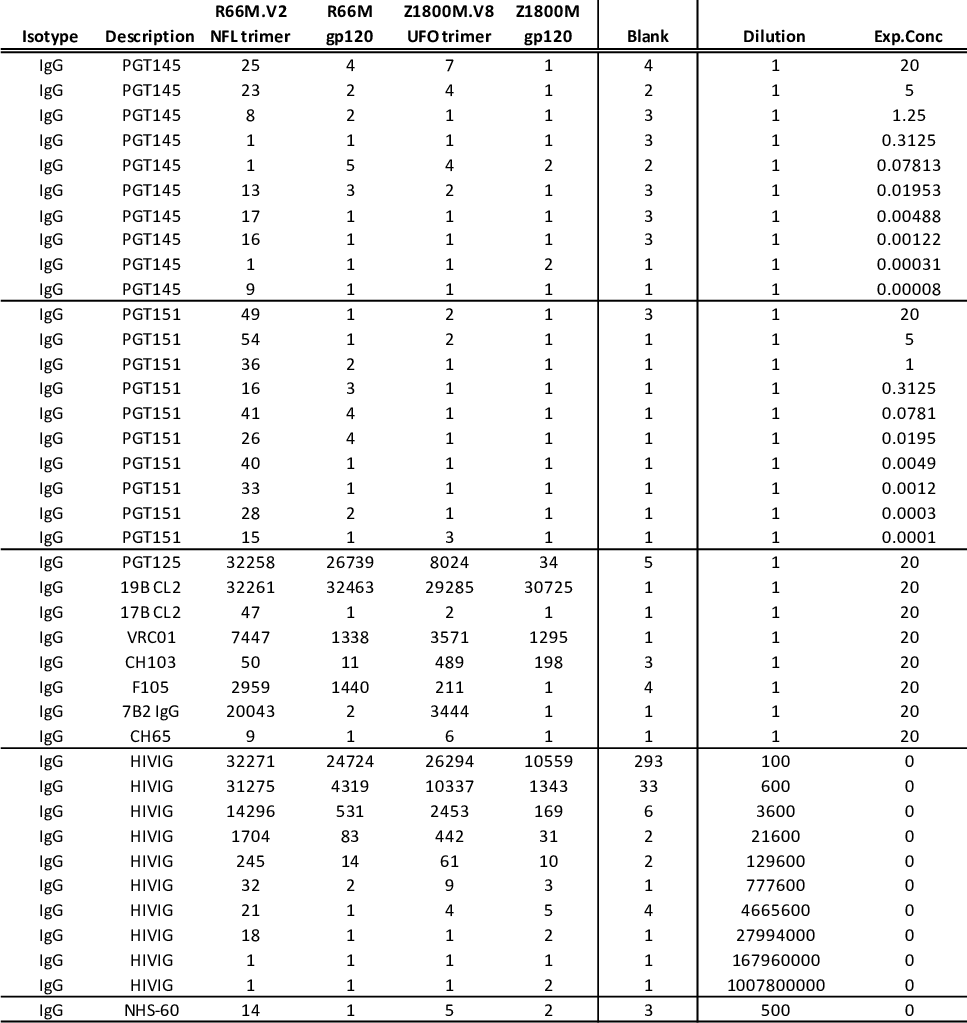

Supplement: S3 Fig — The fluorescence intensity data generated via antibody binding to the immunogen proteins in the BAMA assay is shown. Proteins were analyzed using V1V2 apex bnAb PGT145, gp120-gp41 interface bnAb PGT151, both with serial dilutions starting at 20 μg/ml; a panel of bnAb and non-neutralizing antibodies PGT125, 19B, 17B, VRC01, CH102, F105, 7B2, and an anti-influenza bnAb CH65 at 20 μg/ml; HIVIG at serial dilutions starting at 1:100; and normal human serum (NHS-60) diluted at 1:500 as a negative control. Related to Fig 1. (TIF) [file ppat.1010488.s003.tif]

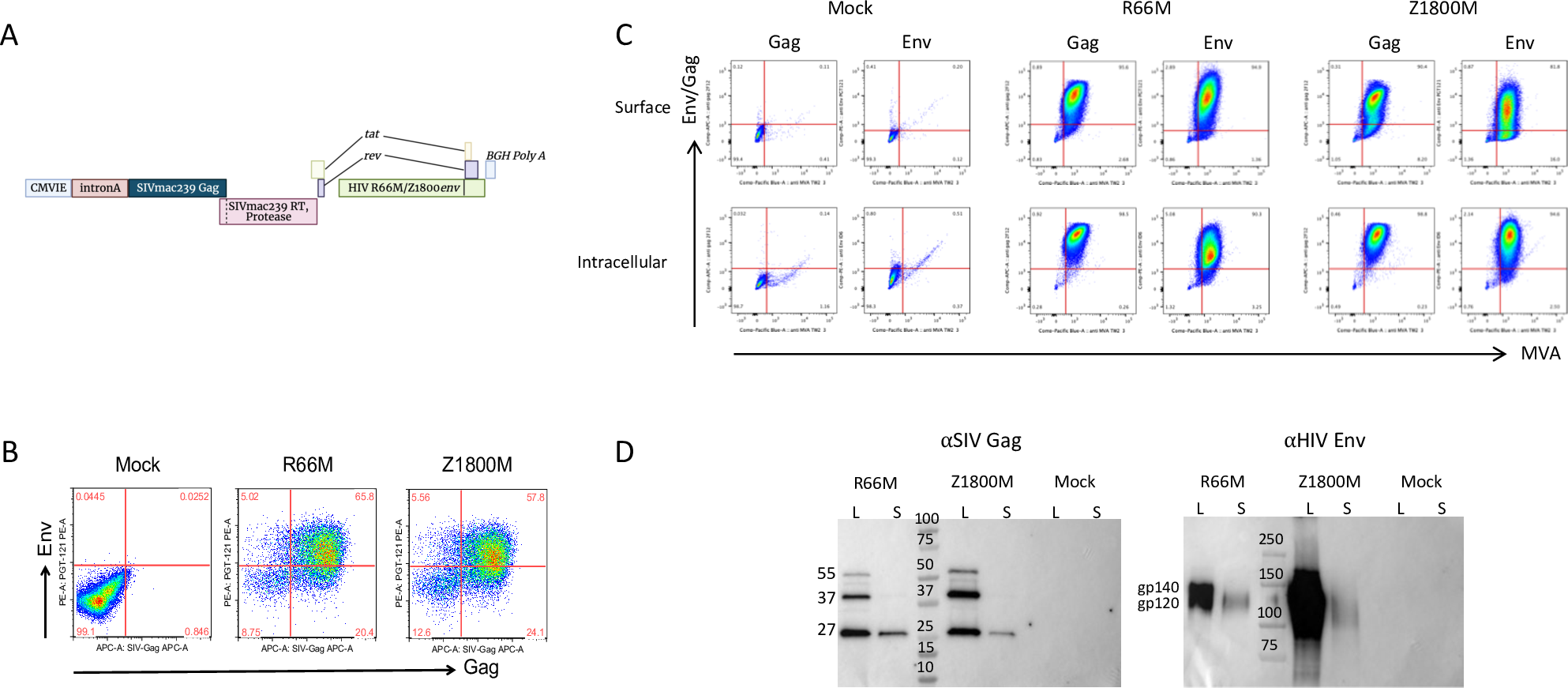

Supplement: S4 Fig — (A) A schematic representation of the recombinant DNA plasmid that expresses SIVmac239 Gag/Pro/Pol and HIV-1 Env is shown. (B) Flow cytometric detection of HIV-1 Env (bnAb PGT121) and SIV Gag (mAb 2F12) expression in R66M and Z1800M T/F Env recombinant DNA plasmid transfected 293T cells. (C) Flow cytometric detection of SIVmac239 Gag and HIV-1 Env in DF-1 cells infected with the R66M and Z1800M T/F Env recombinant MVA vectors. Surface Env was stained using PGT121 and intracellular Env was detected using mAb ID6. Surface and intracellular Gag was detected using mAb 2F12. Gag and Env expression were plotted on live cells infected with rMVA, stained intracellularly for MVA E3 protein (mAb TW2.3, BEI Resources). (D) Lysates (L) and supernatants (S) from rMVA infected DF-1 cells were probed with serum from a BG505 SOSIP-immunized RM to detect HIV-1 Env on western blot and SIV Gag was detected using mAb 2F12. (TIF) [file ppat.1010488.s004.tif]

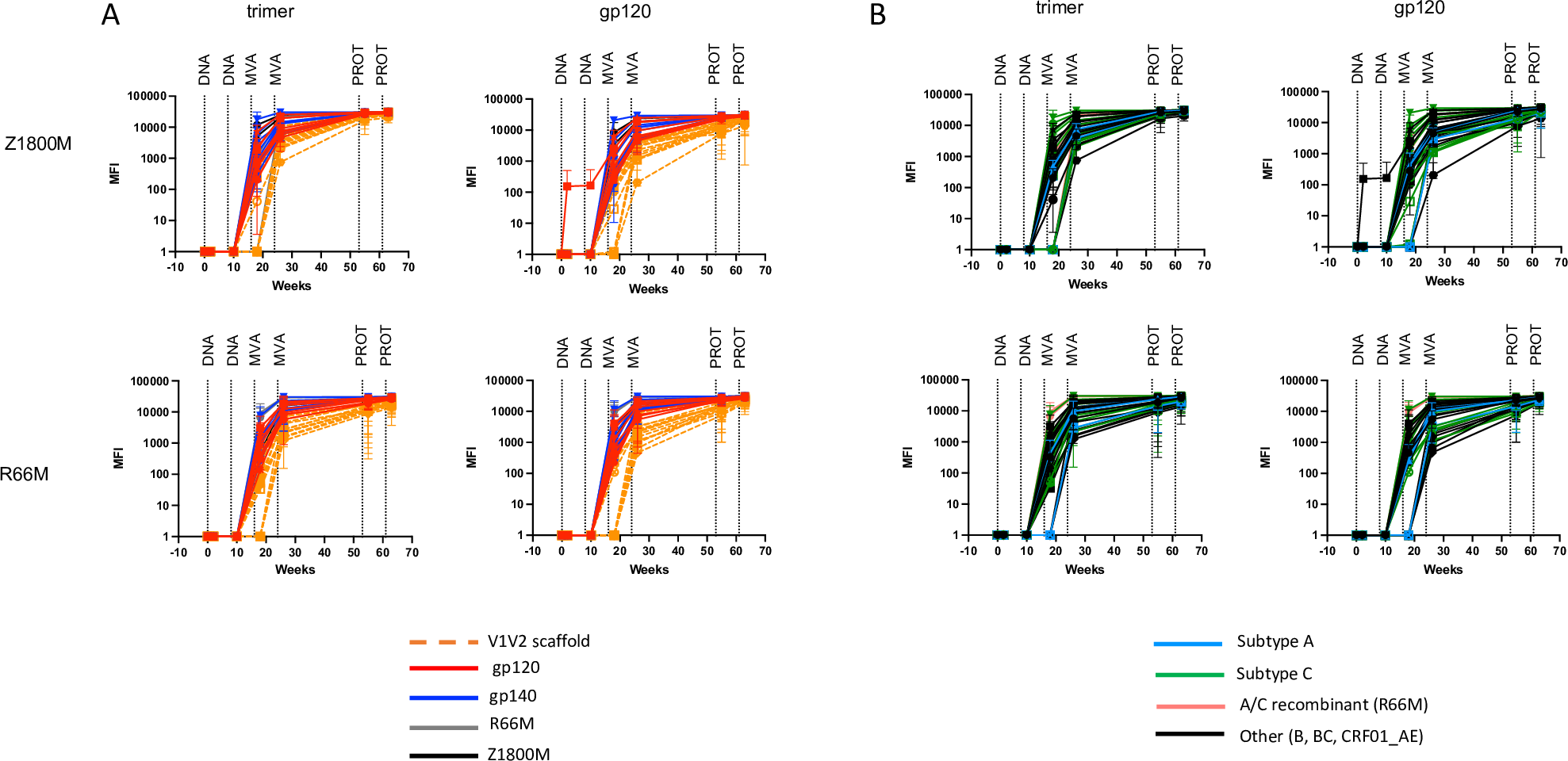

Supplement: S5 Fig — Serum IgG binding to 8 gp120 proteins, 8 gp140 proteins, and 16 scaffolded V1V2 antigens from the BAMA antigen panel, as well as the R66M gp120 and trimer and Z1800M gp120 and trimer, was analyzed with respect to time (weeks). The data was colored according to the type of protein in (A) and the subtype of the Env proteins in (B). The top row contains Z1800M T/F Env immunized RMs that received the trimer or gp120 immunogen, as indicated, and each line represents the mean response of RM in that group against one gp120/gp140/V1V2 binding antigen. The bottom row contains the same information for R66M T/F Env immunized RMs. The mean MFI (mean fluorescence intensity) was calculated for RMs in the four vaccination groups and is plotted on the y-axis on a log10 scale for each binding antigen. The time points of the immunizations are indicated on each graph. Error bars represent the standard deviation of mean serum IgG binding to the antigen panel for the RMs in each vaccination group. (TIF) [file ppat.1010488.s005.tif]

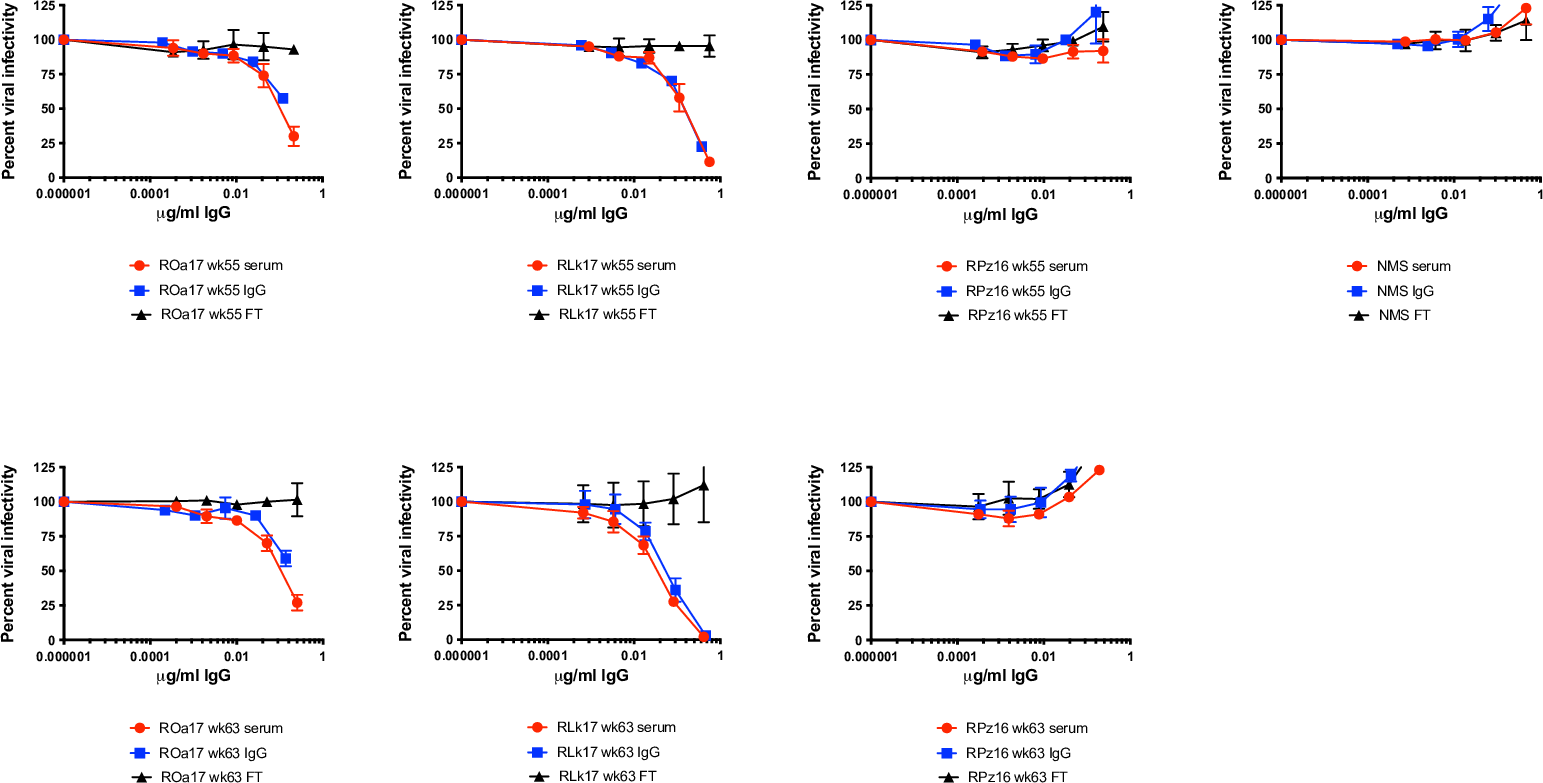

Supplement: S6 Fig — IgG was purified from serum at week 55 and 63 from RLk17 and ROa17 and tested for neutralization of the Z1800M T/F Env in the TZM-bl assay. Each graph shows neutralization by serum (normalized by IgG content), purified IgG, and the flow through fraction at a 1:40 dilution (FT). RPz16, an RM from the same immunization group that lacked serum nAb activity, was also included in the analysis, as well as normal RM serum (NMS). (TIF) [file ppat.1010488.s006.tif]

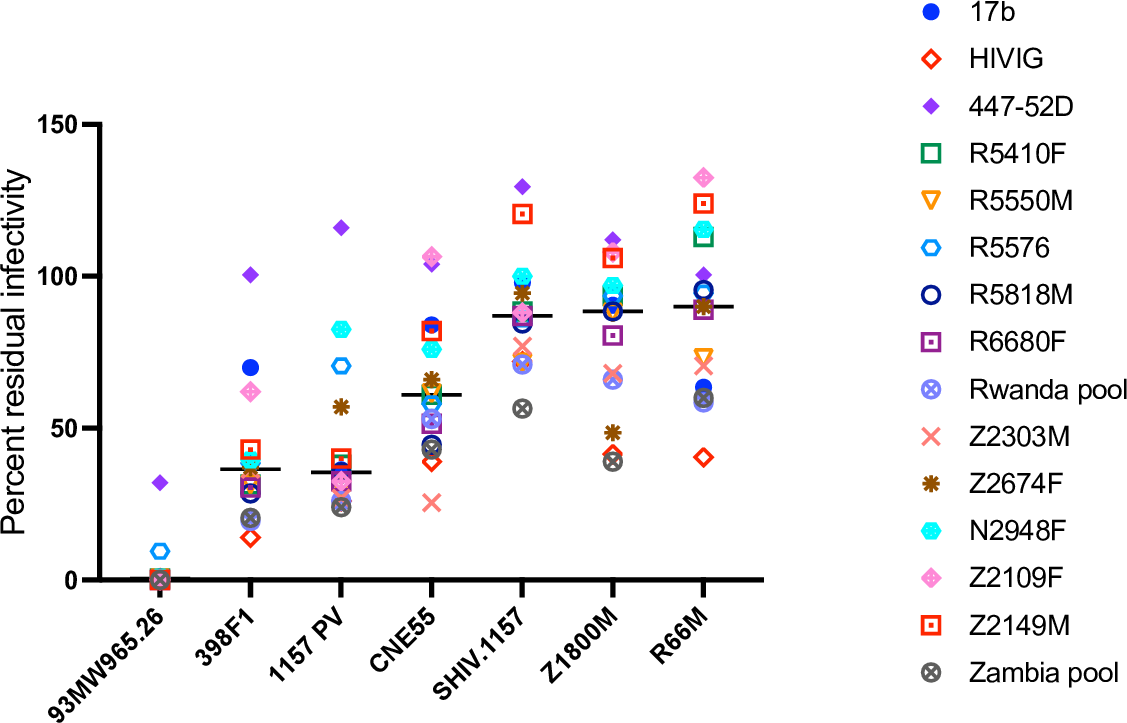

Supplement: S7 Fig — A vertical point plot depicts neutralization of four previously characterized HIV-1/SHIV Env PV: a clade C tier 1 HIV-1 Env (93MW965.26); two tier 2 HIV-1 Envs from a global panel (clade A 398F1 and CRF01_AE CNE55); and a SHIV Env (1157 PV) cloned from clade C SHIV1157pd3N4 challenge stock with a tier 2 phenotype. The clade C tier 2 SHIV1157pd3N4 challenge stock grown in RM PBMC (SHIV.1157) was also included. Z1800M and R66M T/F Env PV were included as the unknowns with respect to neutralization tier. Each Env PV/virus was tested for susceptibility to neutralization by CD4-induced epitope mAb 17b, HIVIG, V3 bnAb 447-52D, individual plasma samples from five Rwandan (R) and five Zambian (Z) HIV+ individuals, and pools of plasma from a group of Rwandan and Zambian HIV+ individuals. The graph depicts residual infectivity when each mAb was tested at 5 μg/ml and each plasma/serum sample diluted to 1:100. Lower values indicate greater susceptibility to neutralization. A two-way ANOVA with Dunnett’s correction for multiple comparisons was performed and revealed that all Envs, including the Z1800M and R66M T/F Envs, were statistically different from the tier 1 Env 93MW965.26 (all p<0.0001). (TIF) [file ppat.1010488.s007.tif]

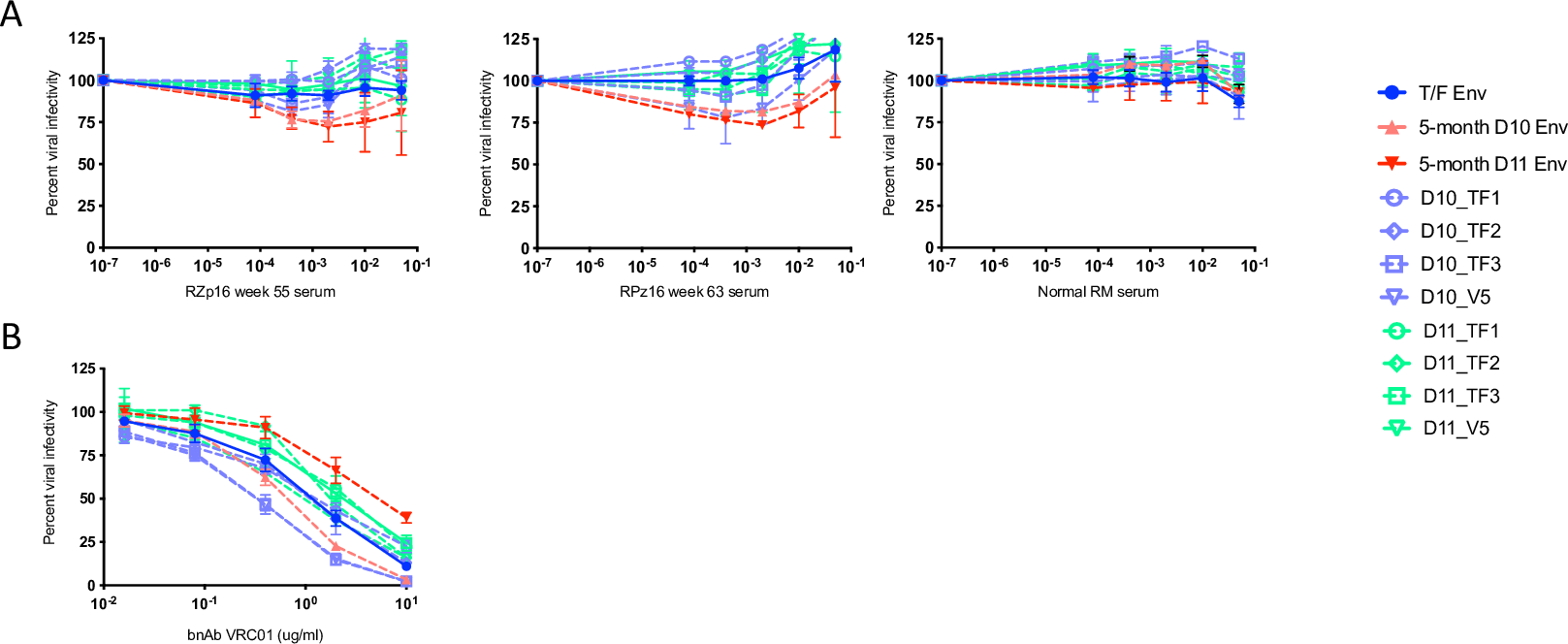

Supplement: S8 Fig — Negative controls including non-neutralizing serum from Z1800M T/F gp120 immunized animal RPz16 (wk 55 and wk63) and normal (naïve) RM serum (A) as well as positive control bnAb VRC01 (B) were tested in the TZM-bl assay against the Z1800M T/F Env, 5-month Envs D10 and D11, and Env chimeras in which the T/F Env contained fragments from D10 and D11 or mutants in which changes in V5 from D10 and D11 were introduced into the T/F Env. (TIF) [file ppat.1010488.s008.tif]

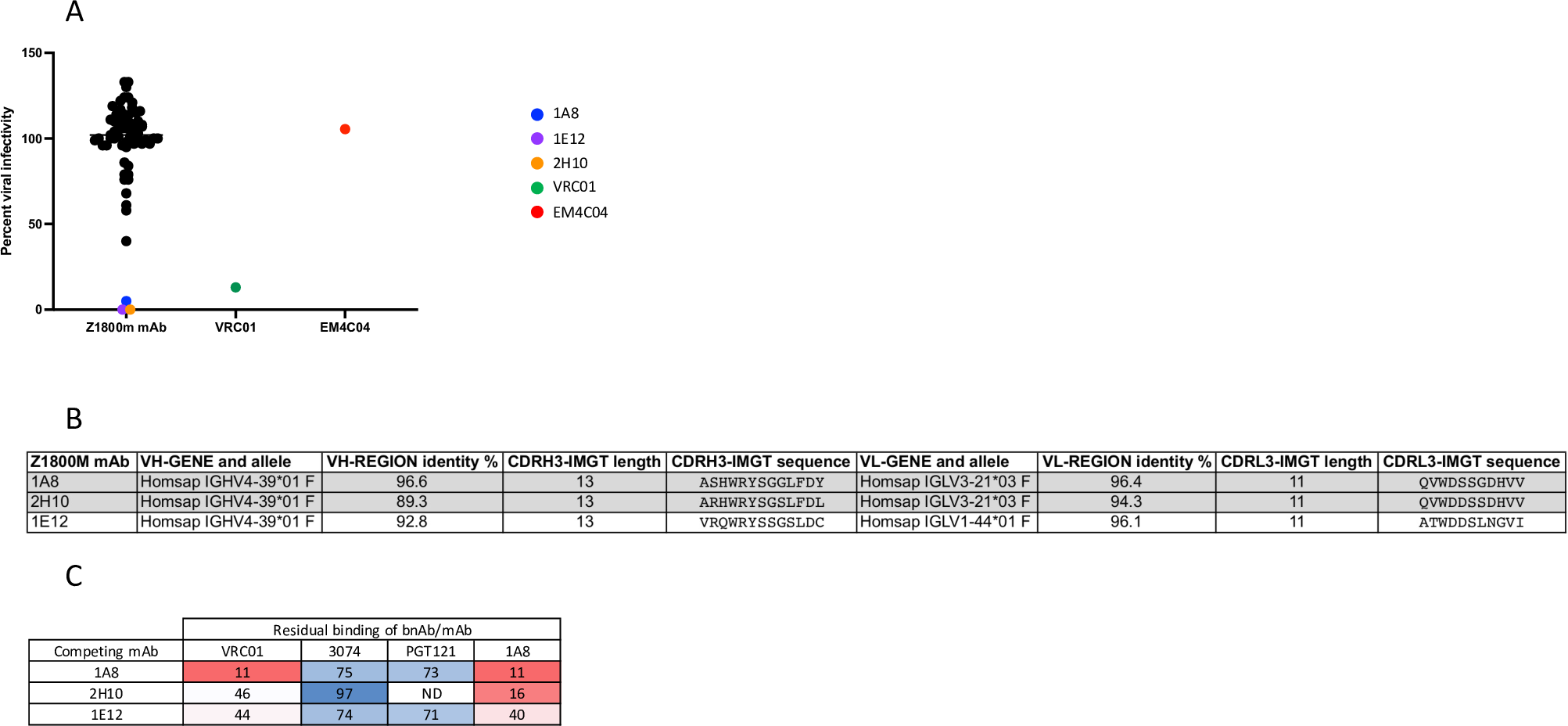

Supplement: S9 Fig — (A) 64 mAbs were isolated from Z1800M at 7 months post-infection by sorting B cells with the autologous T/F Env gp120. The mAbs were tested for neutralization of the autologous T/F Env PV at a concentration of 10 μg/ml. Percent viral infectivity is shown on the y-axis relative to no test mAb. Three neutralizing mAbs from Z1800M are indicated by color. VRC01 and EM4C04 were used as positive and negative controls, respectively. (B) Genetic characteristics of the 3 Z1800M neutralizing mAbs are shown, using IMGT VQuest. mAbs 1A8 and 2H10 are considered clonally related (shaded gray). (C) A binning competition assay was conducted on the Octet Red96 between each of the Z1800M neutralizing mAbs and reference bnAbs VRC01, 3074, and PGT121, as well as the autologous 1A8. The percent residual binding of the reference mAb in the presence of the competitor is shown, with red indicating strong competition and blue representing weak or no competition. (TIF) [file ppat.1010488.s009.tif]

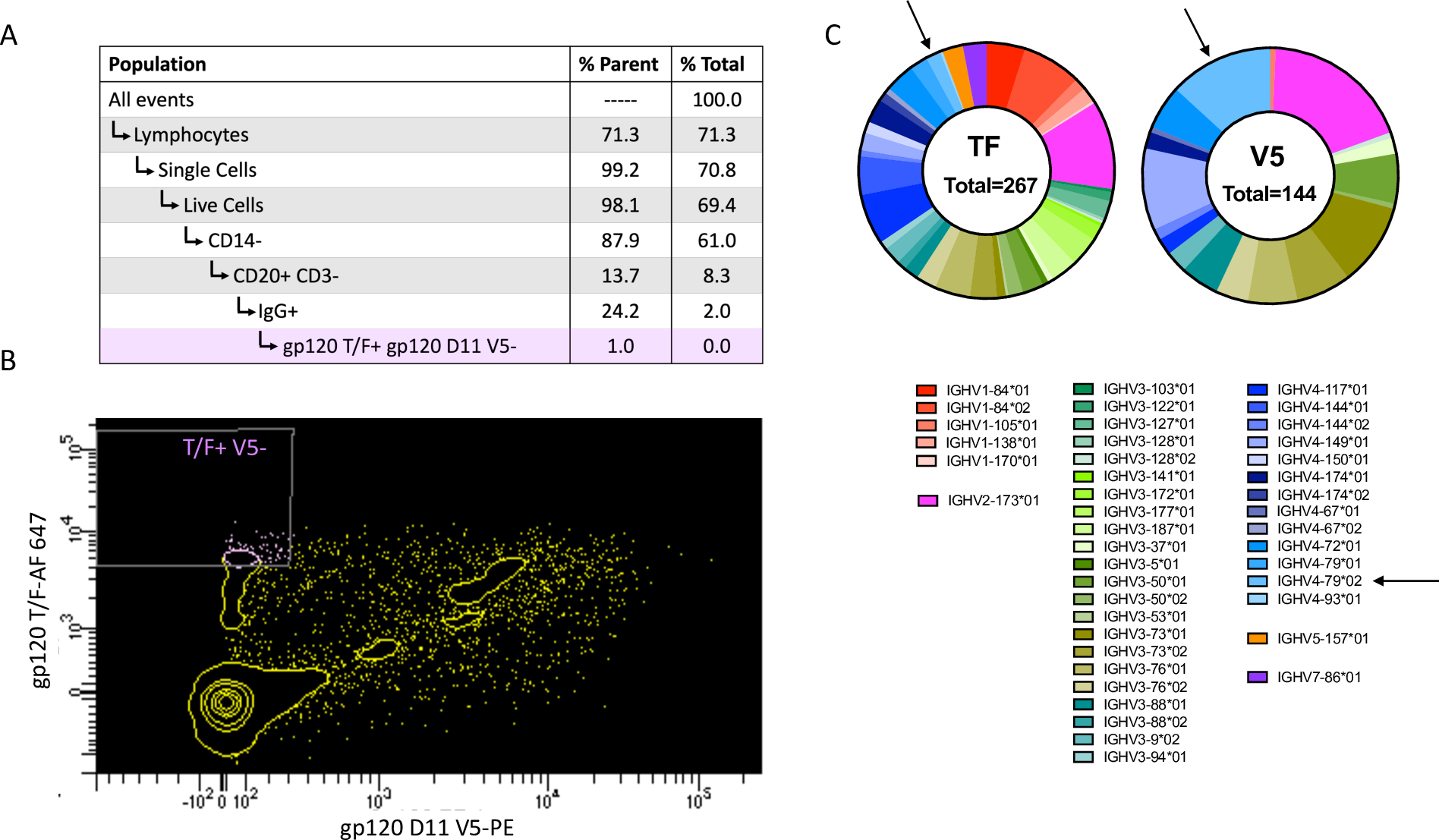

Supplement: S10 Fig — (A) The gating strategy used to isolate single B cells from cryopreserved week 63 PBMC that bound to Z1800M T/F gp120 but not the same protein when V5 was mutated is shown. (B) Live CD20+ IgG+ B cells that were positive for Z1800M T/F gp120-AF647 and negative for D11 mutated V5 gp120-PE were selected (upper left). (C) Germlines from all VH sequences isolated from single B cells selected for binding to Z1800M T/F gp120 (TF) or differential binding to the T/F but not the D11 V5 mutated protein (V5) are shown in donut plots. The total number of VH sequences is indicated. Enrichment of the neutralizing clonotype derived from IGHV4-79*02 is shown by the arrows. (TIF) [file ppat.1010488.s010.tif]

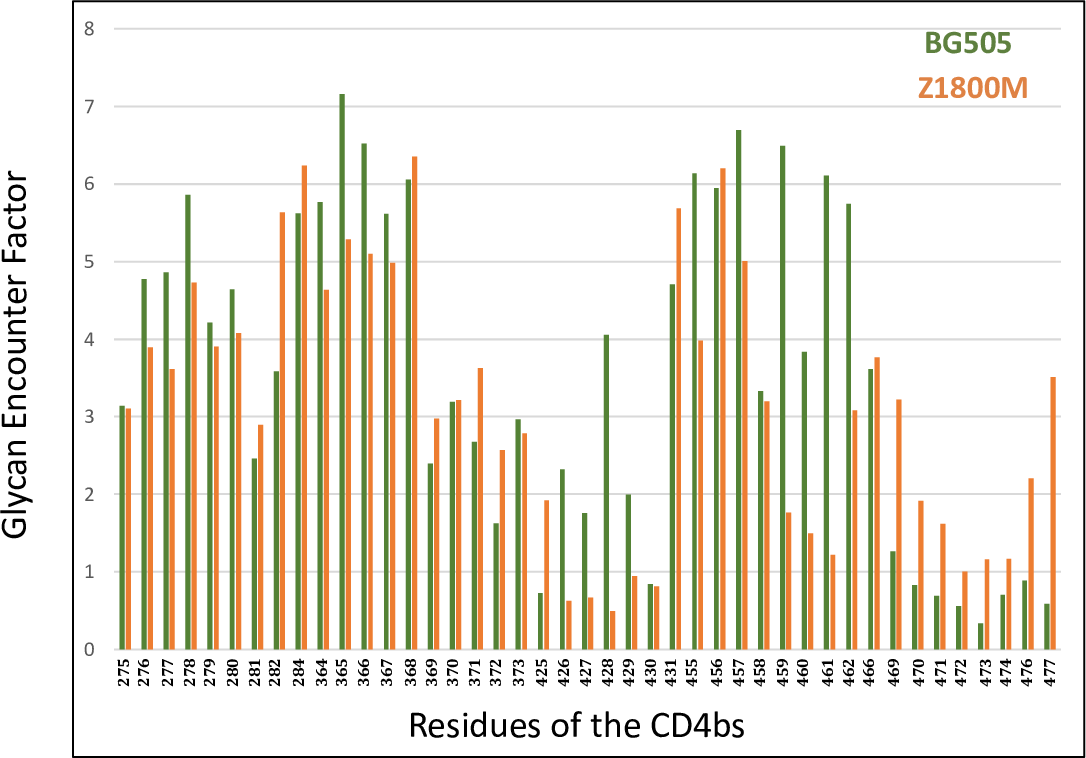

Supplement: S11 Fig — Comparison between BG505 and Z1800M T/F Env SOSIP models. Amino acid residues based on HXB2 are shown. (TIF) [file ppat.1010488.s011.tif]
