# Supplementary material for: A neutralizing antibody target in early HIV-1 infection was recapitulated in rhesus macaques immunized with the transmitted/founder envelope sequence
Source: PLoS Pathog. 2022 May 3;18(5):e1010488. doi: 10.1371/journal.ppat.1010488 (PMC9106183; doi:10.1371/journal.ppat.1010488)
Supplement: S1 Appendix — (DOCX) [file ppat.1010488.s012.docx]

**S1 Supplementary Appendix**

***Glycoproteomic sample preparation, nano-LC-MS/MS data acquisition and data analysis of monomeric Env gp120 Z1800M***

Sequencing-grade modified trypsin and chymotrypsin were purchased from Promega (Madison, WI). Sequencing-grade modified endoproteinase Glu-C was acquired from Roche Diagnostics (Indianapolis, IN).

All other reagents were purchased from Sigma Aldrich unless indicated otherwise. Data analysis was performed using Byonic 2.3 software and manually using Xcalibur 4.2 and GlycoWorkbench 1.1.

**Protease digestion for glycoproteomics of gp120 Z1800M**

The purified and lyophilized monomeric T/F Env gp120 protein Z1800M was dissolved in 50 mM ammonium bicarbonate solution. The protein was reduced in 12 mM dithiothreitol solution, and subsequently alkylated in 30 mM iodoacetamide. In total, three enzymatic digestions were set up for best coverage: (i) tryptic digest for 16 h at 37 °C, (ii) tryptic digest (16 h at 37 °C) followed by a chymotryptic digest at room temperature for 16 h, (iii) tryptic digest (16 h at 37 °C) followed by a digest with endoproteinase Glu-C at 37 °C for 16 h in phosphate buffer. The three digests were individually filtered through 0.2 µm filter and directly analyzed by LC-MS/MS.

**Data acquisition of protein digests using nano-LC-MS/MS**

The glycoprotein digests were analyzed on an Orbitrap Fusion Tribrid mass spectrometer equipped with a nanospray ion source and connected to a Dionex Ultimate 3000 RSLC nano system (Thermo Fisher, Waltham, MA). A pre-packed nano-LC column (Cat. No. 164568, Thermo Fisher, Waltham, MA) of 15 cm length with 75 µm internal diameter (id), filled with 3 µm C18 material (reverse phase) was used for the chromatographic separation of the samples. The precursor ion scan was acquired at 120,000 resolutions in the Orbitrap analyzer and precursors at a time frame of 3 s were selected for subsequent MS/MS fragmentation in the Orbitrap analyzer at 15,000 resolution. The LC-MS/MS runs of each digest were conducted for 72 min. 0.1% formic acid and 80% acetonitrile-0.1 % formic acid was used as mobile phase A and B, respectively, in order to separate the glycopeptides. The intensity threshold for triggering an MS/MS event was set to 2000 counts, and monoisotopic precursor selection was enabled. MS/MS fragmentation was conducted with a stepped HCD (Higher-energy Collisional Dissociation) product triggered CID (Collision-Induced Dissociation) (HCDpdCID) program. Charge state screening was enabled, and precursors with unknown charge state or a charge state of +1 were excluded (positive ion mode). Dynamic exclusion was enabled for an exclusion duration of 30 s after 2x detection within 20 s.

**Data analysis of glycoproteins**

The LC-MS/MS spectra of all three enzymatic digests of the monomeric gp120 Z1800M mutant were searched against the FASTA sequence using the Byonic software 2.3 by choosing appropriate peptide cleavage sites (non-specific cleavage option enabled). Carbamidomethylation of cysteine was set as a fixed modification, whereby oxidation of methionine and common human N‑glycans found in plasma (variable) were set as variable modifications. The LC-MS/MS spectra were also analyzed manually for the glycopeptides with the support of the Thermo Fisher Xcalibur 4.2 software, GlycoWorkbench 1.1., GlycoMod tool, Byos v4.2 and ProteinProspector v6.2.1. The HCDpdCID MS^2^ spectra of glycopeptides were evaluated for the glycan neutral loss pattern, oxonium ions and glycopeptide fragmentations to assign the sequence and the presence of glycans in the glycopeptides. **Additional Figure A**, showing the glycoproteomics workflow, was created with the help of BioRender.com.

**Additional Figures:**

**Analysis of the monomeric gp120 Z1800M:**


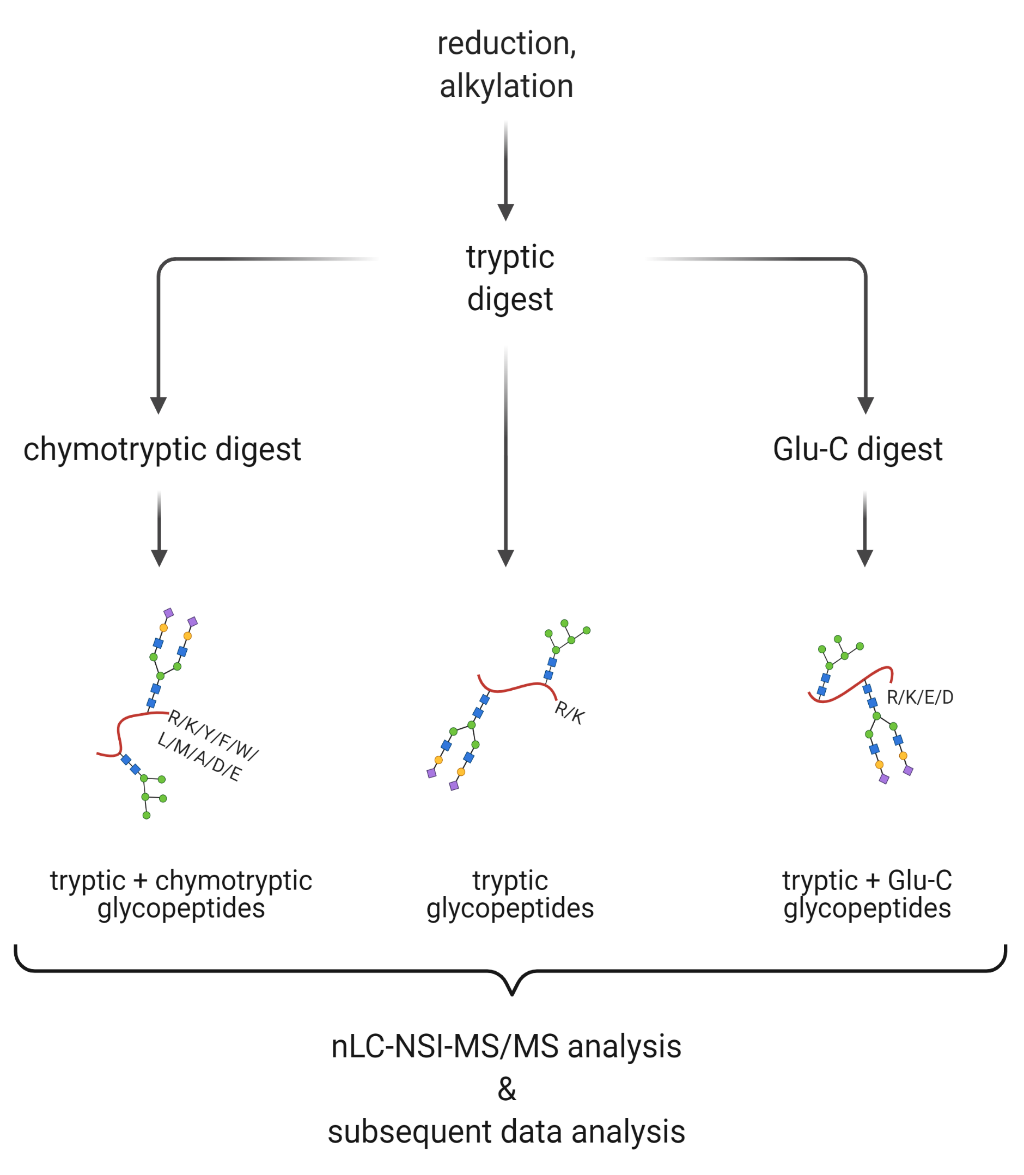


**Figure A.** Glycoproteomic workflow of the monomeric gp120 Z1800M.

(created with BioRender.com).


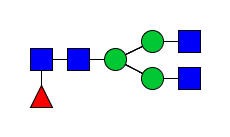

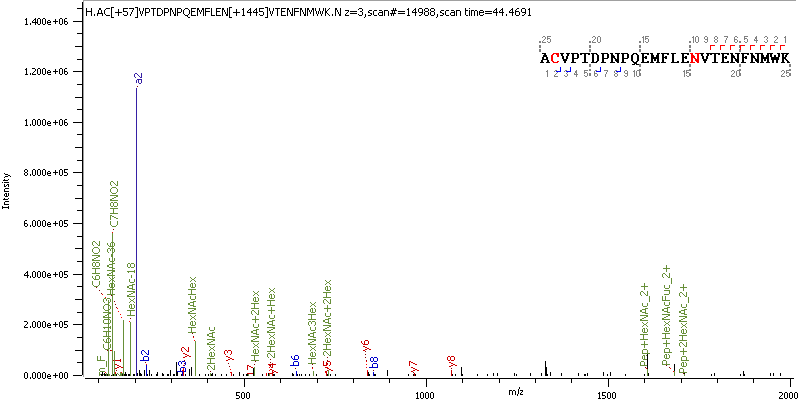


**Figure B.** Annotated HCD MS2 spectrum of N-glycopeptide ^72^ACVPTDPNPQEMFLENVTENFNMWK^96^, with carbamidomethylation of the cysteine (C) and the most abundant glycoform GlcNAc_2_Fuc_1_Man_3_GlcNAc_2_ at glycan site N87, derived from the tryptic digest of gp120 Z1800M.


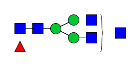

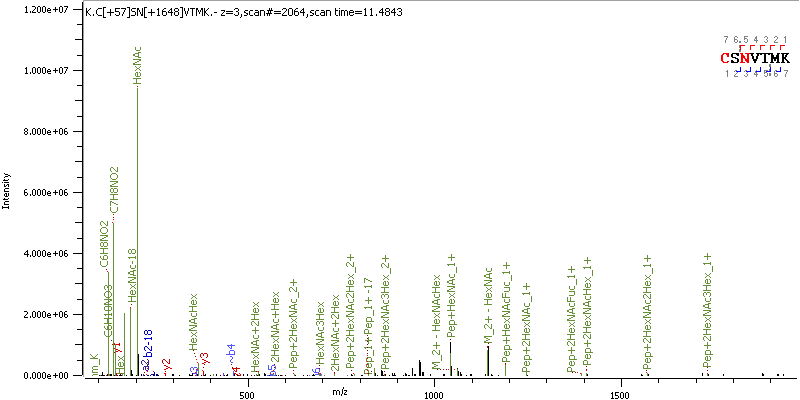


**Figure C.** Annotated HCD MS2 spectrum of N-glycopeptide ^130^CSNVTMK^136^, with carbamidomethylation of the cysteine (C) and the most abundant glycoform GlcNAc_3_Fuc_1_Man_3_GlcNAc_2_ at glycan site N132, derived from the tryptic digest of gp120 Z1800M.


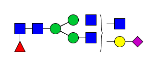

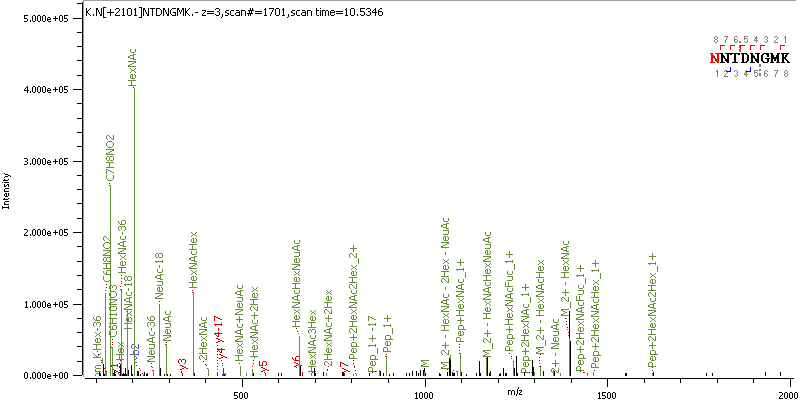


**Figure D.** Annotated HCD MS2 spectrum of N-glycopeptide ^137^NNTDNGMK^144^, with the most abundant glycoform Neu5Ac_1_Gal_1_GlcNAc_3_Fuc_1_Man_3_GlcNAc_2_ at glycan site N137, derived from the tryptic digest of gp120 Z1800M.


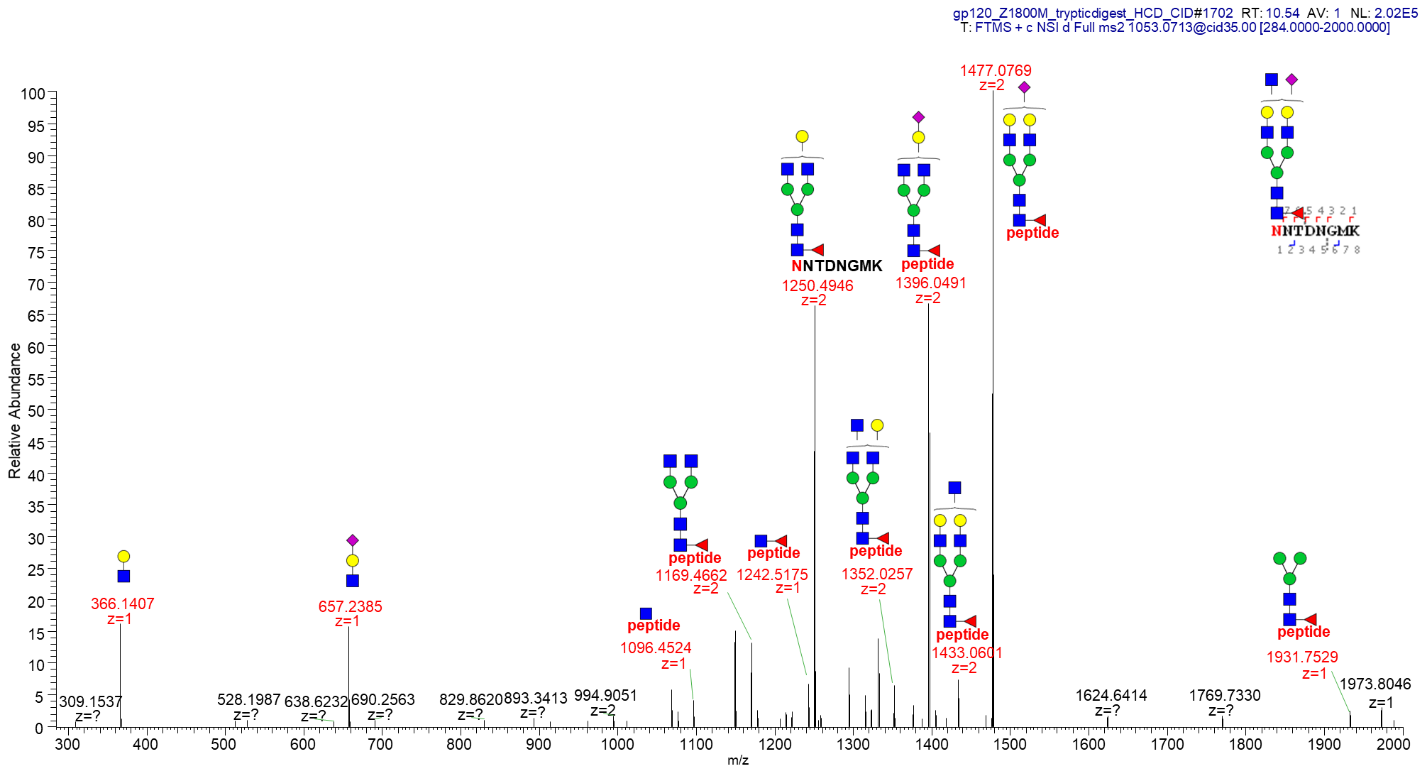


**Figure E.** CID MS2 spectrum of N-glycan Neu5Ac_1_Gal_2_GlcNAc_3_Fuc_1_Man_3_GlcNAc_2_ at glycan site N137 from gp120 Z1800M.


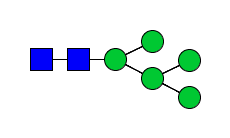

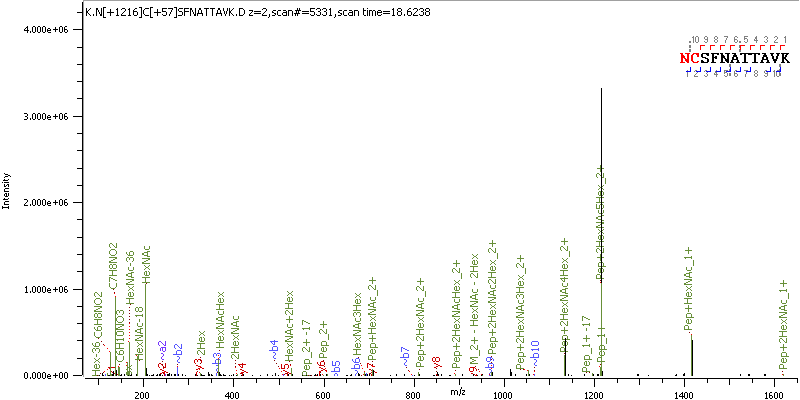


**Figure F.** Annotated HCD MS2 spectrum of N-glycopeptide ^145^NCSFNATTAVK^155^, with carbamidomethylation of the cysteine (C) and the most abundant glycoform Man_5_GlcNAc_2_ at glycan site N145, derived from the tryptic and subsequent chymotryptic digest of gp120 Z1800M.


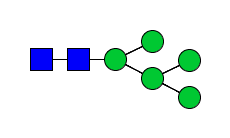

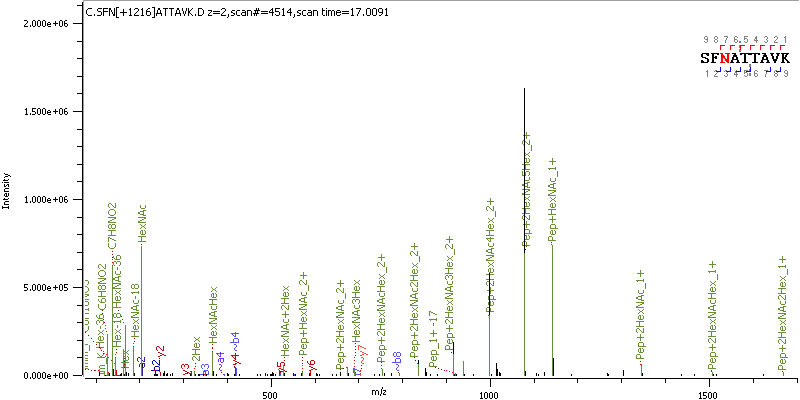


**Figure G.** Annotated HCD MS2 spectrum of N-glycopeptide ^147^SFNATTAVK^148^, with the most abundant glycoform Man_5_GlcNAc_2_ at glycan site N149, derived from a semi-specific cleavage in the tryptic digest of gp120 Z1800M.

**
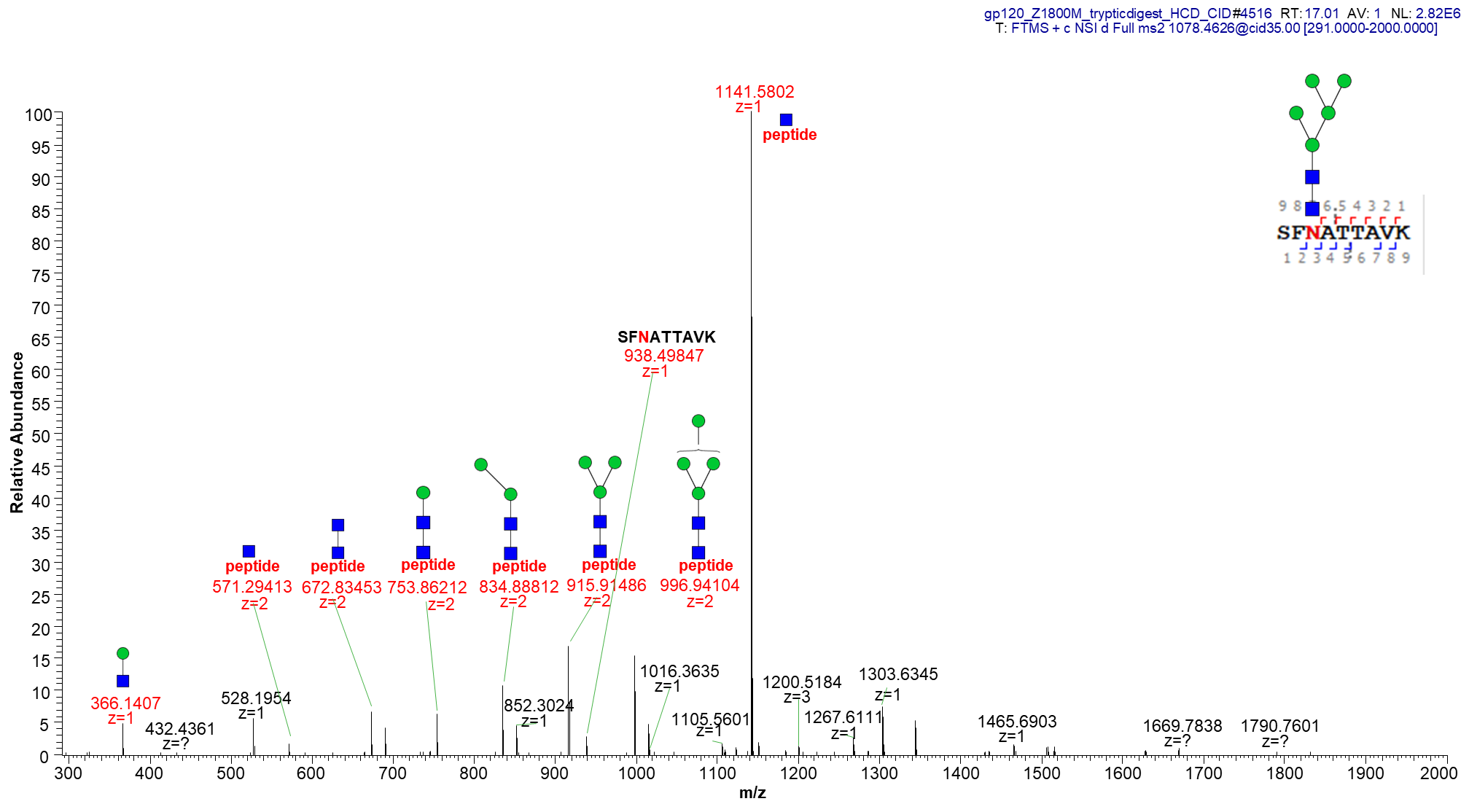
Figure H.** CID MS2 spectrum of N-glycan Man_5_GlcNAc_2_ at glycan site N149 from gp120 Z1800M.


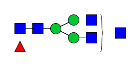

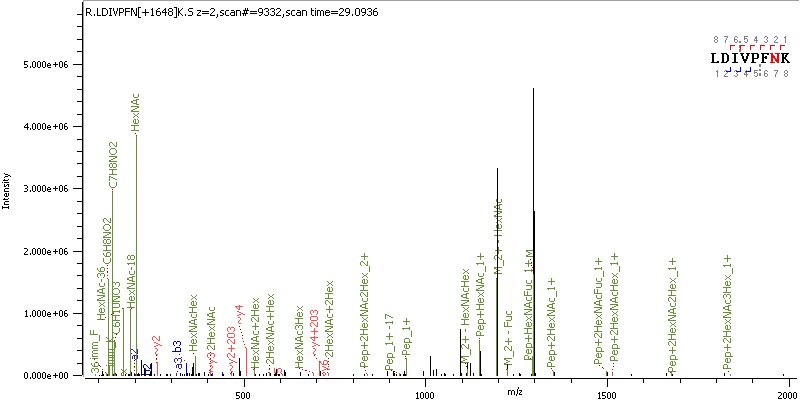


**Figure I.** Annotated HCD MS2 spectrum of N-glycopeptide ^168^LDIVPFNK^175^, with the most abundant glycoform GlcNAc_3_Fuc_1_Man_3_GlcNAc_2_ at glycan site N174, derived from the tryptic digest of gp120 Z1800M.


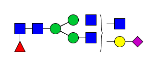

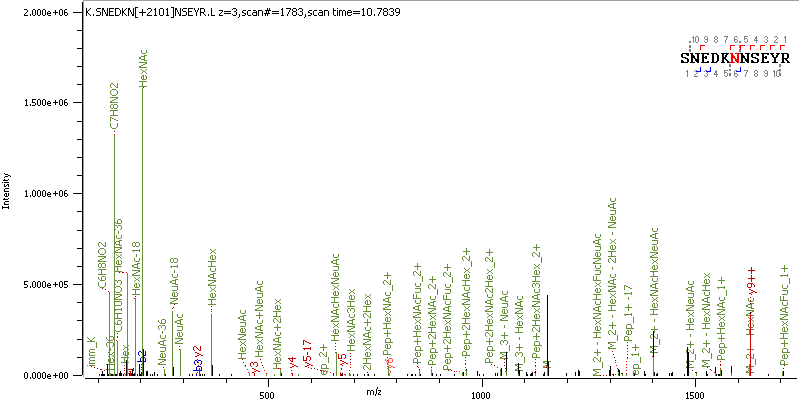


**Figure J.** Annotated HCD MS2 spectrum of N-glycopeptide ^176^SNEDKNNSEYR^186^, with the most abundant glycoform Neu5Ac_1_Gal_1_GlcNAc_3_Fuc_1_Man_3_GlcNAc_2_ at glycan site N181, derived from the tryptic digest of gp120 Z1800M.


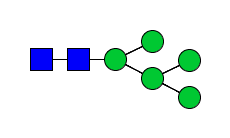

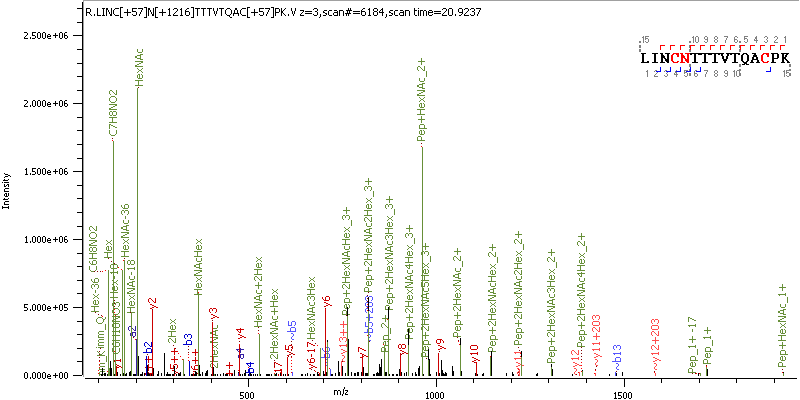


**Figure K.** Annotated HCD MS2 spectrum of N-glycopeptide ^187^LINCNTTTVTQACPK^201^, with carbamidomethylation of both cysteines (C) and the most abundant glycoform Man_5_GlcNAc_2_ at glycan site N191, derived from the tryptic digest of gp120 Z1800M.


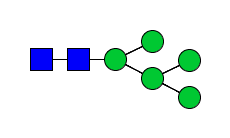

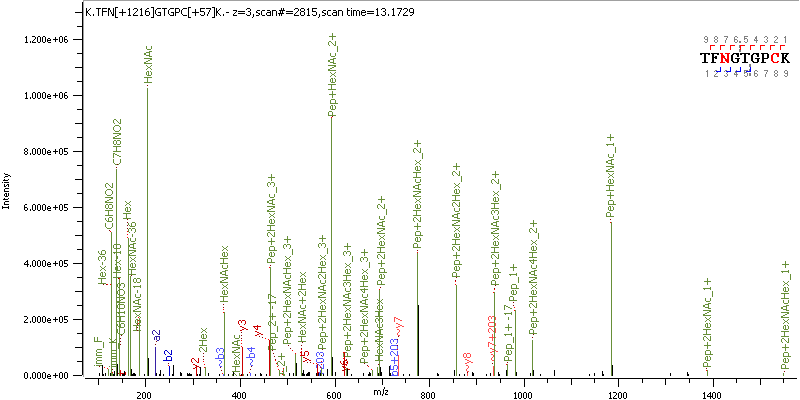


**Figure L.** Annotated HCD MS2 spectrum of N-glycopeptide ^226^TFNGTGPCK^234^, with carbamidomethylation of the cysteine (C) and the most abundant glycoform Man_5_GlcNAc_2_ at glycan site N228, derived from the tryptic with subsequent GluC digest of gp120 Z1800M.


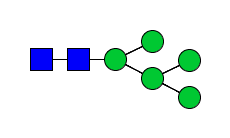

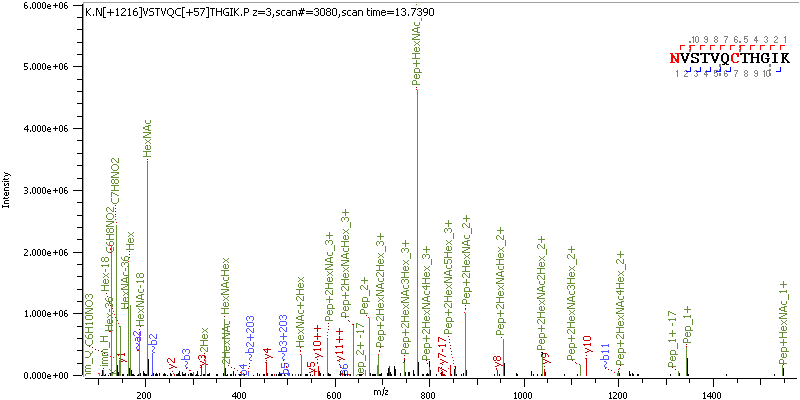


**Figure M.** Annotated HCD MS2 spectrum of N-glycopeptide ^235^NVSTVQCTH^243^, with carbamidomethylation of the cysteine (C) and the most abundant glycoform Man_5_GlcNAc_2_ at glycan site N235, derived from the tryptic digest of gp120 Z1800M.


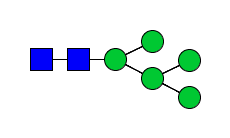

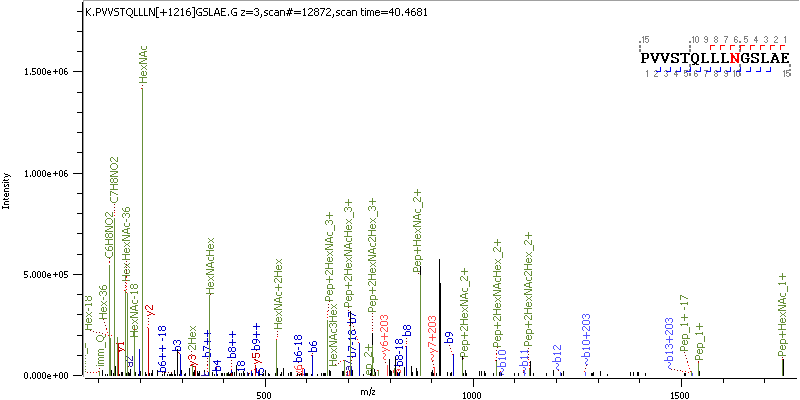


**Figure N.** Annotated HCD MS2 spectrum of N-glycopeptide ^247^PVVSTQLLLNGSLAE^261^, with the most abundant glycoform Man_5_GlcNAc_2_ at glycan site N256, derived from the tryptic with subsequent GluC digest of gp120 Z1800M.


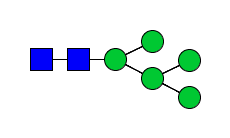

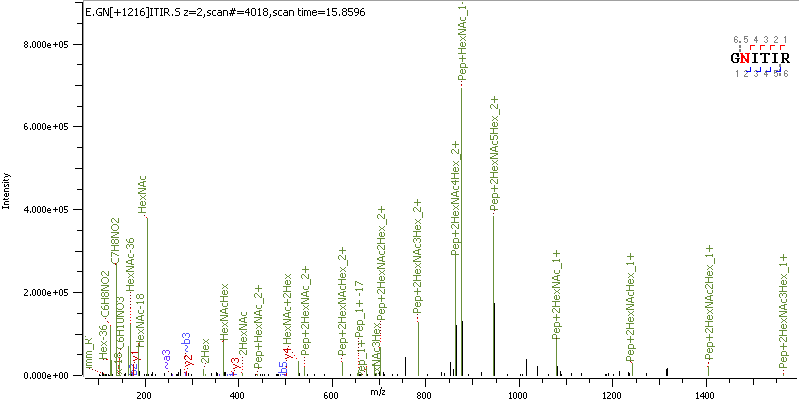


**Figure O.** Annotated HCD MS2 spectrum of N-glycopeptide ^262^GNITIR^267^, with the most abundant glycoform Man_5_GlcNAc_2_ at glycan site N263, derived from the derived from the tryptic with subsequent GluC digest of gp120 Z1800M. This glycosite is part of the high mannose patch.


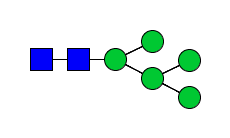

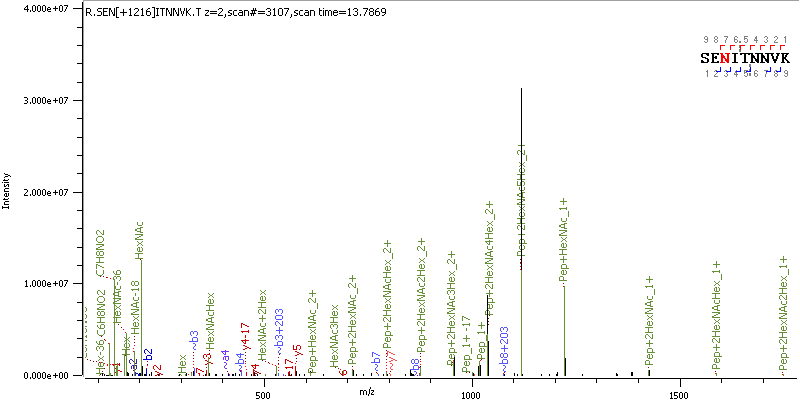


**Figure P.** Annotated HCD MS2 spectrum of N-glycopeptide ^268^SENITNNVK^276^, with the most abundant glycoform Man_5_GlcNAc_2_ at glycan site N270, derived from the tryptic digest of gp120 Z1800M.


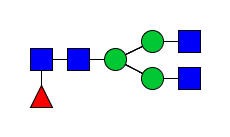

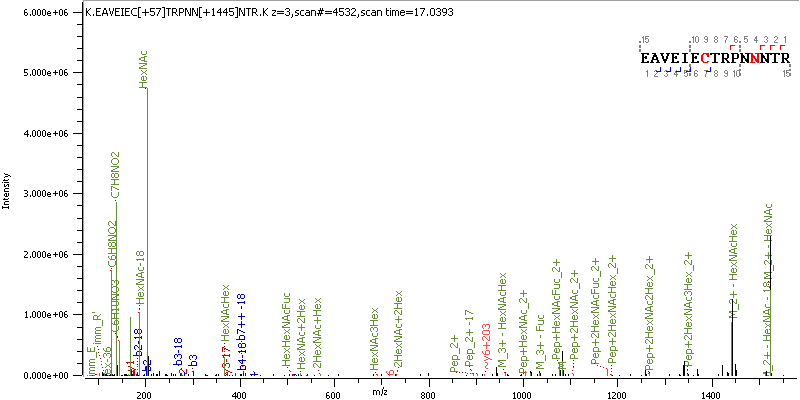


**Figure Q.** Annotated HCD MS2 spectrum of N-glycopeptide ^284^EAVEIECTRPNNNTR^298^, with carbamidomethylation of the cysteine (C) and the most abundant glycoform GlcNAc_2_Fuc_1_Man_3_GlcNAc_2_ at glycan site N295, derived from the tryptic digest of gp120 Z1800M.


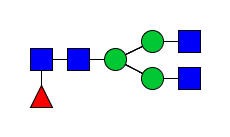

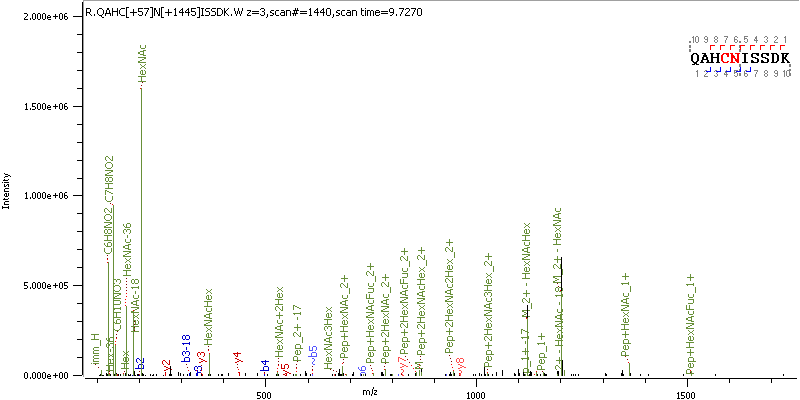


**Figure R.** Annotated HCD MS2 spectrum of N-glycopeptide ^320^QAHCNISSDK^329^, with carbamidomethylation of the cysteine (C) and the most abundant glycoform GlcNAc_2_Fuc_1_Man_3_GlcNAc_2_ at glycan site N324, derived from the tryptic digest of gp120 Z1800M.


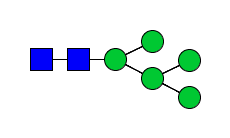

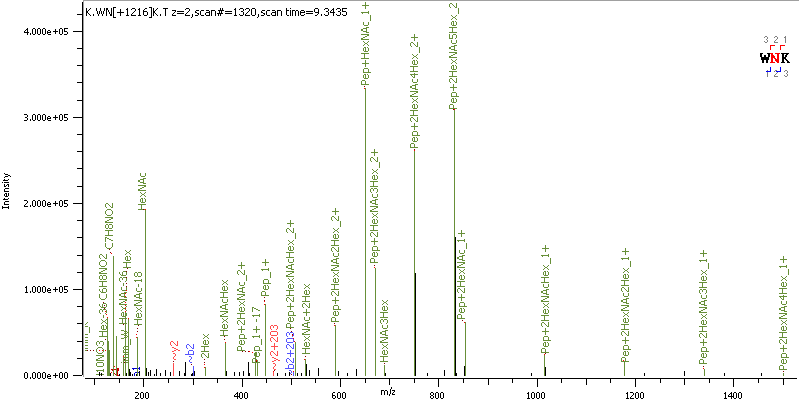


**Figure S.** Annotated HCD MS2 spectrum of N-glycopeptide ^330^WNK^332^, with the most abundant glycoform Man_5_GlcNAc_2_ at glycan site N331, derived from the tryptic digest of gp120 Z1800M. This glycosite is part of the high mannose patch.


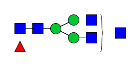

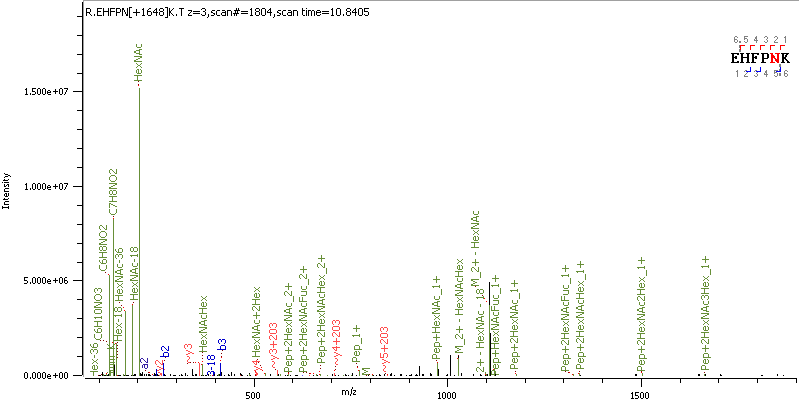


**Figure T.** Annotated HCD MS2 spectrum of N-glycopeptide ^343^EHFPNK^348^, with the most abundant glycoform GlcNAc_3_Fuc_1_Man_3_GlcNAc_2_ at glycan site N347, derived from the tryptic digest of gp120 Z1800M.


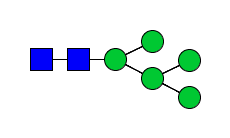

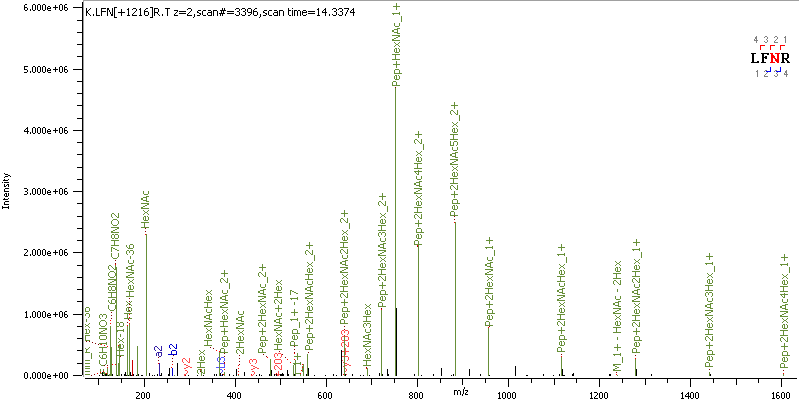


**Figure U.** Annotated HCD MS2 spectrum of N-glycopeptide ^381^LFNR^384^, with the most abundant glycoform Man_5_GlcNAc_2_ at glycan site N383, derived from the tryptic digest of gp120 Z1800M.


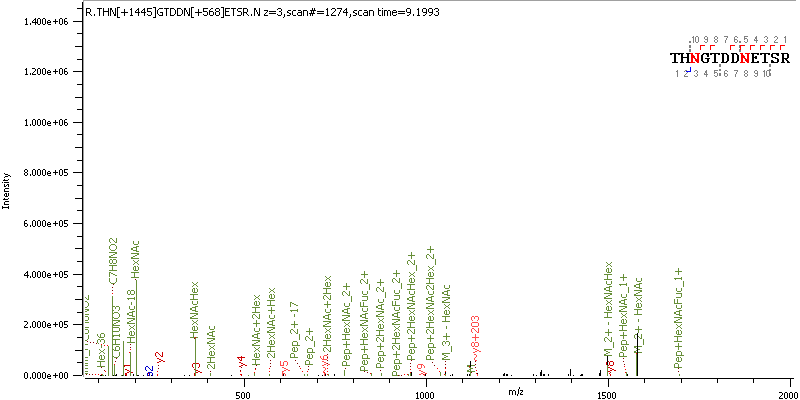


**Figure V.** Annotated HCD MS2 spectrum of N-glycopeptide ^385^THNGTDDNETSR^397^, which was only found in conjunction of both N-glycosylation motifs in all three enzymatic digests. Therefore, no confident glycoform assignment for glycosites N387 and N392 was possible. However, the MS data indicated glycosylation on both sites. For glycan modelling, the glycoform Man_5_GlcNAc_2_ was assigned to both.


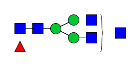

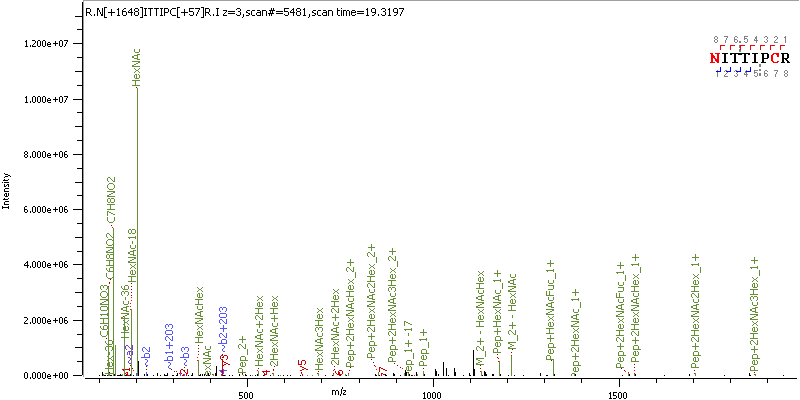


**Figure W.** Annotated HCD MS2 spectrum of N-glycopeptide ^397^NITTIPCR^404^, with carbamidomethylation of the cysteine (C) and the most abundant glycoform GlcNAc_3_Fuc_1_Man_3_GlcNAc_2_ at glycan site N397, derived from the tryptic digest of gp120 Z1800M.


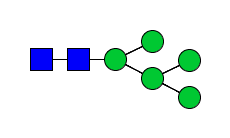

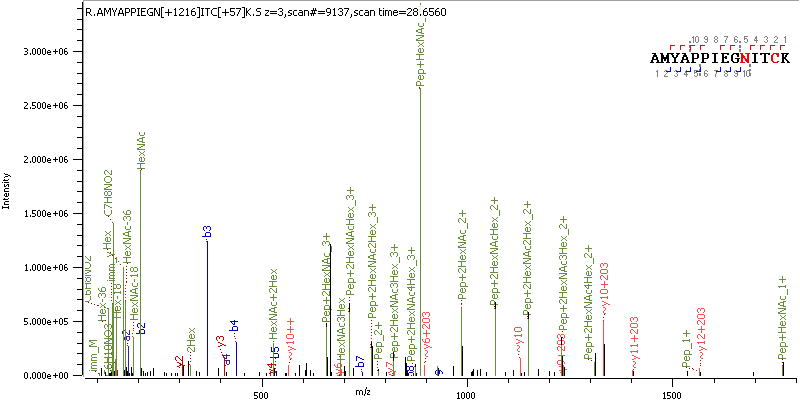


**Figure X.** Annotated HCD MS2 spectrum of N-glycopeptide ^418^AMYAPPIEGNITCK^431^, with carbamidomethylation of the cysteine (C) and the most abundant glycoform Man_5_GlcNAc_2_ at glycan site N427, derived from the tryptic digest of gp120 Z1800M.


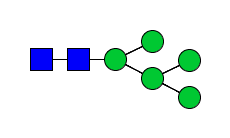

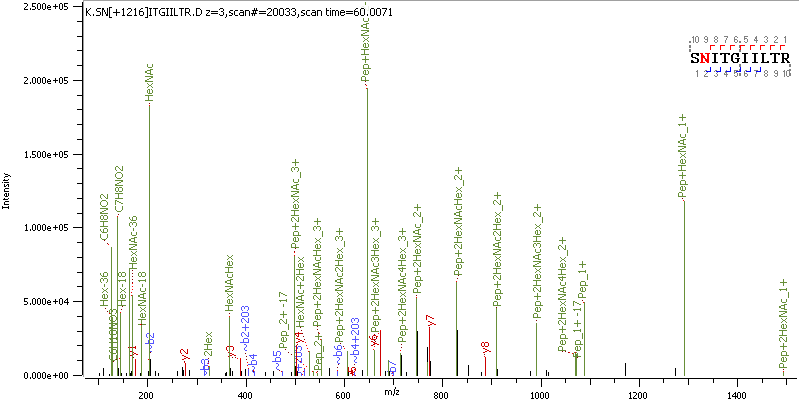


**Figure Y.** Annotated HCD MS2 spectrum of N-glycopeptide ^432^SNITGIILTR^441^, with the most abundant glycoform Man_5_GlcNAc_2_ at glycan site N433, derived from the tryptic digest of gp120 Z1800M.


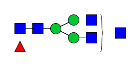

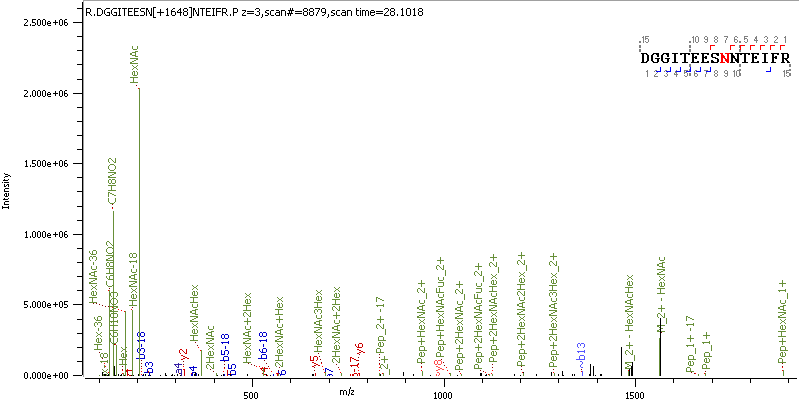


**Figure Z.** Annotated HCD MS2 spectrum of N-glycopeptide ^442^DGGITEESNNTEIFR^456^, with carbamidomethylation of the cysteine (C) and the most abundant glycoform GlcNAc_3_Fuc_1_Man_3_GlcNAc_2_ at glycan site N450, derived from the tryptic digest of gp120 Z1800M.


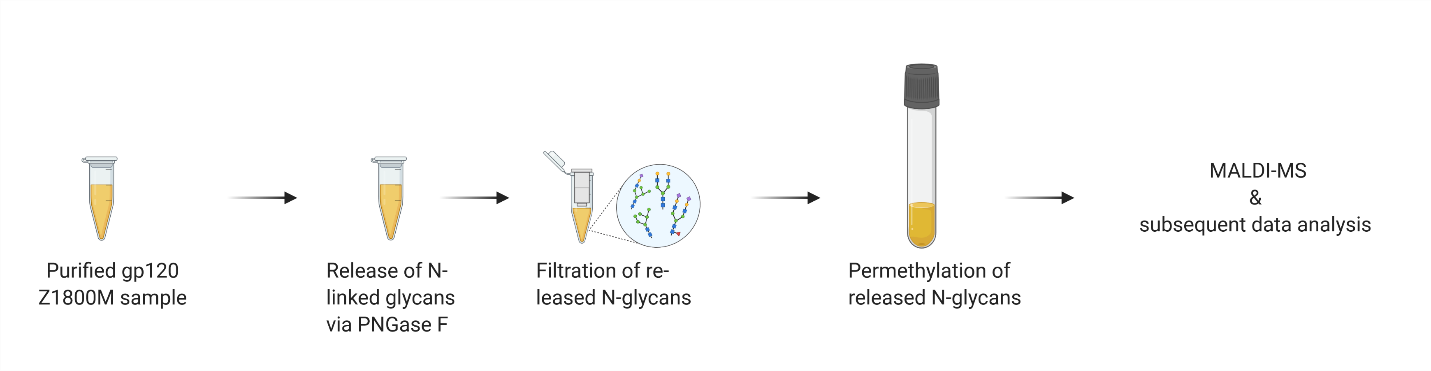


**Figure AA.** Glycomic workflow applied to the monomeric gp120 Z1800M. The sample preparation was performed as described by [1]. (created with BioRender.com)

**Figure AB.** Full MALDI TOF/TOF mass spectrum of released and permethylated N-linked glycans of gp120 protein Z1800M. The identified N-glycans are coherent with the results from the glycoproteomic analysis. The sample preparation was performed as described by [1].

**References**

1. Shajahan A, Heiss C, Ishihara M, Azadi P. Glycomic and glycoproteomic analysis of glycoproteins-a tutorial. Anal Bioanal Chem. 2017;409(19):4483-505. Epub 2017/06/07. doi: 10.1007/s00216-017-0406-7. PubMed PMID: 28585084; PubMed Central PMCID: PMCPMC5498624.
